# Music interventions to improve women’s health outcomes in the preconception, antepartum, intrapartum, and postpartum periods: An overview of reviews

**DOI:** 10.1371/journal.pone.0339337

**Published:** 2026-02-18

**Authors:** Meighan Mary, Briana Kramer, Kirantheja Daggula, Anqi He, Sarah Clifford, Elizabeth Stierman, Kathryn Spielman, Porcia Manandhar, Andreea A. Creanga

**Affiliations:** 1 IMPROVE Maternal Health Data Innovation and Coordination Hub, Johns Hopkins Bloomberg School of Public Health, Baltimore, Maryland, United States of America; 2 International Center for Maternal and Newborn Health, Johns Hopkins Bloomberg School of Public Health, Baltimore, Maryland, United States of America; 3 International Health Department, Johns Hopkins Bloomberg School of Public Health, Baltimore Maryland, United States of America; 4 Department of Gynecology and Obstetrics, Johns Hopkins School of Medicine, Baltimore, Maryland, United States of America; Group for Technical Assistance / Asian College for Advance Studies, Purbanchal University, NEPAL

## Abstract

**Aim:**

To synthesize evidence from systematic reviews of interventions that employ music to improve women’s health outcomes in the preconception, antenatal, intrapartum, and postpartum periods.

**Methods:**

Systematic reviews published between 2010 and 2025 that addressed music interventions for women in the preconception, antenatal, intrapartum, and postpartum periods were sourced from MEDLINE, Scopus, Embase, PsychINFO, and Cochrane Database of Systematic Reviews in March 2025. 754 systematic reviews were imported into Covidence software. Title-abstract screening, and full text review were conducted in duplicate by a team of 5 screeners. Data were extracted to summarize key characteristics and meta-analysis results. The methodological quality of the included reviews was assessed using the AMSTAR-2 tool. Risk of bias and GRADE quality assessments were extracted; when not reported, MM and BK assessed the risk of bias and quality of evidence.

**Results:**

Aggregated findings from 20 reviews suggest that music interventions in the preconception, antepartum, and intrapartum periods have potential to alleviate anxiety (SMD ranging from −7.0 to −0.21), depression (SMD −3.67 to −0.51), and pain (SMD −2.70 to −0.92). Effects from implementation in the postpartum period appear more limited, albeit with a notable exception of demonstrated benefit in reducing depressive symptoms (SMD:-0.75; 95%CI:-1.47,-0.03). Interpretation of these findings warrant caution due to important methodological limitations related to widespread bias, heterogeneity, and imprecision in effect estimation.

**Conclusions:**

Music interventions are a promising approach for women across the perinatal continuum of care. However, significant concerns with the methodological rigor of existing studies need to be addressed before implementation in clinical settings. Further research is critical to identifying design characteristics and implementation modalities of music interventions that most effectively improve women’s health outcomes. Strengthening the evidence on music interventions is vital to informing the effective integration of complementary and alternative medicine approaches into person-centered care strategies for women’s health.

## Introduction

The use of complementary and alternative medicine (CAM) and the pursuit of wellness are common among women in the United States across the life course. According to a nationally representative study, 67% of reproductive-aged women reported using CAM within the year prior [1]. Across all women, pain and anxiety were the chief complaints being addressed by CAM, but among pregnant and postpartum women, pregnancy-related complaints were the primary indication for CAM use. According to the same study, among pregnant women, mind-body approaches were more frequently used than biological therapies (e.g., herbal medicine). A 2014 cross-sectional survey of postpartum women also documented that over two thirds reported CAM use [2]. Trends in the growth of the wellness industry among women suggest these estimates will not decrease. The wellness economy in the US is already the largest in the world at $2 trillion annually [3] and experiencing growth among women, who spend the most on products tied to reproductive health [4]. Given this demonstrated appetite for complementary and alternative therapies and products that promote holistic wellbeing, there is a need for rigorous review of the evidence on popular and promising approaches towards the goal of establishing their safety and effectiveness during the perinatal period.

One promising approach to improving holistic health during the perinatal period is the use of music interventions. Music stimulates brain regions involved in emotional processing (e.g., nucleus accumbens, amygdala, insular and orbitofrontal cortices) and influences physiological responses including salivary and serum cortisol excretion, heart rate, and perceived stress [5,6]. Music interventions consist of music therapy, which is delivered by a licensed therapist, usually in a personalized fashion, and music medicine or listening, which is generally pre-recorded and may be self-administered or administered by a variety of health care professionals without special training [7]. They have the benefit of being widely available and low-cost, with music listening having the lowest barriers to access and application. Because of this, researchers have not hesitated to recommend music interventions for perinatal populations despite unclear evidence [8].

While there have been numerous systematic reviews evaluating the effectiveness of music interventions in pregnant and postpartum women over the past two decades, the narrow scope of their populations, interventions, and outcomes made it challenging to synthesize their evidence and develop recommendations for use across the perinatal continuum of care. For example, existing reviews have focused on anxiety during infertility treatment, antenatal sleep quality, pain during labor, pain and anxiety during cesarean delivery, postpartum depression, and breastmilk production [9–13]. Because of the fragmentary state of the evidence, there is a need for an overview of reviews to inform the various stakeholders interested in the use of music interventions and, more broadly, complementary and alternative approaches in pregnancy and postpartum. This study synthesizes the evidence from systematic reviews of interventions that employ music to improve women’s health outcomes in the preconception, antenatal, intrapartum, and postpartum periods.

## Sources

Our review followed Cochrane guidance for overview of reviews [14] and was registered in the Prospective Register of Systematic Reviews (CRD420251031216). We conducted a broad search of the MEDLINE, Scopus, Embase, PsychInfo and Cochrane Database of Systematic Reviews in March 2025. A draft search strategy using Boolean terms ‘AND’ and ‘OR’ to separate search words and terms was developed and piloted with two primary concepts (i.e., music and women in preconception, antepartum, intrapartum, and postpartum periods) and adapted as needed (See S1 Table). The eligibility criteria were as follows:

- Population: Women in the preconception, antenatal, intrapartum, and postpartum periods (up to one year after the end of pregnancy)
- Intervention: Any music intervention (e.g., music listening or music therapy) employed alone or in combination with other interventions
- Comparison: Any other intervention, treatment, or routine care/no intervention
- Outcomes: Any women’s health outcome
- Study type: Systematic reviews of peer-reviewed randomized or quasi-randomized controlled trials sourced from >1 databases, with clearly defined eligibility criteria and systematic screening and data extraction procedures
- Language: Literature published in any language

The literature search was restricted within a timeline between 2010–2025 to capture evidence since the most recent relevant Cochrane review focused on music interventions during childbirth [15]. We retrieved all relevant published systematic reviews, reviewed citation lists, and identified registered and published protocols to cross-check for any reviews that may be missing or in process.

## Study selection

The screening team was comprised of five screeners. All retrieved literature was imported to Covidence software [16] whereby duplicate documents were excluded at the onset of screening. Title and abstract screening was conducted in duplicate for each article. MM resolved any discrepancies or conflict upon completion. Subsequent full article screening was conducted following a similar protocol of resolution of discrepancies once all literature had been reviewed by two screeners. All literature excluded from the full text review was documented in Covidence and stored separately (S2 Table).

The primary studies of included reviews were mapped in a citation matrix to assess the extent and nature of overlap in reviews (S3 Table). We employed Pollack et al.’s (2019) guidance and decision tool for handling overlapping systematic reviews [17]. Reviews were excluded if their primary studies were completely over-lapping by a 1.) more recent review or 2.) review of higher quality (as described below). All such exclusions are reported in S2 Table.

### Data extraction and management

A team of four research team members independently extracted the data in duplicate using the Covidence software. Discrepancies were reviewed by MM. Data were extracted using a piloted form designed to summarize key characteristics of each review. Key data points included the objectives of each review, inclusion criteria (e.g., participants, details of intervention, comparison, outcomes, type of trials and length of follow-up), date of last search, number of included trials, number of participants for each comparison and statistical outcome data, and narrative text of the results. Data were subsequently synthesized in a series of summary tables including details of the quality assessment of individual reviews.

MM and BK assessed the methodological quality of the included reviews using the 16 domains detailed in the AMSTAR 2 tool [18] and rated the overall confidence in the reviews’ quality. Overall confidence was assessed using the following guidance: High (0–1 non-critical weakness), Moderate (>1 non-critical weakness), Low (1 critical flaw with or without non-critical weaknesses), and Critically low (>1 critical flaw with or without non-critical weaknesses). Established AMSTAR-2 guidance [18] defined the following criteria as critical: establishment of a priori review methodology, utilization of a comprehensive literature search strategy, description of included studies, utilization of a satisfactory technique for assessing risk of bias (RoB), appropriate methods for statistical analysis of combined results, and consideration of RoB in the interpretation of results. Reviews were not excluded based on their methodological quality, nor were sensitivity analyses conducted to explore the consequences of combining reviews of varying methodological quality. Data related to the domain-specific or overall quality and RoB assessments for primary studies were extracted from each eligible systematic review. When different tools were employed across reviews to report RoB (e.g., JBI critical appraisal tool, QATQS, SIGN), MM and BK synchronized common elements to the Cochrane RoB tool and consulted the original primary studies to extract any needed information related to missing quality or RoB domains.

When available, the Grading of Recommendations Assessment, Development and Evaluation (GRADE) assessments [19] were extracted from the eligible reviews to understand the quality of evidence for each reported outcome. When not reported, MM and BK independently assessed the quality using GRADE guidance and any discrepancies were discussed to finalize the rating.

### Data synthesis

The unit of analysis was the eligible systematic review. Statistical outcome data are presented as reported in each review; when unavailable, data are presented as a narrative synthesis. A synthesis table was developed to include key characteristics of each review and primary outcomes reported. Data from reviews were disaggregated into subgroups based on the intervention period (preconception, antepartum, intrapartum, and postpartum periods), intervention type (music listening vs. music therapy with active engagement with a music therapist or other mediums), and outcomes measured to glean insights across the spectrum of maternal health care. Meta-analyses that combined trials from different periods were excluded due to significant heterogeneity in intervention approach and outcome assessment. To mitigate the presentation of multiple meta-analysis comparisons with over-lapping primary studies, we only reported the results of the most recent and/or comprehensive meta-analysis conducted for relevant comparisons. For example, if review 1 presented a meta-analysis with primary studies “a,” and “b” for a given comparison and review 2 assessed the comparison with primary studies “a,” “b,” “c,” and “d”, review 1’s meta-analysis results were excluded for the indicated comparison assessment. Sub-group analyses investigating specific intervention characteristics were presented alongside main meta-analyses.

## Results

**Fig 1:**
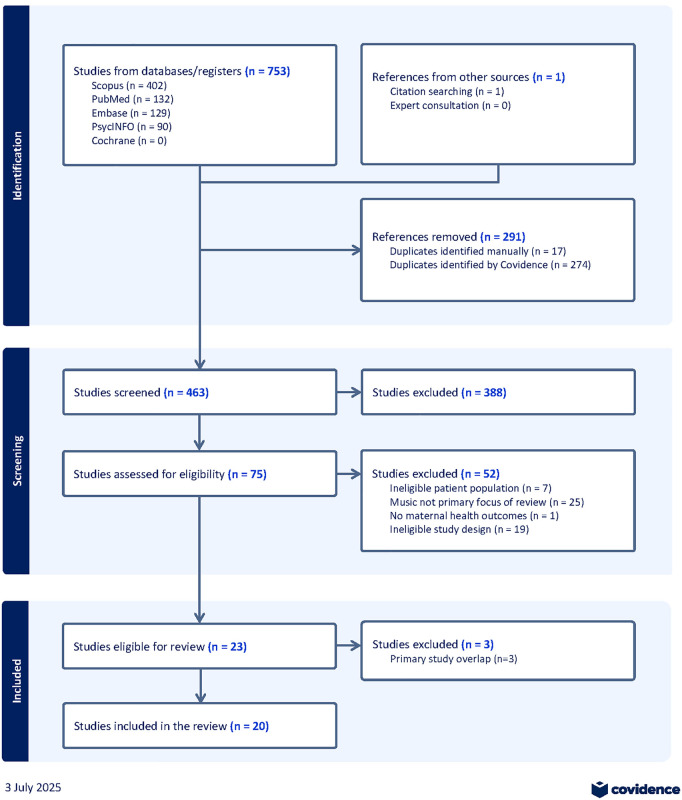
PRISMA diagram.

We retrieved 753 studies across the five databases and identified one systematic review via citation searching, of which 291 were duplicates and 388 were deemed irrelevant from title and abstract screening (Fig 1). A full text review was conducted for the remaining 75, with 52 reviews deemed ineligible and an additional 3 reviews excluded due to primary studies overlapping with other reviews. In total, 20 systematic reviews were included in our overview of reviews [8–13,20–33].

Appraisal of systematic review registries identified 12 eligible protocols (S4 Table). Among these registered protocols, we identified three published systematic reviews: two were already included in our overview [21,24], and one had a published abstract [34]. As such, no additional reviews were added to the final overview of reviews.

### Description of included reviews

Table 1 describes the key characteristics of the reviews. All were published between 2017 and 2025. Meta-analyses were conducted in 15 reviews [9–13,21,22,24–29,32,33]; Maul et al. (2024) synthesized the literature and calculated the reversed Cohen’s d for a set of a priori outcome measures [20]; and four presented only narrative syntheses [8,23,29–31]. All but three reviews included only RCTs. Overall, the reviews included 132 trials and 16,390 participants; 83 trials were included in only one review, while 49 were included in multiple reviews (ranging from 2 to 7; see S5 Table).

**Table 1:** Characteristics of included reviews (N = 20).

Review	Interventions	Population	Comparator	Maternal Health Outcomes	Date of search	Types of studies included	No. of studies included	Total no. of subjects
*Preconception interventions (n = 1)*								
Kızılkaya 2024 [9]	Mixed music interventions	Women who were diagnosed with infertility, underwent fertility treatment or procedures, and attended fertility clinics	No intervention	Anxiety Pain Pregnancy rates Vital signs	May 2024	RCT	8	1634
*Antepartum interventions (n = 5)*								
Hoffmann 2025 [10]	Music listening	Pregnant women suffering from insomnia	No intervention	Sleep Quality	March 2024	RCT	4	348
Dogan-Gangal 2024 [8]	Mixed music interventions	Pregnant women	No intervention	Anxiety Blood pressure Depression Sleep quality Stress	January 2019	RCT and CCT	13	2143
Maul 2024 [20]	Mixed music interventions	Pregnant women	Routine antenatal care, conventional treatment of hypertension, and/or resting	Anxiety Blood pressure Depression Sleep quality Stress	July 2023	RCT	14	2375
Lin 2019 [21]	Mixed music interventions	Pregnant women	No intervention	Anxiety	September 2019	RCT	11	1482
Corbijn van Willenswaard 2017 [22]	Mixed music interventions	Pregnant women	No intervention	Anxiety Stress	April 2016	RCT and CCT	5	1261
*Intrapartum interventions (N = 6)*								
Chehreh 2023 [11]	Music listening during labor	Pregnant women	Control -not specified	Pain	November 2022	RCT	12	1168
Hunter 2023 [23]	Music listening during vaginal births and caesarean delivery	Pregnant women	Control -not specified	Anxiety Labor duration Pain	March 2023	RCT	28	2835
Şen 2023 [24]	Music listening during labor	Pregnant women	No intervention	Labor duration Pain	November 2022	RCT	12	1168
Chuang 2019 [25]	Music listening during labor	Primiparous pregnant women expected to have uncomplicated vaginal deliveries	No intervention	Anxiety Pain	July 2017	RCT and CCT	5	392
Maleki and Youseflu 2023 [26]	Music listening after episiotomy	Healthy women who had a vaginal delivery	No intervention	Pain	July 2022	RCT	7	677
Weingarten 2021 [12]	Music listening before, during, and immediately after cesarean delivery	Pregnant women	No intervention	Anxiety	November 2020	RCT	15	1361
*Postpartum interventions (n = 3)*								
Yang 2019 [28]	Music listening	Women diagnosed with postpartum depression	Standard treatment, including drug treatment, psychological treatment, and health education.	Anxiety Depression Maternal attachment Pain Satisfaction Sleep assessments	November 2018	RCT	7	1065
Hakimi 2021 [27]	Mixed music interventions	Postpartum women	No intervention	Anxiety Pain Stress	March 2019	RCT	4	395
Düzgün and Özer 2020 [13]	Mixed music interventions	Breastfeeding mothers	No intervention	Amount of breast milk	March 2020	RCT	5	554
*Mixed: antepartum, intrapartum, and/or postpartum (n = 5)*								
Wu 2020 [33]	Five elements music listening alone or in combination with other treatment	Pregnant and postpartum women	Routine care or psychiatric nursing care	Anxiety Depression Labor duration Pain Blood loss Maternal venous blood content	January 2020	RCT	13	2387
Han 2024 [29]	Mixed music interventions	Pregnant and postpartum women	Routine care including health education and psychological care	Anxiety Depression Pain Satisfaction Sleep	September 2024	RCT	13	1540
Ji 2024 [30]	Mixed music interventions	Pregnant women	Control -not specified	Anxiety Blood Pressure Pain Sleep quality	December 2022	RCT	17	2232
Shafqat 2024 [31]	Mixed music interventions	Pregnant women	Control -not specified	Anxiety	December 2023	RCT	33	4363
Sun 2024 [32]	Mixed music interventions	Pregnant or postpartum women	No intervention	Depression	May 2024	RCT	10	988

Abbreviations: CCT: controlled clinical trial; RCT: randomized controlled trial.

Reviews varied widely by intervention type, intervention period, comparators, and outcome measures. We categorized music interventions as 1.) music listening, 2.) music therapy interventions (with guided active engagement with music (singing, dancing, etc.), and 3.) mixed (combination of music listening with music therapy interventions). Nine of the reviews included only trials with music listening interventions [10–12,23–26,28,33], with the remaining including trials with a mix of music listening and music therapy approaches. Despite reference to music therapy in name, no trials assessed only music therapy approaches. Reviews covered music intervention trials that spanned preconception (n = 1), antepartum (n = 6), intrapartum (n = 5), and postpartum (n = 3) periods, with five reviews including trials across several periods. Kızılkaya et al.’s (2024) review in the preconception period focused on music listening and music therapy interventions employed during infertility treatment and assessed their effect on anxiety, pain, pregnancy rates, and vital signs [9]. Reviews of antepartum music interventions assessed the effect of both music listening and music therapy approaches on pregnant women’s anxiety [8,20–22], stress [8,20,22], depression [8,20], blood pressure [8,20], and sleep [8,10,20]. Reviews assessing intrapartum interventions examined the effect of music listening during labor [11,23–25], after an episiotomy [26], and before, during, and/or immediately after cesarean deliveries [12,23]. Reviews on music interventions in the postpartum period varied in approach: Hakimi et al. (2021) synthesized the effect of music listening and music therapy approaches on postpartum anxiety and pain [27], while Yang et al. (2019) examined the effect of music listening on a variety of outcomes among women diagnosed with postpartum depression [28] and Düzgün and Özer (2020) assessed the impact of music listening and music therapy approaches on women’s milk production [13]. Several reviews investigated the effect of music listening and music therapy approaches on maternal health outcomes more broadly spanning the antepartum and postpartum periods [29–33]. Shafqat (2024) and Sun (2024) focused on music interventions’ effects on specific outcomes (i.e., anxiety [31] and depression [32]), and Wu et al. (2020) synthesized findings from trials employing “Five Elements Music” listening [33] – an East Asian traditional medicine theory employing five tones (Jue, Zhi, Gong, Shang, and Yu).

Music interventions were compared to no intervention in 12 reviews [8–10,12,13,21,22,24–27,32], routine care or treatment (i.e., for depression or hypertension) in two reviews [20,28], and routine or psychological care in two reviews [29,33]. Four of the narrative reviews did not specify the type of control group employed in the primary studies [11,23,30,31].

### Methodological quality

Unweighted AMSTAR-2 scores ranged from 5 [30] to 14 [9,13], with a mean score of 9.9 out of 16. However, when considering critical components of the AMSTAR-2 tool, all reviews were rated as having critically low confidence in the quality (See S6 Table), with the exception of Maul et al. (2024) that ranked as moderate [20] and Kızılkaya et al. (2024), Weingarten et al. (2021), and Düzgün and Özer (2020) that ranked as low [9,12,13]. RoB of primary studies included in each review were high with concerning levels of performance and detection bias (S7 Table). While two thirds of primary studies had low RoB for random sequence generation (67%), only half (50%) had low risk for allocation concealment. Approximately one eighth (13%) and one third (31%) of primary studies had low risk for blinding of participants and study personnel and blinding of outcome assessments, respectively. Less than half of the primary studies (48%) had low RoB for selective outcome reporting.

### Effect of music interventions on maternal health outcomes

Three primary outcomes were commonly assessed across reviews: anxiety, depression, and pain. Outcome data were available for 20 comparisons to assess the effect of music interventions on anxiety (Table 2), 8 comparisons for depression (Table 3), and 8 comparisons for pain (Table 4; See S8–S10 Tables for details on GRADE assessments). Data on the effect of music interventions on other outcomes (e.g., stress, sleep, vital signs, etc.) are detailed in S11–S16 Tables.

**Table 2:** Summary of effects of music interventions on anxiety, disaggregated by intervention period.

Comparison	Outcome measurement	No. of subjects (trials)	Measure of Effect (95% CI)	Direction of effect	*I*^2^ (%)	Quality of evidence (GRADE)	Primary studies
*Preconception interventions*							
Mixed music interventions before or during ART treatment vs. no intervention	Anxiety: Composite measure derived from: DASS 21, STAI, VAS	902 (8)	SMD: −0.27 (−0.40, −0.14)	Favors music interventions	44%	Low	[35–42]
*Antepartum interventions*							
Music listening vs. no intervention	Anxiety: STAI	469 (2)	SMD: −0.21 (−0.39, −0.03)	Favors listening to music	0%	Low	[43,44]
Music listening at home vs. no intervention ^a^	Anxiety: STAI	393 (3)	SMD: −0.28 (−0.47, −0.08)	Favors listening to music at home	0%	Low	[43,45,46]
Music listening during antepartum hospitalization for HDP vs. no intervention ^a^	Anxiety: STAI	130 (2)	SMD: −0.54 (−2.09, 1.01)	NS	94%	Very low	[47,48]
Music listening during antepartum procedures vs. no intervention ^a,b^	Anxiety: STAI	711 (3)	SMD: −0.43 (−1.21, 0.34)	NS	96%	Very low	[49–51]
Music listening with pre-selected music vs. no intervention ^a^	Anxiety: STAI	445 (2)	SMD: −0.83 (−1.50, −0.17)	Favors listening to pre-selected music	74%	Very low	[45,49]
Music listening with patient chosen music vs. no intervention ^a^	Anxiety: STAI	789 (6)	SMD: −0.29 (−0.68, 0.09)	NS	85%	Very low	[43,46–48,50,51]
Mixed music interventions vs. no intervention	Anxiety: STAI	1234 (8)	SMD: −0.42 (−0.83, −0.02)	Favors music interventions	91%	Very low	[43,45–51]
*Intrapartum interventions*							
Music listening during labor vs. no intervention	Anxiety: SAS	302 (4)	SMD: −0.96 (−1.15, −0.76)	Favors listening to music	0%	Low	[52–54]
Music listening before cesarean delivery vs. no intervention	Preoperative anxiety: STAI	250 (4)	MD: −3.95 (−9.07, 1.18)	NS	22%	Moderate	[55–58]
	Preoperative anxiety: SAS	60 (1)	MD: −7.0 (−8.43, −5.57)	Favors listening to music	NA	Low	[59]
Music listening during cesarean delivery vs. no intervention	Intraoperative anxiety: VAS	368 (2)	MD: −0.54 (−0.87, −0.2)	Favors listening to music	0%	Moderate	[60,61]
	Intraoperative anxiety: SAS	64 (1)	MD: −4.8 (−7.08, −2.52)	Favors listening to music	NA	Low	[62]
	Intraoperative anxiety: STAI	304 (1)	MD: −2.8 (−4.57, −1.03)	Favors listening to music	NA	Low	[61]
	Postoperative anxiety: SAS	64 (1)	MD: −4.5 (−6.82, −2.18)	Favors listening to music	NA	Low	[62]
Music listening during and/or immediately after cesarean delivery vs. no intervention	Postoperative anxiety: VAS	847 (7)	MD: −0.38 (−0.53 to −0.23)	Favors listening to music	0%	Moderate	[60,61,63–67]
Music listening before, during, and/or immediately after cesarean delivery vs. no intervention	Postoperative anxiety: STAI	504 (4)	MD: −2.1 (−6.38, −2.18)	Favors listening to music	58%	Low	[58,61,68,69]
Music listening before scheduled cesarean delivery vs. no intervention ^a^	Preoperative anxiety: STAI	125 (2)	MD: 0.63 (−4.62, 5.89)	NS	48%	Low	[56,58]
Music listening before and/or during scheduled cesarean delivery vs. no intervention ^a^	Postoperative anxiety: STAI	200 (3)	MD: −2.22 (−7.44, 2.99)	NS	15%	Low	[58,68,69]
Music listening during and/or after scheduled cesarean delivery vs. no intervention ^a^	Postoperative anxiety: VAS	361 (5)	MD: −0.49 (−0.85, −0.13)	Favors listening to music	18%	Moderate	[60,63,64,66,67]
*Postpartum interventions*							
Music listening vs. kangaroo care, traditional treatment, or psychological treatment	Anxiety: Composite continuous measure derived from: STAI, SAS, VAS	601 (3)	SMD: −1.26 (−2.81, 0.29)	NS	98%	Very low	[70–72]
Music listening vs. kangaroo care ^a, d^	Anxiety: Composite binary measure derived from STAI, SAS	460 (2)	RR: 0.38 (0.09, 1.63)	NS	0%	Low	[71,72]
Five elements music listening alone or in combination with other treatment vs. no intervention	Anxiety rate	524 (2)	OR: 0.42 (0.19, 0.94)	Favors listening to five elements music	0%	Low	[71,73]
Mixed music interventions vs. no intervention	Anxiety 0.5–8hrs post delivery: VAS-A	318 (3)	MD: −0.96 (−2.26, 0.34)	NS	92%	Very low ^c^	[64,70,74]
	Anxiety 2 weeks postpartum: VAS-A	77 (1)	MD: 0.84 (−1.59, 3.27)	NS	N/A	Very low ^c^	[75]

Acronyms: ART: assisted reproductive technology; BAI: Beck Anxiety Inventory; DASS21: Depression, Anxiety and Stress Scale; HDP: Hypertensive disorders of pregnancy; MD: Mean difference; N/A: Not available; NS: Not significant; OR: Odds ratio; RR: Risk ratio; SAS: Self-rating Anxiety Scale; SMD: Standardized mean difference; STAI: State-Trait Anxiety Inventory; VAS-A: Visual analogue scale for anxiety.

Notes: ^a^ Sub-group analyses presented; ^b^ Antepartum procedures include non-stress tests and manual vacuum aspiration for abortion; ^c^ GRADE ratings were calculated by Hakimi 2021. ^d^ Kangaroo care, or skin-to-skin contact between mother and baby, is a technique used with premature and low birth weight babies to regulate the baby’s heart rate, body temperature, and breathing. In the referenced studies, authors compared the effect of music listening to kangaroo care on maternal anxiety.

**Table 3:** Summary of effects of music interventions on depression, disaggregated by intervention period.

Comparison	Outcome measurement	No. of subjects (trials)	Measure of Effect (95% CI)	Direction of effect	*I*^2^ (%)	Quality of evidence (GRADE)	Primary studies
*Antepartum interventions*							
Five elements music listening alone or in combination with other treatment vs. no treatment	Depression: SDS	1138 (6)	SMD: −3.67 (−5.21, −2.13)	Favors listening to five elements music	99%	Very low	[76–81]
Mixed music interventions vs. no intervention	Depression: Composite measure derived from EPDS, BDI, and HAM-D	703 (6)	SMD: −0.51 (−0.80, −0.22)	Favors music interventions	67%	Very low	[43,45,48,70,82,83]
	Depression: EPDS	374 (3)	SMD: −0.44 (−0.90, 0.02)	NS	74%	Very low	[45,84,85]
*Postpartum interventions*							
Music listening vs. no intervention	Depression: Composite measure derived from EPDS, BDI, and HAM-D	285 (4)	SMD: −0.75 (−1.47, −0.03)	Favors listening to music	87%	Very low	[82,86–88]
Music listening vs. psychological treatment and Chinese medicine	Depression: SDS	80 (1)	RR: 0.73 (0.63, 0.86)	Favors listening to music	N/A	Low	[89]
Music listening vs. psychological treatment, drug treatment, and/or traditional treatment	Depression: Composite continuous measure derived from EPDS, HAM-D, BAI, and SDS	763 (4)	SMD: −0.87 (−1.23, −0.51)	Favors listening to music	79%	Very low	[70,71,90,91]
Music listening vs. health education, psychological treatment, and/or traditional treatment	Depression: Composite binary measure derived from EPDS and SDS	623 (4)	RR: 0.25 (0.12, 0.54)	Favors listening to music	0%	Low	[70,71,92]
Five elements music listening alone or with psychiatric nursing vs. no intervention	Depression: EPDS	240 (2)	SMD: −0.84 (−1.33,-0.35)	Favors listening to five elements music	71%	Low	[93,94]
Mixed music interventions vs. no intervention	Depression: EPDS	906 (6)	SMD: −0.59 (−0.96, −0.21)	Favors music interventions	87%	Very low	[43,70,82,86,87,95]

Acronyms: BDI: Beck Depression Inventory; EPDS: Edinburgh postnatal depression scale; HAM-D: Hamilton Depression Rating Scale; HIC: High income countries; LMIC: Low-and-middle income countries; N/A: Not available; NS: Not significant; RR: Risk ratio; SDS: Self-raiting depression scale; SMD: Standardized mean difference.

Notes: ^a^ Sub-group analyses presented.

**Table 4:** Summary of effects of music interventions on pain, disaggregated by intervention period.

Comparison	Outcome measurement	No. of subjects (trials)	Measure of Effect (95% CI)	Direction of effect	*I*^2^ (%)	Quality of evidence (GRADE)	Primary studies
*Preconception interventions*							
Music listening before or during assisted reproductive technology treatment vs. no intervention	Pain: VAS-P	302 (3)	MD: −0.96 (−1.72, −0.20)	Favors listening to music	0%	Low	[39,41,42]
*Intrapartum interventions*							
Music listening during labor vs. no intervention	Overall Pain: VAS-P	332 (4)	MD: −0.92 (−1.33, −0.51)	Favors listening to music	45%	Moderate	[52–54,96]
	Pain in active phase of labor: VAS-P	912 (16)	SMD: −1.26 (−1.52, −1.01)	Favors listening to music	67%	Moderate	[52,54,97–102]
	Pain 2hrs after initiation of music: VAS-P	528 (7)	SMD: −1.22 (−1.67, −0.76)	Favors listening to music	81%	Low	[52–54,97,99–101]
	Pain 3hrs after initiative of music: VAS-P	312 (5)	SMD: −1.06 (−1.32, −0.81)	Favors listening to music	12%	High	[53,97,99–101]
Music listening during labor with preselected music vs. no intervention ^a^	Pain: VAS-P	860 (14)	SMD: −1.22 (−1.50, −0.95)	Favors listening to pre-selected music	69%	Moderate	[53,97,99,101–103]
Music listening during labor with headphones vs. no intervention ^a^	Pain: VAS-P	1418 (20)	SMD: −1.04 (−1.27, −0.81)	Favors listening to music with headphones	75%	Moderate	[52–54,97,99,100,103]
Music listening during labor via speakers vs. no intervention ^a^	Pain: VAS-P	200 (4)	SMD: −0.44 (−0.93, 0.05)	NS	65%	Low	[52,98,103]
Music listening after episiotomy vs. no intervention	Episiotomy pain measured at any time: VAS-P	677 (7)	SMD = −1.60 (−2.18, −1.02)	Favors listening to music	96%	Very Low	[54,70,98,104–107]
	Episiotomy pain 1hr post-procedure:: VAS-P	425 (4)	SMD: −1.78 (−3.1, −0.46)	Favors listening to music	96%	Very low	[54,98,104,105]
	Episiotomy pain 2hrs post-procedure: VAS-P	232 (2)	SMD: −2.70 (−5.21, −0.19)	Favors listening to music	98%	Very low	[54,107]
	Episiotomy pain 3–24hrs post-procedure: VAS-P	241 (2)	SMD: −2.09 (−2.71, −1.47)	Favors listening to music	88%	Very low	[54,70,107]
	Episiotomy pain 24hrs post-procedure: VAS-P	242 (2)	SMD: −1.71 (−4.49, 1.07)	NS	98%	Very low	[54,98]
	Episiotomy pain 48hrs post-procedure: VAS-P	100 (1)	SMD: −0.16 (−0.88, 0.55)	NS	NA	Very low	[98]
Music listening before, during, and immediately after cesarean delivery vs. no intervention	Postoperative pain: VAS-P	479 (5)	MD: −0.82 (−1.74, 0.11)	NS	86%	Very low	[59,63–65,67]
*Postpartum Interventions*							
Music listening vs. no intervention	Postpartum Pain (30–60 mins after delivery): VAS-P	318 (3)	MD: −1.85 (−3.96, 0.26)	NS	98%	Very low	[64,70,74]

Acronyms: MD: Mean difference; NS: Not significant; RR: Risk ratio; SMD: Standardized mean difference.

Notes: ^a^ Sub-group analyses presented.

#### Effect of music interventions on anxiety.

Mixed music interventions were found to significantly reduce anxiety when implemented in the preconception period (8 trials with 902 women; SMD = −0.27; 95% CI: −0.40, −0.14) and antepartum period (8 trials with 1234 women; SMD: −0.42; 95% CI: −0.83, −0.02) compared to no intervention, albeit with low and very low quality of evidence, respectively. In addition, no significant effect on anxiety was reported when assessing mixed music interventions implemented in the postpartum period.

The effect of music listening on anxiety was variable. Low quality evidence from two trials (469 women) found that music listening during the antepartum period significantly reduced anxiety compared to no intervention (SMD: −0.21; 95% CI: −0.39, −0.03). However, when looking at subgroup analyses, listening to music at home during pregnancy significantly reduced anxiety (3 trials with 393 women; SMD: −0.28; 95% CI:-0.47,-0.08; low-quality evidence) while listening to music during antepartum hospitalization and during antepartum hospital procedures had no significant effect (very low-quality evidence). Listening to pre-selected music (by researcher) during pregnancy also significantly reduced anxiety (2 trials with 445 women; SMD:-0.83; 95% CI: −1.50, −0.17; very low quality) whereas music listening with patient-preferred music had no effect (very low quality).

Compared to no intervention, music listening during labor (4 trials with 302 women; SMD: −0.96; 95% CI: −1.15, −0.76; low quality) significantly reduced anxiety. For music listening before, during or immediately after cesarean delivery, moderate quality evidence showed no significant effect on pre-operative anxiety (4 trials with 250 women; MD: −3.95; 95% CI: −9.07, 1.18), but significant reductions in intra-operative anxiety (2 trials with 368 women; MD: −0.54; 95% CI: −0.87, −0.2) and post-operative anxiety (7 trials with 847 women; MD: −0.38; 95% CI: −0.53, −0.23). Sub-group analyses demonstrated no significant effect on pre-operative or post-operative anxiety for interventions that employed music listening before and/or during scheduled cesarean deliveries. Yet, listening to music during and/or immediately after scheduled cesarean deliveries significantly reduced anxiety (5 trials with 361 women; MD:-0.49; 95% CI: −0.85, −0.13; moderate quality). Authors of a narrative review also emphasized the benefit of wearing headphones to listen to music during cesarean sections [23].

Reported meta-analyses for music listening in the postpartum period had different comparator groups. Listening to music compared to kangaroo care, psychological treatment, and/or traditional treatment showed no significant effect on anxiety (3 trials with 601 women; SMD: −1.26; 95% CI: −2.81, 0.29, very low quality). Similarly, when limiting comparisons between music listening and only kangaroo care, no effect was found (2 trials with 460 women; RR:0.38; 95% CI: 0.09, 1.63; low quality). Yet, a meta-analysis

of postpartum listening of only five elements music versus no intervention yielded a significant reduction in anxiety rates (2 trials with 524 women; OR: 0.42; 95% CI: 0.19, 0.94; low quality).

#### Effect of music interventions on depression.

Mixed music interventions compared to no intervention significantly reduced depression when implemented in the antepartum period (6 trials with 703 women; SMD:-0.51; 95% CI: −0.80, −0.22) and postpartum period (6 trials with 906 women; SMD: −0.59; 95% CI: −0.96, −0.21). However, this evidence was rated very low quality and when limiting evidence to only trials that measured depression with the Edinburgh Postpartum Depression Scale, mixed music interventions implemented in the antepartum period showed no significant effect (3 trials with 374 women; SMD: −0.44; 95% CI: −0.90, 0.02).

When assessing music listening interventions, various comparisons were reported, all of which reported significant reductions in depression with low to very low-quality evidence. In the antepartum period, listening to five elements music alone or in combination with other treatment significantly decreased depression compared to no intervention (6 trials with 1138 women; SMD:-3.67; 95% CI: −5.21, −2.13; very low quality). In the postpartum period, low quality evidence showed that music listening significantly decreased depression compared to health education, psychological treatment and/or traditional treatment (4 trials with 623 women; RR:0.25, 95% CI: 0.12, 0.54), psychological treatment and Chinese medicine (1 trial with 80 women; RR:0.73; 95% CI: 0.63, 0.86), or no intervention when five elements music is played (2 trials with 240 women; SMD: −0.84; 95% CI: −1.33, −0.35).

#### Effect of music interventions on pain.

Only trials implementing music listening approaches assessed pain intensity. Music listening before or during ART in the preconception period (3 trials with 302 women; MD: −0.96; 95% CI: −1.72, −0.20; low quality), after an episiotomy (7 trials with 677 women; SMD: −1.60; 95% CI: −2.18, −1.02; very low quality) and during labor (4 trials with 332 women; MD: −0.92; 95% CI: −1.33, −0.51; moderate quality) significantly reduced pain compared to no intervention. Sub-analyses investigating the effect of listening to music during labor at different timepoints, with pre-selected music (by researcher), and via headphones all found significant reductions in pain (low to high quality evidence). Only listening to music during labor over hospital speakers showed no significant effect on pain compared to no intervention (4 trials with 200 women; SMD: −0.44; 95% CI: −0.93,0.05; low quality). Hunter et al.’s (2023) narrative review built upon these results with a more nuanced examination of the effect of music listening on pain, suggesting that its analgesic effect waned as labor progressed.

Very low-quality evidence also suggested that listening to music before, during, and/or immediately after cesarean delivery (5 trials with 479 women; MD: −0.82; 95% CI: −1.74, 0.11) and in the postpartum period (3 trials with 318 women; MD: −1.85; 95% CI: −3.96, 0.26) had no effect on pain intensity.

#### Effect of music interventions on other outcomes.

The effect of music listening on stress and sleep was also assessed by various trials/reviews (S11 and S12 Tables). Listening to music during pregnancy did not significantly affect perceived overall stress (2 trials with 532 women; SMD: −0.08; 95% CI: −0.25, 0.09; moderate quality) or pregnancy-specific stress (2 trials with 529 women; SMD: −0.02; 95% CI: −0.19, 0.15; low quality). However, listening to music during pregnancy (4 trials with 348 women; MD:-1.38; 95% CI: −2.56, −0.19; very low quality) and during the postpartum period (1 trial with 82 women; MD: −2.30; 95% CI: −2.75, −1.85; low quality) significantly reduced sleep problems thereby improving sleep quality.

Listening to music during labor had no effect on overall duration or the length of the first stage of labor (S13 Table); however, these analyses were deemed of low quality with only one trial reporting on each of the outcomes. Moderate quality evidence from 2 trials (177 women) found that listening to music during and/or immediately after cesarean deliveries significantly reduced post-operative opioid use (MD: −0.87; 95% CI: −1.55, −0.19). Yet, listening to music during the same time period had no effect on women’s post-operative blood pressure (systolic and diastolic) or heart rate (S14 Table). Low quality evidence also demonstrated that music listening in the postpartum period significantly improved women’s satisfaction (1 trial with 141 women; MD: 2.92; 95% CI: 2.67, 3.17) and maternal attachment (1 trial with 60 women; MD 3.30; 95% CI: 1.72, 4.88; S14 Table).

The effect of mixed music interventions was assessed in the preconception and postpartum periods. In the preconception period, mixed music interventions had no significant effect on vital signs and pregnancy rates (See S13 and S14 Tables). However, moderate quality evidence from 5 trials (554 women) found that mixed music interventions in the postpartum period significantly improved breast milk production (Hedge’s g = 0.39; 95% CI: 0.18, 0.61).

#### Considerations for the heterogeneity of music interventions.

Given the significant heterogeny in music interventions, methods and outcomes across trials, Maul et al. (2024) opted to use descriptive methods instead of meta-analyses to understand the weight of the effect of music interventions employed in the antepartum period and differentiate between key intervention characteristics that may render more beneficial outcomes [20]. Their findings suggest that the type, quantity, and quality of music interventions influence outcomes. Stratified Cohen’s d calculations demonstrated that, contrary to their hypothesis, trials with passive interventions (i.e., music listening) had a large effect on blood pressure among high-risk pregnancies (1 trial of 60 women; Cohen *d*: 1.36) and normal pregnancies (1 trial of 409 women; Cohen *d:*0.81) and on anxiety among healthy women with pre-selected music (1 trail of 409 women; Cohen *d:* 0.96) and self-selected music (1 trial of 80 women; Cohen *d:* 1.27). Stratification by intervention regularity demonstrated that frequent music interventions (≥20 sessions) had a large effect on blood pressure (1 trial with 60 women; Cohen *d*: 1.36), yet small effect on anxiety and depression (1 trial with 36 women; Cohen *d:* 0.40).

Shafqat et al. (2024) builds upon this point emphasizing that the efficacy of music interventions interplays with socio-cultural preferences related to which type and how music interventions are implemented [31]. Therefore, thorough testing of the numerous implementation options for music interventions may not solve issues of heterogeneity alone; the synergistic effects generated when cultural sensitivity is addressed in the design of music interventions for different populations should be considered.

## Discussion

Our overview presents a comprehensive review of the evidence on music interventions employed to improve women’s health outcomes across the perinatal continuum of care. Aggregated findings over the past 15 years suggest that music interventions in the preconception, antepartum, and intrapartum periods have the potential to alleviate anxiety, depression, and pain. Effects from implementation in the postpartum period appear more limited, albeit with a notable exception of demonstrated benefit in reducing depressive symptoms. Our findings are relevant for maternal health care professionals interested in offering music interventions to their patients in various settings (i.e., home or health facility). Also, clinicians can recommend music listening as a safe, low-cost, low-barrier, adjunctive therapy to enhance routine care from preconception through the intrapartum period. Moreover, health care institutions may consider offering music listening during procedures and delivery hospitalization to improve patient experience.

Nonetheless, interpretation of these findings warrant caution due to important methodological limitations with both primary trials and systematic reviews. Heterogeneity in intervention characteristics limits our ability to establish definitive and generalizable recommendations, and to conduct meta-analyses. Considerable RoB plagued most of the cited trials driven by issues with randomization, allocation concealment, and blinding. In addition, only one review [27] assessed the strength of their evidence. Upon our own assessment of study quality, issues with small sample sizes, variability in intervention and outcome measurement, imprecision, and publication bias significantly impacted the level of certainty of the evidence, with very few reviews deemed as having moderate or high quality.

Trials assessing the effect of music listening or passive music interventions were more common in each period, lending little or no insight into how active music interventions and music therapists can be most effectively engaged to improve maternal health outcomes. Subgroup analyses and efforts to assess the magnitude of effect according to key intervention characteristics would be a productive next step in addressing heterogeneity and strengthening the quality of the evidence base; yet findings to date have not synthesized active music interventions alone nor stratified their effect by the conditions or mode of interaction with a music therapist. Notably, no trial or review has assessed the effect of telehealth music therapy modalities on women’s health nor evaluated potential benefits in terms of improved feasibility and accessibility to care during pregnancy and postpartum – this represents a missed opportunity given significant shifts to remote service provision since the COVID-19 pandemic [108,109].

The limited evidence base for several outcomes highlights the need for additional research to enhance the interpretability of findings. For example, while Hoffmann et al. (2025) found that music listening significantly improved sleep quality in pregnancy, the quality of evidence was very low [10]. Similarly, in the postpartum period, Yang et al. (2019) reported significant improvement in women’s sleep, yet based only on one small trial [28]. Additional research is necessary to develop robust recommendations for music interventions that aim to improve maternal sleep quality.

Furthermore, gaps in evidence synthesis were identified for maternal vital signs, particularly regarding blood pressure parameters. Current evidence is low to very-low quality and limited to the preconception and intrapartum periods. Kizilkaya et al. (2024) examined two studies employing music during assisted reproductive technology procedures and found no significant effect [9], while Weingarten et al. (2021) looked at studies utilizing music during cesarean delivery, also finding no significant effect [12]. Although several trials are available [47,48,110,111], no meta-analyses have yet synthesized the effects of antepartum music interventions in women with and/or at-risk for hypertensive disorders of pregnancy (HDPs). Moreover, there is a complete absence of trials evaluating the effect of postpartum music interventions in this population. In the United States, HDPs remain the leading cause of maternal morbidity and mortality [112–116], largely due to challenges in achieving sustained blood pressure control and ensuring adherence to first-line anti-hypertensive therapies [117]. Emerging evidence suggests a shared etiological pathway involving stress that may underlie HDPs and mental health conditions [118]. Given this intersection, further research is needed to determine if music interventions have the potential to address both physiological and psychological outcomes in this high-risk population in ways that respond to women’s preferences for complementary and alternative approaches. Smith et al.’s (2020) systematic review expands this inquiry by examining the effect of the spectrum of mind body interventions (i.e., relaxation, yoga, guided imagery, and music) for women with HDPs, yet comes to similar conclusions – that further research is warranted to guide the use of music interventions with pregnant and postpartum women [119].

Our overview of reviews has several limitations. Due to the overall poor quality of the primary trials, we elected not to reanalyze or conduct our own meta-analyses. As such, our synthesis and interpretation of the evidence has been limited to prior assessments fraught with significant heterogeneity and imprecision. To mitigate some of this issue, we elected to not present estimated effects of grouped trials that implemented music interventions across various perinatal periods and, in turn, emphasized subgroup findings that aimed to improve the comparability of similarly designed music interventions. Since there have been no Cochrane reviews focusing on music interventions for our population of interest, we also encountered significant overlap of reported trials across several systematic reviews. When possible, we excluded reviews with overlapping primary studies if more recent and higher quality reviews were available [17] and presented the most recent meta-analyses conducted for relevant comparisons. During the review process, we discovered that a trial cited in several reviews [23,24,30] had been retracted due to “systematic manipulation of the publication and peer-review process [120].” As a result, we excluded this trial and all associated meta-analyses to maintain the integrity of our findings.

## Conclusions

Based on the current evidence base, music interventions are a promising approach for women across the perinatal continuum of care. However, results should be interpreted with caution. Significant concerns with the methodological rigor need to be addressed before recommendations for using music interventions in pregnant and postpartum women are developed. Further research is needed to identify design characteristics and implementation modalities of music interventions that most effectively improve women’s health outcomes. Strengthening the evidence on music interventions is vital to informing the effective integration of CAM approaches into person-centered care strategies for women’s health.

## Supporting information

S1 Table Literature search strategy. (PDF)

S2 Table Characteristics of excluded literature. (PDF)

S3 Table Primary study mapping. (PDF)

S4 Table Registered literature reviews. (PDF)

S5 Table Characteristics of primary studies included in each review. (PDF)

S6 Table Methodological quality of systematic reviews using the AMSTAR 2 tool (N = 20). (PDF)

S7 Table Primary study risk of bias assessments (RoB-1). (PDF)

S8 Table Summary of effects of music interventions on anxiety. (PDF)

S9 Table Summary of effects of music interventions on depression. (PDF)

S10 Table Summary of effects of music interventions on pain. (PDF)

S11 Table Summary of effects of music interventions on stress. (PDF)

S12 Table Summary of effects of music interventions on sleep. (PDF)

S13 Table Summary of effects of music interventions on other outcomes. (PDF)

S14 Table Summary of effects of music interventions on vital signs. (PDF)

S1 Appendix PRIOR checklist for review of reviews. (PDF)
