# Supplementary material for: Music interventions to improve women’s health outcomes in the preconception, antepartum, intrapartum, and postpartum periods: An overview of reviews
Source: PLoS One. 2026 Feb 18;21(2):e0339337. doi: 10.1371/journal.pone.0339337 (PMC12915951; doi:10.1371/journal.pone.0339337)
Supplement: S1 Table — (PDF) [file pone.0339337.s001.pdf]

Supplementary Materials

Table S1: Literature Search Strategy

PUBMED

Population	("pregnancy"(MeSH Terms) OR "pregnan*"(Title/Abstract) OR "antepartum"(Title/Abstract) OR "antenatal"(Title/Abstract) OR "prenatal"(Title/Abstract) OR "postpartum"(Title/Abstract) OR “post partum”(Title/Abstract) OR "Postpartum Period"(Mesh) OR puerperium(Title/Abstract) OR "matern*"(Title/Abstract) OR "perinatal care"(MeSH Terms) OR "perinatal"(Title/Abstract) OR "Delivery, Obstetric"(Mesh) OR delivery(Title/Abstract) OR obstetric*(Title/Abstract) OR "Obstetrics"(Mesh) OR childbirth(Title/Abstract) OR intrapartum(Title/Abstract) OR abortion(Title/Abstract) OR "Abortion, Induced"(Mesh) OR "Abortion, Therapeutic"(Mesh) OR "Abortion, Missed"(Mesh) OR "Abortion, Incomplete"(Mesh) OR breastfeeding(Title/Abstract) OR “breast feeding”(Title/Abstract(OR "Breast Feeding"(Mesh))
Intervention	("music"(MeSH) OR "Music Therapy"(MeSH Terms) OR music(Title/Abstract))
Filters	(meta-analysis(Filter) OR review(Filter) OR systematicreview(Filter)) AND AND (humans(Filter)) AND (2010/1/1:2025/3/10(pdat)))

EMBASE

Population	('pregnancy'/exp OR 'pregnan*':ab,ti OR 'puerperium'/exp OR ‘puerperium’:ab,ti OR 'postpartum':ab,ti OR 'post partum':ab,ti OR 'maternal care'/exp OR 'matern*':ab,ti OR 'perinatal care'/exp OR 'perinatal':ab,ti OR 'obstetrics'/exp OR 'obstetric*':ab,ti OR 'childbirth'/exp OR 'childbirth':ab,ti OR 'intrapartum':ab,ti OR 'abortion':ab,ti OR 'abortion'/exp OR 'breast feeding'/exp OR 'breast feeding':ab,ti)
Intervention	('music therapy':ab,ti OR 'music':ab,ti)
Filters	'human'/de AND ('review':ab,ti OR 'meta analysis':ab,ti) AND (2010:py OR 2011:py OR 2012:py OR 2013:py OR 2014:py OR 2015:py OR 2016:py OR 2017:py OR 2018:py OR 2019:py OR 2020:py OR 2021:py OR 2022:py OR 2023:py OR 2024:py OR 2025:py)

SCOPUS

Population	((TITLE-ABS-KEY(pregnan*) OR TITLE-ABS-KEY(antepartum) OR TITLE-ABS-KEY(antenatal) OR TITLE-ABS-KEY(prenatal) OR TITLE-ABS-KEY(postpartum) OR TITLE-ABS-KEY(“post partum”) OR TITLE-ABS-KEY(puerperium) OR TITLE-ABS-KEY(matern*) OR TITLE-ABS-KEY(perinatal) OR TITLE-ABS-KEY(delivery) OR TITLE-ABS-KEY(obstetric*) OR TITLE-ABS-KEY(childbirth) OR TITLE-ABS-KEY(intrapartum) OR TITLE-ABS-KEY(abortion*) OR TITLE-ABS-KEY(breastfeeding) OR TITLE-ABS-KEY(“breast feeding”))
Intervention	TITLE-ABS-KEY(music)
Filters	(TITLE-ABS-KEY(“meta-analysis”) OR TITLE-ABS-KEY(review)) AND (PUBYEAR > 2009 AND PUBYEAR < 2026))

PsychINFO

Population	(TI (pregnan* OR antepartum OR antenatal OR prenatal OR postpartum OR “post partum” OR matern* OR perinatal OR delivery OR obstetric* OR childbirth OR intrapartum OR abortion* OR breastfeeding OR “breast feeding”) OR AB (pregnan* OR antepartum OR antenatal OR prenatal OR postpartum OR “post partum” OR matern* OR perinatal OR delivery OR obstetric* OR childbirth OR intrapartum OR abortion* OR breastfeeding OR “breast feeding”))
Intervention	(TI music OR AB music)
Filters	(TI (“meta-analysis” OR review) OR AB (“meta-analysis” OR review)) AND Publication Date: 20100101-20251231
