# Supplementary material for: Music interventions to improve women’s health outcomes in the preconception, antepartum, intrapartum, and postpartum periods: An overview of reviews
Source: PLoS One. 2026 Feb 18;21(2):e0339337. doi: 10.1371/journal.pone.0339337 (PMC12915951; doi:10.1371/journal.pone.0339337)
Supplement: S2 Table — (PDF) [file pone.0339337.s002.pdf]

Supplementary Materials

Table S2: Characteristics of Excluded Literature

Review	Reason for exclusion	Date of search	Objective	Study Design	Types of studies included	Subjects	Interventions	Comparators	Outcomes	No. of studies
Adjie 2025	Music not primary focus	Aug/ Sep 2023	To assess the effect of supportive interventions pre- and during delivery on the likelihood of a positive childbirth experience.	Systematic review	RCTs	Low-risk pregnant and childbearing women who have not been diagnosed with any issues	Any approach to improving childbirth experience during pregnancy or labor	Regular treatment provided by medical professionals following the hospital's and maternity care unit's clinical guidelines	Women's assessment of their own labor and delivery experiences	15
Hasanul Huda 2025	Wrong patient population	Aug 2023	To investigate the relative effectiveness of non-pharmacological interventions involving parents in alleviating discomfort in neonates	Network meta-analysis	RCTs	Infants who have completed at least 37 weeks of gestation and had invasive procedures in the Neonatal Intensive Care Unit	Non-pharmacological interventions involving parents	Standard of care, placebo, and other therapies targeting pain reduction	Pain	35
Rahmawati 2025	Music not primary focus	NR	To evaluates non-pharmacological interventions targeting the prevention and management of postpartum blues among pregnant and postpartum women.	Systematic review	RCTs and quasi-experimental	Primiparous, nulliparous, and multiparous with age ≥ 15 years in the postpartum period (≤ 2 years postpartum)	Non-pharmacological interventions	Placebo or treatment as usual	Postpartum blues	17
Zeng 2025	Music not primary focus	Oct 2024	To explore and compare the impact of various non-pharmacological interventions in improving depressive and anxiety symptoms, and to identify the most effective strategies for	Systematic review and meta-analysis	RCTs or quasi-RCTs	Pregnant women exhibiting early signs of depressive and/or anxiety symptoms during pregnancy but did not meet clinical	Mindfulness, education, counseling, cognitive behavioral therapy, muscle acupoint therapy, relaxation, mind-body exercise, psychotherapy, foetal movement counting, physical exercise, and music.	Usual care	Depression and anxiety	101

			pregnant women with depressive and/or anxiety symptoms.			diagnostic criteria or exceed the threshold for clinically significant symptoms				
Abera 2024	Wrong study type	Aug 2023	To systematically synthesize evidence on the effects of relaxation interventions on maternal stress and mental health during pregnancy and on pregnancy and birth outcomes.	Systematic review and meta-analysis	RCTs or quasi-experimental designs	Apparently healthy pregnant women	Relaxation interventions that were applied during pregnancy with the aim of reducing stress and promoting mental health	Pregnant women who did not receive a stress-reduction relaxation intervention but who received treatment as usual.	Stress, mental health problems, pregnancy outcomes, birth outcomes and maternal physiology	32
Cavero 2024	Wrong study type	Jan 2023	To systematically examine how musical interactions between infants and their caregivers have been studied in relatively natural situations	Systematic review	Empirical observational studies	Infants under 36 months and their parent or primary caregiver	Musical interactions in which both the infant and caregiver engaged together in at least one of the following activities: singing, dancing, playing musical instruments, listening to music, or creating rhythms	NR	Procedures employed for intervention, adult, infant, and dyadic behaviors	27
Feng 2024	Music not primary focus	Jan 2023	To provide a comprehensive summary of the available evidence on the impact of different strategies for reducing labor pain	Systematic review and network meta-analysis	RCTs and CCTs	Women in labor	Nonpharmacological coping strategies for reducing labor pain	Control group	Labor pain	9
Kaplan 2024	Wrong study type	March 2023	To investigate the effectiveness of nonpharmacological methods used to reduce the severity of RLS and related symptoms during pregnancy, based on	Systematic review and meta-analysis	RCTs and semi-experimental	Pregnant women with restless leg syndrome	Non-pharmacological methods (water temperature, heat therapy, leg stretches, music, exercises, sleep hygiene)	Control group or placebo group	Presence and severity of RLS, physical problems such as sleeping and hurting	6

			the results of previous studies							
Levene 2024	Music not primary focus	Sep 2023	To appraise all available evidence on whether the provision of relaxation interventions to lactating individuals improves lactation and well-being	Systematic review and meta-analysis	RCTs	NR	M=Music, guided relaxation, mindfulness, and breathing exercises/muscle relaxation	Control group	Human milk quantity, length and exclusivity of human milk feeding, milk macronutrients/cortisol, and infant growth and behavior.	16
Marin 2024	Music not primary focus	NR	To analyze the obstetric effects of relaxation techniques on pain management during labour	Systematic review	RCTs, systematic review and/or meta-analyses, observational studies, and clinical practice guidelines	Women in labour	non-pharmacological analgesic methods used to control pain in labour,	NR	Pain during labour	11
Park 2024	Wrong patient population	Jan 2023	To examine the effectiveness of technology-based music interventions for the anxiety and pain of hospitalized patients undergoing procedures	Systematic review	RCTs	Hospitalized patients undergoing procedures	Music interventions using interactive technology (e.g., smartphones, mHealth, tablets, applications, and virtual reality)	NR	Pain and anxiety	21
Peng 2024	Wrong study design	NR	To investigate the association between pregnancy-related complications and post-traumatic stress disorder (PTSD) among postpartum women	Scoping review	RCTs, before-after studies, prospective cohort studies, cross-sectional studies, and case reports	Women with PTSD caused by pregnancy-related complications	One or more psychological interventions to prevent and/or treat PTSD resulting from pregnancy-related complications, including music therapy	NR	NR	21
Xu 2024	Music not primary focus	May 2024	To ascertain the effectiveness of creative art therapy (CAT) in alleviating symptoms of	Systematic review and meta-analysis	RCTs	Women diagnosed with PPD	CAT intervention in PPD including music (listening and singing), painting, dance, sculpture, film, poetry, drama,	Standard nursing, including a series of medical, nursing, developmental	Depression, anxiety, mother-infant relationship, satisfaction with intervention, and personal state	12

			postpartum depression (PPD)				psychological drama, and situational drama	and supportive nursing.		
Abd-El Gawad 2023	Wrong patient population	Sept 2022	To summarize the current evidence-based knowledge on the potential effect of music as a pain and anxiety reliever for patients undergoing outpatient hysteroscopy	Systematic review and meta-analysis	RCTs	Women who underwent outpatient hysteroscopy	Music	No music	Pain and anxiety	3
Cabral 2023	Music not primary focus	Sept 2020	To evaluate the effectiveness of non-pharmacological measures used by obstetric nurses to relieve pain during labor	Systematic review	RCTs	Obstetric patients	Non-pharmacological measures	Standard nursing care, own group, placebo, and no intervention	Pain relief	17
Dube 2023	Music not primary focus	Jan 2022	To provide an updated estimate of the overall effect of psychological interventions on psychological outcomes and pregnancy rates among infertile couples and/or individuals with infertility as the available evidence is thus far conflicting	Systematic review and meta-analysis	RCTs	Women either receiving medical treatment for infertility or meet the definition of infertility	Psychological interventions: non-pharmacological protocol based in psychological theory and aimed at bringing about change in at least one aspect of psychosocial wellbeing	Active or inactive control group	Depressive symptoms, anxiety, psychological well-being, infertility-related distress, marital satisfaction or pregnancy	58
Gutiérrez-Ortega 2023	Wrong patient population	NR	To synthesize music therapy's impact on different domains of preterm infant development when used in the Neonatal Intensive Care Unit (NICU) and led by music therapists	Systematic review	NR	Infants born before 37 weeks of gestation, or regardless of gestational age, those who had stayed in the NICU	Music therapy interventions performed by certified music therapists, explicitly stated, including live music, recorded music, and creative music therapy	NR	Infant vital signs, feeding, quality of sleep, brain oxygenation, well-being and maternal anxiety	21

Janssen 2023	Music not primary focus	April 2020	To assess whether stress-reducing interventions can reduce the incidence of PTB, specifically in a low-risk obstetric population	Systematic review and meta-analysis	RCTs, CCTs, cohort studies, and observational studies	Pregnant women	Stress-reducing interventions	NR	Preterm birth, low birth weight, mean gestational age at time of birth, mean birth weight, maternal anxiety, maternal stress, and adverse perinatal outcomes	10
Konsam 2023	Overlap with other studies	August 2022	To assess if listening to music reduces perinatal anxiety among pregnant women	Systematic review	RCTs and CCTs	Pregnant women and their newborns	Music listening	No intervention or routine care	Anxiety	10
Le Lous 2023	Wrong study design		To systematically examine studies published on the physical enviro--ent in the obstetrical operating room during c-sections and its impact on mother and neonate outcomes.	Systematic review	Clinical trials, randomized clinical trials, meta-analysis, reviews, and systematic reviews	NR	Investigations of the physical operating room enviro--ent during cesarean sections	NR	NR	8
Martinez-Shaw 2023	Wrong study design	March 2022	To describe and classify the different interventions for parents to reduce the stress experienced by the arrival of a premature infant and to have an overview of their effectiveness.	Systematic review	RCTs, quasi-experimental, longitudinal studies	Parents of premature infants	Psychoeducational, parental support programs, relaxation techniques, expressive writing, art therapy, music therapy, tactile interaction and stimulation	Active comparator or standard care	Parental stress, PTSD	46
Nori 2023	Wrong study design	July 2023	To investigate up-to-date non-pharmacological pain management (NPPM) understanding and application, specifically focusing on women’s individual needs, regarding NPPM’s effectiveness,	Systematic review	NR	Pregnant >18 years old during childbirth	Interventions that reduced labor pain and shortened labor duration	NR	Labor pain and labor duration	94

			advantages, limitations, and potential adverse effects							
Pino 2023	Wrong study design	NR	To describe the potentialities of music exposure on fetus, and on preterm newborns in the Neonatal Intensive Care Unit evaluating its influence on neurobehavioral development	Systematic review	Cohort, cross-sectional, prospective, RCTs, controlled clinical trials	Pregnant women, fetuses, premature newborns, term newborns, children	Music	NR	NR	28
Qian 2023	Music not primary focus	March 2022	To assess the efficacy of art-based interventions when delivered to pregnant and postpartum women	Meta-analysis	RCTs	Healthy pregnant or postpartum women (within the 1st year following childbirth) with no perinatal complications or mental health diagnoses	Art-based interventions implemented during pregnancy	Usual care, waitlist, or no intervention	Anxiety, distress, grief, depression, stress or posttraumatic stress disorder measured by self-reported psychological inventories	21
Beyable 2022	Wrong study design	NR	To standardize and increase the quality of care in labor pain management by using available and cost-effective resources with minimal complications.	Systematic review	Original articles, Meta analysis, systematic review, randomized control trial, comparative and cross sectional studies	NR	Pharmacologic and non pharmacologic labor pain management	NR	NR	40
Lorenzo 2022	Wrong study design (Conference Abstract)	NR	To evaluate the effectiveness of triadic music therapy in reducing parents' and their preterm infants' distress levels	Systematic Review	RCTs, non-randomized clinical trials	Parents and pre-term infants	Creative music therapy, premature triadic music therapy, family-centered music therapy, rhythm, breath, and lullaby program, and generic	Control Groups were standard care and skin-to-skin contact	Infant distress and vital signs	6

							improvisational music therapy			
Melillo 2022	Music not primary focus	May 2021	To conduct a meta-analysis to evaluate the efficacy of non-invasive and nonpharmacological techniques on labor first-stage pain intensity	Meta-analysis	RCTs	Women in first stage of labor	Non-pharmacological, non-invasive, or minimally invasive intrapartum analgesic techniques alternative and/or complementary to pharmacological analgesia	Routine intrapartum care or placebos	Subjective pain intensity	63
Onyekere 2022	Music not primary focus	NR	Systematic review aimed at investigating the minimal-contact physical interventions for pregnant women with musculoskeletal disorders	Systematic Review	RCTs and non-RCTs	Pregnant women with musculoskeletal conditions	Minimal contact interventions	Usual care, no intervention, placebo or control groups	Pain intensity, disability and quality of life	10
Paulino 2022	Music not primary focus	NR	To investigate the effect of non-pharmacological interventions to improve sleep quality during pregnancy	Systematic review and meta-analysis	Prospective clinical trials	Pregnant women	Non-pharmacological interventions (music, physical exercise, relaxation exercises, lettuce seed, sleep hygiene or acupressure)	Control group without intervention or with the usual care	Sleep improvement	8
Dominguez-Solis 2021	Wrong study Design	Dec 2018	To determine which non-pharmacological interventions are known to be effective in reducing anxiety during pregnancy, childbirth, and postpartum.	Systematic Review	RCTs or quasi-experimental designs	Low-risk pregnant women, healthy women with healthy newborns, women with full-term newborns	Non-pharmacological interventions	Control groups following treatment as usual	Anxiety	21
Dukic 2021	Wrong study design	NR	To review of current research on the influence of music on resilience in pregnancy and its	Conference abstract of literature review	NR	NR	Music	NR	Production of dopamine, oxytocin and growth hormone	NR

			consequential effect on the production of dopamine, oxytocin and growth hormone in pregnant women							
Eidelman 2021	Wrong study design	NR	NR	Editorial	NR	NR	Music	NR	Breastfeeding rates	NR
He 2021	Wrong Outcomes	March 2021	To investigate the effects of prenatal music therapy on fetal and neonatal status.	Systematic review and meta-analysis	RCTs	Pregnant women	Music	No music therapy	Fetal heart rate, number of fetal movements, and number of acceleration, neonatal heart rate, respiratory rate, Apgar score, and feeding status	9
Katyal 2021	Music not primary focus	Feb 2020	To investigate the effect of psychosocial interventions on pregnancy rates in women or couples undergoing Assisted Reproduction Technologies (ART) treatment compared to pregnancy rates in women or couples undergoing ART treatment and not receiving psychosocial interventions	Systemaitc review and meta-analysis	RCTs	Women/couples with different types of infertility and different types and stages of ART treatment	Interventions with a psychosocial aim that did not include medication or had any physiological focus including acupunctire, musc, writing, CBT, and mind-body interventions in either an individual, couple or group setting		Pregnancy rate including biochemical pregnancy rate and clinical pregnancy rate, and live birth rate, and abortion rate	15
Mahmoud 2021	Primary study overlap	May 2021	To evaluate the effect of music therapy on anxiety and pregnancy rates among infertile women undergoing to perform assisted reproductive technologies	Systematic review and meta-analysis	RCTs	Infertile women undergoing assisted reproductive technology procedures	Music listening and music therapy	No intervention	Anxiety, Pregnancy rate, Satisfaction	7
Shimada 2021	Wrong study design	June 2019	To investigate in the literature the studies on the benefits of music therapy	Systematic review	Any	Pregnant women	Music or music therapy	NR	Relaxation, anxiety, psychosocial stress and depression, pain, maternal bond, quality	23

			interventions among pregnant women in the prenatal, delivery and postpartum periods.						of sleep, fetal heart rate, maternal blood pressure, and intake of drugs in the postoperative period	
Widiasih 2021	Music not primary focus	NR	To identify various health interventions to prevent PPD in Indonesia.	Systematic review	Quasi-experimental or pre-post-test design	Women in postpartum period or third trimester of pregnancy	Techniques to prevent PPD including counseling, music, and aromatherapy	Control group or within group	PPD	10
Zhu 2021	Music not primary focus	April 2021	To retrieve the latest and best evidence about music, massage, yoga and exercise in the prevention and treatment of prenatal depression, and to preliminarily compare the four methods to explore the most effective means.	Systematic review and meta-analysis	RCTs	Pregnant women with suspected or confirmed depression	Music, exercise, massage, and yoga, performed during pregnancy	Waiting-list control, usual care, or active controls	Antenatal depression and anxiety	24
Santiváñez-Acosta 2020	Primary study overlap	June 2018	To evaluate the effectiveness of music therapy to manage pain and anxiety during labor	Systematic review and meta-analysis	RCTs and CCTs	Pregnant women during labor	Music listening	No intervention	Anxiety, depression, pain, vital signs	12
Smith 2020	Music not primary focus	Jan 2019	To undertake a systematic review of the safety and effectiveness of mind body approaches for women with hypertensive disorders in pregnancy	Systematic review and meta-analysis	RCT and quasi-RCT	Pregnant women with hypertension, preeclampsia, or risk of hypertension	Yoga, classical music, guided imagery, biofeedback, Benson relaxation, acupunctures, relaxation techniques	Standard care, bed rest, stretching exercises, oral hypertensives	Blood pressure reduction, preeclampsia, eclampsia, morbidities, medication use, pre-term birth, antenatal hospital admission and duration of stay, side effects, small for gestational age, NICU admission, and Apgar.	8
Wang 2020	Music not primary focus	Feb 2020	To determine the efficacy of nonpharmacological interventions in terms of women's satisfaction with	Systematic review	RCTs, case-control studies, cohort studies, quasi-experimental studies	Women seeking abortion	Skill-based health education, support person in recovery room, music, text messages, doula support, visit and	No intervention, other intervention, pre-post comparison	Satisfaction with care, psychological well being	10

			care and psychological outcomes during and subsequent to abortion.				waiting period requirements, counseling, smartphone applications			
Yue 2020	Wrong patient population	Nov. 2029	To examine the effectiveness of music therapy on pre-term infants in neonatal intensive care unit	Systematic review and meta analysis	RCT	Preterm infants born before 37 wks gestation	Music therapy conducted by professional therapists or caregivers in NICU	Any	Any	13
Zimpel 2020	Music not primary focus	Sept 2019	To assess the effects of CAM for post-caesarean pain	Systematic review and meta analysis	RCTs, cluster RCTs, and quasi-randomized trials	Women in the postpartum period after a CS.	All types of CAM according to WHO criteria including acupuncture or acupressure, aromatherapy, massage, music therapy, Reiki, relaxation and transcutaneous electrical nerve stimulation	Placebo, no treatment, placebo plus analgesia, analgesia alone	Pain, adverse effects, vital signs, rescue analgesic requirement, pain after six weeks from discharge, patient satisfaction, breastfeeding status, interaction with baby, walking at discharge, length of hospitalization	32
Mascarenhas 2019	Wrong study design	NR	To identify studies on the efficacy of non-pharmacological methods in reducing labor pain in the national and international literature	Integrative review	RCTs, quasi-experimental, and qualitative studies	Parturient	Non-pharmacological methods of relief including acupuncture and its main variations, hydrotherapy, Swiss ball exercises, music, and breathing exercises	Control groups or placebo groups when available	Labor pain	19
Dombrowska-Pali 2018	Wrong study design	NR	NR	Literature review	NR	Women in antepartum, intrapartum, and postpartum periods, fetuses, and babies	Music therapy	NR	NR	NR
Moayedi 2018	Wrong study design	Jan 2018	To review the literature regarding pharmacologic and non-pharmacologic pain control options	Literature Review	NR	Women who have undergone a first trimester aspiration abortion	Pain management regimens	NR	Pain during first-trimester uterine aspiration abortion	NR

			for first-trimester abortion performed in the clinical setting.							
Smith 2018	Music not primary focus	June/ Aug 2017	To assess the effect, safety and acceptability of massage, reflexology and other manual methods to manage pain in labour	Systematic review and meta-analysis	RCTs, cluster RCTs, and quasi-RCTs	Women in labour	Massage, warm packs, thermal manual methods, reflexology, chiropractic, osteopathy, musculo-skeletal manipulation, deep tissue massage, neuro-muscular therapy, shiatsu, tuina, trigger point therapy, myotherapy and zero balancing	Placebo/no treatment, hypnosis, biofeedback, intracutaneous or subcutaneous sterile water injection, immersion in water, aromatherapy, relaxation techniques, acupuncture or acupressure.	Pain intensity, satisfaction with pain relief, sense of control in labour, satisfaction with childbirth experience	10
Smith 2018	Music not primary focus	May 2017	To examine the effects of mind-body relaxation techniques for pain management in labour on maternal and neonatal well-being during and after labour	Meta-analysis	RCTs, cluster-RCTs, quasi-RCTs	Women in labour	Relaxation methods, yoga, music, audio analgesia and mindfulness	Placebo/no treatment, hypnosis, biofeedback, intracutaneous or subcutaneous sterile water injection, immersion in water, aromatherapy	Pain intensity, satisfaction with pain relief, sense of control in labour, satisfaction with childbirth experience	15
Becker 2016	Music not primary focus	March 2016	To assess acceptability, effectiveness, safety, effect on milk composition, contamination and costs of methods of milk expression	Systematic review and meta-analysis	RCTs and quasi-randomized trials	Women expressing or pumping milk for any reason by any method, who may or may not also be feeding a child at the breast	Provided instructions or support protocols on hand expression or mechanical pumping, provided hand expression or mechanical pumping equipment, or if the study required expression or pumping using a specific protocol or adjunct behavior	NR	Maternal satisfaction, indicators of possible adverse outcomes for mother or infant, transfer to feeding at breast, quantity of milk expressed, nutrient quality of expressed milk, maternal physiological effects of expressing, economic costs	41

BieleninikŁ 2016	Wrong patient population	April 2015	To systematically review and meta-analyze the effect of music therapy on preterm infants and their parents during NICU hospitalization and after discharge from the hospital	Systematic review and	RCTs	Children born Prematurely (<37 completed weeks of gestation)	All forms of music therapy carried out by, or in consultation with, a trained music therapist	Standard care alone, standard care combined with other therapies, or standard care with placebo	Psycho- developmental, behavioral, physiologic, anthropometric, socioemotional development, parental functioning, adverse effects, and length of hospitalization	16
Tschann 2016	Wrong study design	NR	To identify areas of consensus in the literature regarding nonpharmacologic pain control adjuncts during first-trimester aspiration abortion.	Systematic review	Randomized and non-randomized studies	Women undergoing first-trimester aspiration abortions	Non-pharmacologic pain management interventions	Control groups	Pain and anxiety	7
Fleury 2014	Wrong study design	Jan 2014	To review the literature, listing the benefits of music therapy with patients in other correspondent themes.	Literature review	NR	Women undergoing assisted reproduction	Music	NR	Stress	22
Lieberman 2014	Music not primary focus	NR	To conduct a systematic review of the current preventive and treatment interventions of perinatal depression specifically tested for adolescents, with a focus on low SES, racial or ethnic minority populations.	Systematic review	RCT, and Pre-Post intervention studies	Pregnant adolescents or adolescent mothers	Preventive and treatment interventions of perinatal depression	Any	Depression	10
Renner 2010	Music not primary focus	NR	To compare the effect of different methods of pharmacological and nonpharmacological pain control administered prior	Systematic Review	RCTs	Pregnant women prior to or during a first-trimester surgical abortion at less than 14 weeks	Pharmacological pain control administered via mucosal, intramuscular, or intravenous routes or	Control groups or placebo groups	Pain control or perceived pain, adverse effects, side effects, and patient satisfaction	40

			to or during first-trimester surgical abortion (b14 weeks gestation with electric or manual suction aspiration) on patient perceived pain, satisfaction, side effects and safety.			gestational age using electric or manual suction aspiration	nonpharmacological pain control			
Schmid 2010	Wrong study design; wrong patient population	2010	To assess whether and how home-based music therapy can be part of an innovative and effective service for people who have to be treated at home	Literature review	Observational studies, RCTs, case studies, qualitative studies	Anyone receiving care at home	Home-based music therapy	NR	Any	20
NR: NR										

				Intervention 2 (19): Relaxation intervention: 1 session (60 minutes)<br>Control (42): Waitlist attention group				
Browning 2001		RCT	Primiparous women with planned hospital vaginal birth	Intervention (10): Session with a music therapist to select soothing anxiety-relieving and rhythmic music followed by music listening during labor: six 90-min tapes of recorded music provided to use on cassette player or Walkman<br>Control (10): Routine care	Relaxation and control as proxies for anxiety<br>Pain	Labor agentry scale - attitude towards childbirth scale; Trippet objective muscle relaxation inventory McGill pain scale;	NR	Hunter 2023
Buglione 2020	Italy	RCT	Nulliparous women at full-term with singleton pregnancy having a vaginal birth	Intervention (15): Music listening via speakers throughout their labor: participant choice of gentle popular music, soft classical music, and Israeli tunes<br>Control (15): Routine care	Anxiety<br>Pain	VAS-A<br>VAS-P	Vaginal Delivery, episiotomy pain at 1, 24, 48 h	Hunter 2023; Ji 2024; Shafqat 2024, Maleki 2023, Sen 2023
Cao 2016	China	RCT	Pregnant women admitted to hospital with pregnancy-induced hypertension	Intervention (30): Music listening using a MP3 player with headphones for 30-60mins a day for 4 weeks; participant's preferred music or selection from recorded CD<br>Control (30): Routine care	Anxiety<br>Vital signs<br>Depression	HAM-A<br>Systolic and diastolic blood pressure<br>HAM-D	Baseline and post-intervention	Lin 2019; Maul 2024; Shafqat 2024; Sun 2024
Cappon 2014		CCT	Anxious pregnant women	Intervention (36): music listening of pre-recorded psychoacoustically modified music preselected by researcher: 20 sessions (15-30 min)<br>Control (37): Not specified	Anxiety	BAI	NR	Dogan-Gangal 2024
Catalgol 2021	Turkey	RCT	Pregnant women 36-38 wks gestation	Intervention (50): Music listening during non-stress test with a MP4 player; participant selection of 12 classical Turkish songs<br>Control (50): Not specified	Anxiety	STAI-S, STAI-T	Before and after non-stress test, before NST at 36 and 38w	Ji 2024; Maul 2024; Shafqat 2024
Chaichanalap 2018	Thailand	RCT	Primiparous and multiparous women who had vaginal delivery	Intervention (50): "A Yellow Brick Cinema-Relaxing Piano Music" through earphones from episiotomy repair to 6h postpartum<br>Control (50): Routine care	Pain	VAS-P	Vaginal delivery, 2h and 6h post-repair	Maleki 2023
Chang 2008	Taiwan	RCT	Pregnant women: 18-22 or 30-34 wks gestation, medically low risk	Intervention (116): Routine ANC and listening to music for 2 weeks for 30minutes/day; participant selection among 4 types of music: lullabies,	Anxiety<br>Depression<br>Stress	STAI<br>EPDS<br>PSS	Baseline, immediately post intervention	Corbijn van Willenswaard 2017; Dogan-Gangal 2024; Han

				classical music, nature sounds, and crystal music with 60-80 beats/min				2024; Lin 2019; Maul 2024; Shafqat 2024; Sun 2024
				Control (120): Routine ANC				
Chang 2015	Taiwan	RCT	Pregnant women: ≥17 wks gestation, medically low risk	Intervention (145): Music listening using a CD, headphones or speakers at home; daily sessions (30mins) for 2 weeks; participant selection among 4 types of music: lullabies, classical music, nature sounds, crystal and symphonic music with 60-80 beats/min	Stress	PSS, pregnancy stress rating scale	Baseline and immediately post intervention	Corbijn van Willenswaard 2017; Dogan-Gangal 2024; Maul 2024
				Control (151): Routine ANC				
Chang and Chen 2005	Taiwan	RCT	Pregnant women scheduled for a cesarean section	Intervention (32): Music listening using a portable C play with headphones from beginning of anesthesia administration to end of cesarean section; participant choice of anxiety-relieving music including western classical, new-age, or Chinese religious songs	Anxiety	VAS-A	NR	Hunter 2023; Shafqat 2024; Weingarten 2021
				Control (32): Routine care				
Cheung 2018	China	RCT	Women undergoing fertility treatment	Intervention (66): Calming non-lyrical music through headphones during oocyte collection, approximately 60-80bpm, played at 60dB: once for 30m	Anxiety<br>Pain<br>Depression<br>Patient satisfaction	STAI<br>VAS<br>BDI<br>Client Satisfaction Questionnaire	NR	Kizilkaya 2024
				Control (65): Headset without music				
Choubsaz 2018	Iran	RCT	Pregnant women having a planned cesarean section	Intervention (30): Music listening during the entire cesarean section; pre-selected music by the researchers of sedative musical piece of Iranian Music Therapy Association	Anxiety	STAI	NR	Weingarten 2021
				Control (60): Ear plugs or routine care				
Dabas 2019	India	RCT	Mothers with newborns	Intervention (29): Passive listening to an audio-assisted relaxation technique on a laptop: once daily for 10 days (10 total sessions) for 30m	Breastmilk volume	ml	NR	Duzgun 2020
				Control (28): Standard nursing care				
Dehcheshmeh and Rafiei 2015	Iran	RCT	Primiparous pregnant women at full term having a vaginal birth	Intervention 1 (37): Music listening using headphones for 30 minutes: participant choice of piano music or wave sounds	Anxiety	VAS-A	NR	Hunter 2023; Sen 2023
				Intervention 2 (37): Ice massage				
				Control (38): Routine care				

Denney 2018	USA	RCT	Pregnant women scheduled for a cesarean section	Intervention (25): Music listening using a MP3 player while in the preoperative waiting area and postoperative recovery room; participant choice of a playlist with classical, pop/top 40, R&B, country, soft rock, or gospel music	Anxiety	STAI	NR	Hunter 2023; Weingarten 2021
				Control (25): Routine care				
Dereddy 2024	USA	RCT	Women in postpartum period	Intervention (20): Music therapy in the NICU with a music therapist: 4 times a day for 7 days (30mins): participant selection of preferred music	Anxiety<br>Depression<br>Stress	DASS-21	NR	Han 2024
				Control (20): Routine care				
Dolker 2019	Turkey	RCT	Pregnant women 32-41 wks gestation	Intervention (50): Music listening during non-stress test; selection of Turkish classical, folk, or classical music	Anxiety	STAI	NR	Ji 2024
				Control (50): Not specified				
Drzymalski 2023	US	RCT	Nulliparous women having an elective cesarean section	Intervention (10): Music listening via broadcast immediately before patient entry and throughout entire cesarean section; pre-selected Mozart sonatas	Anxiety	Maternal satisfaction scale for cesarean sections	NR	Shafqat 2024
				Control (10): Routine care				
Drzymalski 2020	US	RCT	Pregnant women having and elective cesarean section	Intervention 1 (49): Music listening before, during and after cesarean section; participant choice of preferred music on Pandora	Anxiety	Maternal satisfaction scale for cesarean sections	NR	Shafqat 2024
				Intervention 2 (50): Music listening before, during and after cesarean section; preselected Mozart sonatas				
				Control (50): Not specified				
Drzymalski 2017	US	RCT	Pregnant women scheduled for labor induction	Intervention (50): Music listening via broadcast during labor analgesia; participant choice of preferred music on Pandora	Anxiety<br>Pain	Numeric rating scales	NR	Shafqat 2024
				Control (49): Not specified				
Ebneshahidi and Mohseni 2008		RCT	Pregnant women with planned cesarean sections	Intervention (38): Music listening with a soft open-air headphones and a tape player within 15mins of arrival to PACU; 1 session (30mins); participant choice of music	Anxiety<br>Pain	VAS-A<br>VAS-P	NR	Hakimi 2021; Weingarten 2021
				Control (39): Headphones with no music				

Eren 2018	Turkey	RCT	Pregnant women having a planned cesarean section	Intervention (30): Music listening during cesarean section; participant choice of music prior to operation<br>Control (30): Not specified	Anxiety<br>Vital signs	VAS<br>Systolic and diastolic blood pressure	NR	Hunter 2023; Shafqat 2024; Weingarten 2021
Estrella-Juarez 2023	New Zealand	RCT	Nulliparous pregnant women at full term (>37wks) having a vaginal, instrumental or cesarean section birth	Intervention 1 (109): Music listening via iPod with overhead headphones: 20 minute intervals during the first stage of labor; instrumental of music pre-selected by researcher<br>Intervention 2 (130): virtual reality during first stage of labor<br>Control (124): Routine care	Anxiety<br>Vital signs	STAI<br>Systolic and diastolic blood pressure	NR	Hunter 2023; Shafqat 2024
Fleury 2021	Brazil	RCT	Women undergoing fertility treatment	Intervention (50): Music therapy using small and easy to play percussive musical instruments, a guitar, voice, and a flute: once for 50 min<br>Control (50): No intervention	Anxiety<br>Stress	DASS 21<br>LSSI	NR	Kizilkaya 2024
Gaden 2022	Argentina, Colombia, Israel, Norway, and Poland	RCT	Postpartum women	Intervention (105): Music therapy with a music therapist in the NICU: 3 session per week (30mins) for a total of 27 sessions: participant selection of preferred music<br>Control (108): Routine care	Anxiety<br>Depression	GAD-7<br>EPDS	NR	Han 2024
Gan 2016	China	RCT	Pregnant with threatened abortion	Intervention 1 (60): Western medicine and medicinal diet and five-element music: Volume <75db, twice weekly for two weeks for 30 min<br>Intervention 2 (60): Western medicine and five-element music: Volume <75db, twice weekly for two weeks for 30m<br>Intervention 3 (60): Western medicine and medicinal diet<br>Control (60): Routine care (western medicine)	Anxiety<br>depression	Self-rating anxiety scale<br>Self-rating depression scale	Before, after 2 weeks	Wu 2020
Garcia González 2017	Spain	RCT	Nulliparous pregnant women: >28 wks gestation, medically low-risk	Intervention (204): Music listening of recorded music on a CD player: 3 sessions a week (40mins) with 14 sessions in total at home and during non-stress test; instrumental music preselected by researcher (60-75 beats/min)<br>Control (205): Not specified	Anxiety<br>Vital signs	STAI<br>Systolic and diastolic blood pressure	Before and after non-stress test	Dogan-Gangal 2024; Ji 2024; Hunter 2023; Maul 2024

Garcia-Gonzalez 2018	Spain	RCT	Pregnant women in 3rd trimester	Intervention (204): Music listening of recorded music on a CD player: 3 sessions a week (40mins) with 14 sessions in total at home and during non-stress test; instrumental music preselected by researcher (60-75 beats/min)	Anxiety	STAI	NR	Hunter 2023, Ji 2024, Lin 2019, Maul 2024, Shafqat 2024
				Control (205): Not specified				
Gokduman 2022	Turkey	RCT	Primiparous women who had vaginal delivery	Intervention (40): classical Turkish music with sterile virtual reality glasses	Pain	VAS-P	Immediately post-episiotomy and 1h later	Maleki 2023
				Control (44): Routine care				
Gönenç and Dikmen 2020	Turkey	RCT	Nulliparous pregnant women having a vaginal birth	Intervention 1 (33): Music listening using headphones once cervical dilation reached 4-5cm (active labor); 1 session (30mins); subjects choice of 3 songs with a range of upbeat pop music, slow pop music, Turkish folk music, and religious music	Pain, Pregnancy-related Anxiety	VAS<br>W-DEQA	NR	Hunter 2023, Sen 2023
				Intervention 2 (33): Dance and music listening				
				Control (33): Routine care				
Guerrero 2012	USA	RCT	Women undergoing an abortion	Intervention (54): Music listening during vacuum aspiration abortion procedure; participant choice of 10 preloaded playlists	Anxiety<br>Vital signs<br><br>Pain	STAI<br>Systolic and diastolic blood pressure, HR, RR<br>VAS	Before and after the procedure	Lin 2019
				Control (47): Routine care				
Guo 2022 [a]	China	RCT	Primiparous women having a vaginal birth	Intervention (201): Music therapist session to personalize music followed by music listening during all phases of labor: first phase of labor relaxing/hypnotic music was played, late stage of the first phase of labor: intense rhythmic music was played, second/third phase of labor parent–child music was played	Pain	Perception of labour pain questionnaire, perineal pain score	NR	Hunter 2023; Ji 2024, Sen 2023
				Control (239): standard labor practices with no music				
Halder 2022	India	RCT	Pregnant women scheduled for an elective cesarean section	Intervention (30): Music listening using headphones during the preoperative, intraoperative, and postoperative stages of the cesarean section; intervals of 20 mins; participant choice of preferred	Pain	VAS	NR	Hunter 2023

				genre or Indian classical, semi-classical, folk, light music, or instrumental				
				Control (30): Routine care				
Hanprasertpong 2016	Thailand	RCT	Pregnant women 15-21wks gestation who underwent a second trimester genetic amniocentesis	Intervention (161): Music listening using earphones throughout antiseptic skin preparation to the needle removal stage	Anxiety<br>Pain	VAS-A<br>VAS-P	NR	Shafqat 2024
				Control (171): Not specified				
Hepp 2018	Germany	RCT	Pregnant women having a cesarean section	Intervention (154): Music listening using CD player in operating theater; participant choice of classical, jazz, lounge, or meditation music with tempo at 60-80 bpm	Anxiety	VAS-A, STAI	NR	Hunter 2023; Ji 2024; Shafqat 2024; Weingarten 2021
				Control (150): Routine care				
Hinesley 2020		RCT	Pregnant women: 2nd-3rd trimester, medically low risk	Intervention (23): Composing a lullaby and listening teach recorded lullaby: 3 sessions (12hrs total); music created by subjects	Stress	Mental Health: SCL-27 and Pregnancy Stress Scale	NR	Dogan-Gangal 2024
				Control (21): Not specified				
Hoegholt 2024	De--ark	RCT	Pregnant nulliparous women: >25th week gestation	Intervention (31): Music listening and web-based advice on sleep hygiene: Daily sessions (30mins) for 28 days; participant selected music	Sleep quality	PSQI, Insomnia Severity Index.	NR	Hoffman 2025
				Control (40): Web-based advice on sleep hygiene				
Horasanli and Demirbas 2022	Turkey	RCT	Pregnant women with singletons over 37 wks gestation scheduled for elective cesarean section	Intervention (26): Music listening using an earpiece prior to administration of spinal anesthesia and throughout the entire cesarean section; pre-selection of sufi music with steady rhythm	Anxiety<br>Vital signs	STAI<br>Systolic and diastolic blood pressure, HR, RR, O2 sat	NR	Hunter 2023
				Control (23): Routine care				
Hosseini 2013	Iran	RCT	Pregnant women	Intervention (15): Music listening during labor; 2 sessions (30mins) within first two hours; pre-selected music by researchers: baraneeshgh (love rain) composed by Manouchehr cheshmazar	Anxiety<br>Pain	VAS<br>VAS-P Numerical pain rating scale, verbal pain rating scale	NR	Chuang 2018; Ji 2024;
				Control (15): Routine care				
Haung 2010	China	RCT	Women diagnosed with postpartum depression	Intervention (82): light music every other day for 6 weeks for 30m	Depression	HAM-D	NR	Yang 2019
				Control (80): Psychological treatment, drug treatment				

Kafali 2011	Turkey	RCT	Pregnant women: 36 wks gestation, medically low risk	Intervention (96): Music listening of recorded music during non-stress test at hospital: 1 session (30mins); participant's preference or selection among 3 types of music: classical, Turkish art, and Turkish folk music with 60-72 beats/min	Anxiety	STAI	Before and after non-stress test	Dogan-Gangal 2024; Lin 2019
				Control (105): Not specified				
Kakde 2023	Singapore	RCT	Pregnant women having an elective cesarean section	Intervention (53): Music listening using earphones during administration of spinal anesthesia and continuing during the cesarean section and post-surgery in the post-anesthesia care unit: 2 sessions (30mins)	Anxiety<br>Pain	VAS-A<br>Unspecified pain measurement tool	NR	Shafqat 2024
				Control (55): Routine care				
Karkal 2017	India	RCT	Primiparous women	Intervention (30): Music intervention	Anxiety<br>Pain	NR<br>NR	NR	Chuang 2018
				Control (30): Routine care				
Kaur 2023		RCT	Pregnant women having a cesarean section	Intervention (30): Music listening using headphones during cesarean section while under spinal anaesthesia; participant choice of folk, Hindi, film music, or religious songs	Anxiety	VAS-A; cortisol levels; hemodynamic parameters	NR	Hunter 2023
				Control (30): Routine care with headphones worn				
Kehl 2020	Switzerland	RCT	Postpartum women	Intervention (10): Music therapy with a music therapist in the NICU: 2-3 sessions per week (20mins) for a total of 8 sessions	Anxiety<br>Depression<br>Stress	STAI<br>EPS<br>PSS	NR	Han 2024
				Control (6): Routine care				
Kimber 2008	England	RCT	Pregnant women having a vaginal birth	Intervention 1 (30): Music with relaxation at the hospital; subjects were trained to focus on breathing and visualization techniques along with music listening during labor	Anxiety	VAS-A Cambridge birth worry scale, Labour agentry scale	NR	Hunter 2023; Chehreh 2023; Sen 2023
				Intervention 2 (30): Massage with relaxation at the hospital: subjects were trained to focus on breathing and visualization techniques along with massage during labor				
				Control (30): Routine care				
Kirca 2020	Turkey	RCT	Primiparous women who had vaginal delivery	Intervention (50): Turkish music (mother's preference) during episiotomy repair for 15-20m	Pain	VAS-P	Vaginal delivery, hymen	Maleki 2023

				Control (50): Routine care			repair, skin repair, immediately post-episiotomy repair and 1h later	
Kittithanesuan 2017	Thailand	RCT	Mothers with newborns	Intervention (152): Passive listening to regional music on a CD player: once for 11m	Breastmilk volume	ml	NR	Duzgun 2020
				Control (152): Standard nursing care				
Kobus 2022	Germany	RCT	Postpartum women	Intervention (40): Music therapy with a music therapist at the NICU: 2 sessions per week (10-50mins) until discharge	Depression	Allgemeine Depressions Skala, German version of the Center for Epidemiologic Studies Depression Scale	NR	Han 2024; Ji 2024
				Control (40): Routine care				
Küçükkaya 2024	Turkey	RCT	Women within 3h postpartum of full-term delivery	Intervention (41): Music listening at hospital of recorded Turkish music: 2 times a day for 36hrs (30 mins)	Depression	EPDS, Stein Blues Scale	Baseline, 12th hr post partum, 24th hr post partum, post-intervention	Han 2024; Sun 2024
				Control (41): Routine care				
Kumarilohar2018	India	NR	Pregnant women in labor	NR	Pain	VAS-P	NR	Chehreh 2023
Kurdi and Gasti 2018	India	RCT	Pregnant women having an emergency cesarean section	Intervention 1 (63): Music listening using a MP3 player and bilateral headphones covering entire ear during cesarean section; pre-selection of calming and soothing meditation music	Anxiety<br><br>Pain	VAS-A, psychological wellbeing questionnaire; VAS-P	NR	Hunter 2023; Weingarten 2021
				Intervention 2 (63): Music listening using a MP3 player and bilateral headphones covering entire ear during cesarean section; pre-selection of binaural beat meditation music				
				Control (63): Routine care while wearing headphones				
Kwun and Kim 2000	Korea	RCT	Pregnant women having planned cesarean section	Intervention (32): Music listening during preoperative period of cesarean section; 3 sessions (30mins); participant choice of music	Anxiety	STAI	NR	Weingarten 2021
				Control (32): Routine care				

Labrague 2013	Philippines	RCT	Pregnant women in latent labor	Intervention (25): Classical nature music through headphones and speakers for 30 min	Pain during labor	VAS	NR	Chehreh 2023, Sen 2023
				Control (25): Not specified				
Lee 2010	Korea	RCT	Women diagnosed with postpartum depression	Intervention (30): preferred music daily for 8 days for 40 min	Depression	BAI	NR	Yang 2019
				Control (30): Routine care				
Li J 2016	China	RCT	Pregnant with threatened abortion	Intervention (98): Five-element music (Gong (10 min), Shang (5 min), Jue (10 min), Zhi (5 min))	Anxiety<br>Depression	Self-rating anxiety scale<br>Self-rating depression scale	After intervention	Wu 2020
				Control (98): Routine care				
Li Z 2015	China	RCT	Postpartum	Intervention (40): electroacupuncture and five-element music	Depression	Self-rating depression scale, HAM-D	Before, after (3weeks, 6weeks)	Wu 2020
				Control (42): Acupuncture				
Li Z 2016a	China	RCT	Pregnant with threatened abortion	Intervention 1 (60): Western medicine and medicinal diet and five-element music: Volume <75db, twice weekly for two weeks for 30 min	Anxiety<br>Depression	HAM-A<br>HAM-D	Before, after 2 weeks	Wu 2020
				Intervention 2 (60): Western medicine and medicinal diet				
				Intervention 3 (60): Western medicine and five-element music: Volume <75db, twice weekly for two weeks for 30m				
				Control (60): Routine care (western medicine)				
Li Z 2016b	China	RCT	Pregnant with threatened abortion	Intervention (120): Five-element music: Volume <75db, twice weekly for two weeks for 30m	Anxiety<br>depression	Self-rating anxiety scale<br>Self-rating depression scale	Before, after 1,2 weeks	Wu 2020
				Control (120): Routine care				
Li and Dong 2012	China	RCT	Pregnant women scheduled for elective cesarean section	Intervention (30): Music listening for 30 minutes before and throughout the entire cesarean section; participant choice of classical Chinese music	Anxiety	Self-rating anxiety scale, heart rate variability, VAS	NR	Hunter 2023; Shafqat 2024; Weingarten 2021
				Control (30): Routine care and 30 minutes of relaxation before cesarean section				
Liu 2010	Taiwan	RCT	Primiparous women having a vaginal birth	Intervention (30): Music listening during the latent and active phases of labor for 30 minutes with or without headphones: participant choice of relaxing music, light music, popular music, crystal, children's, or Chinese religious music.	Anxiety<br>Pain	VAS-A<br>VAS-P	During active and latent phases of labor	Chuang 2018; Hunter 2023; Shafqat 2024, Sen 2023

				Control (30): Routine care				
Liu 2014	China	RCT	Women diagnosed with postpartum depression	Intervention (41): Pure music every other day for 3 days for 1-2 hours<br>Control (41): Health education, psychological treatment	Depression<br>sleep	Self-rating depression scale<br>PSQI	NR	Yang 2019
Liu 2016	Taiwan	RCT	Sleep-disturbed pregnant women: 18-34 wks gestation	Intervention (61): Music listening of recorded music at bedtime: daily for 2 weeks (30mins); participant selected from their own collection or from 5 categories: lullaby, classical, nature sounds, crystal, or symphonic music with 60-80 beats/min<br>Control (60): Standard prenatal care	Anxiety<br>Sleep quality<br>Stress	STAI<br>PSQI<br>PSS	Baseline and post-intervention	Dogan-Gangal 2024; Ji 2024; Hoffman 2025; Lin 2019; Maul 2024 ; Shafqat 2024
Liu 2017	China	RCT	Pregnant women	Intervention (50): five-element music: twice daily for 8 weeks for 30m<br>Control (50): Routine care	Anxiety<br><br>depression	Self-rating anxiety scale<br>Self-rating depression scale	Before, after 6 weeks, 1 week after birth	Wu 2020
Mohd Shukri 2019	Malaysia	RCT	Mothers with newborns	Intervention (33): Passive listening to image-supported relaxing music: once daily for 2 weeks (14 total sessions)<br>Control (31): Standard nursing care	Breastmilk volume	ml	NR	Duzgun 2020
Momeni 2020	Iran	RCT	Pregnant women 37-42wks gestation	Intervention (65): Creating a calming enviro--ent: Snoezelen's room designed using an aquarium, and a projector, which played optical shapes, light music, and essential aroma<br>Control (65): Not specified	Anxiety	VAS-A	NR	Shafqat 2024
Moragianni 2009	USA	RCT	Women undergoing fertility treatment	Intervention (67): Harp music therapy with embryo transfer: once for 20m<br>Control (59): Standard care	Anxiety	STAI	NR	Kizilkaya 2024
Murphy 2014	USA	RCT	Women undergoing fertility treatment	Intervention (90): Harp music therapy with embryo transfer: once for 20m<br>Control (91): Standard care	Anxiety	STAI	NR	Kizilkaya 2024
Nandeibam 2022	India	RCT	Women undergoing fertility treatment	Intervention (54): Instrumental music (Zebronics, Zeb-thunder) through headphones for 30-45m<br>Control (55): Noise-cancelling headphones	Pain<br>Anxiety	VAS<br>Anxiety score	NR	Kizilkaya 2024
Nayak 2014	India	NR	Pregnant women in labor	NR	Pain	VAS-P	NR	Chehreh 2023
Nikandish 2007		RCT	NR	Intervention (50): Spanish guitar<br>Control (50): White music	Anxiety<br>Pain	VAS-A<br>VAS-P	NR	Hakimi 2021

Norouzi 2013	Iran	RCT	Women diagnosed with postpartum depression after cesarean section	Intervention (30): Lullaby daily for 30 min	Anxiety	STAI	NR	Yang 2019
				Control (30): Kangaroo care				
Nwebube 2017	UK	RCT	Pregnant women	Intervention (20): Music listening of traditional lullabies at home: Daily sessions (20mins) for 12 weeks	Anxiety<br>Depression	STAI<br>EPDS	Baseline and post-intervention	Han 2024; Lin 2019; Maul 2024; Shafqat 2024; Sun 2024
				Control (16): Routine care				
Orak 2020	Turkey	RCT	Women undergoing fertility treatment	Intervention 1 (31): Turkish classical music through headphones 1h before surgery: once for 60 min	Pain	VAS	NR	Kizilkaya 2024
				Intervention 2 (31): Turkish classical music through headphones 1h before surgery and continuing through surgery: 60+ min				
				Control (31): No music				
Palazzi 2021	Brazil	RCT	Postpartum women	Intervention (22): Music therapy with a music therapist at the NICU: 2 sessions per week (20-30 mins) for a total of 6 sessions; participant selection of preferred music	Anxiety<br>Depression<br>Stress	STAI<br>EPDS<br>PSS	NR	Han 2024
				Control (17): Routine care				
Parodi 2021	Italy	RCT	Low-risk pregnant women having an elective cesarean section	Intervention (40): Music listening of novel binaural beat technique audio track for deep relaxation during the preoperative period (within 1hr of cesarean section): 1 session (12 mins)	Anxiety	STAI	NR	Shafqat 2024; Weingarten 2021
				Control (20): Routine care				
Perkins 2023	UK	RCT	Women within 9 months postpartum	Intervention (44): Music therapy with a musically trained research assistants at home: 60-minute sessions for 6 weeks; frequency of sessions NR : participant selection of preferred music	Depression	EPDS, Social support and loneliness	Baseline, immediate post-intervention, 4 weeks post-intervention	Han 2024; Sun 2024
				Control (45): Routine care				
Perkovic 2021	Bosnia	RCT	Pregnant women in second and third trimesters	Intervention (90): Group education intervention with music listening: daily sessions (15min) before bedtime during pregnancy; participant choice of classical music	Anxiety<br><br>Pain	The symptom checklist—90<br>VAS-P	NR	Hunter 2023; Ji 2024
				Control (85): Routine care				
Phumdoung and Good 2003	Thailand	RCT	Primiparous women having a vaginal birth	Intervention (55): Music listening to soft music with earphones during first 3 hours of the active phase of labor:	Anxiety<br>Pain	VAS-A<br>VAS-P	During active phase of labor	Chuang 2018; Hunter 2023; Sen 2023

				participant choice of five types of Western music without lyrics: synthesizer, harp, piano, orchestra, and jazz.<br>Control (55): Routine care				
Qi 2023	China	RCT	Pregnant women 28-36wk gestation	Intervention (29): Music listening at home: daily sessions (20-30mins) for 4 weeks<br>Control (29): Routine care	Depression	EPDS	Baseline, immediate post-intervention, 6 weeks postpartum	Sun 2024
Rajakumari 2015	India	NR	Pregnant women in labor	NR	Pain	VAS-P	NR	Chehreh 2023
Reza 2007	Iran	RCT	Pregnant women having an elective cesarean section	Intervention 1 (50): Music listening using a CD player and headphones during cesarean section; pre-selection of Spanish-style guitar music<br>Control (50): white noise using CD player and headphones	Anxiety<br>Pain	VAS-A<br>VAS-P	NR	Shafqat 2024; Weingarten 2021
Rezaei 2023	Iran	RCT	Pregnant women 32-41wks gestation	Intervention 1(65): Inhalation of lavender aromatherapy drops and music listening using earphones before a non-stress test; pre-selected nature sounds<br>Intervention 2 (65): Inhalation of a placebo (distilled water) before a non-stress test<br>Control (65): No intervention	Anxiety	STAI	NR	Shafqat 2024
Ribeiro 2018	Brazil	RCT	Mothers of preterm infants admitted to NICUs	Intervention (10): Music sessions performed by professional music therapists at the hospital; weekly sessions (30-45 mins)<br>Control (11): Routine care	Depression	BDI	Baseline and discharge of infant	Sun 2024
Salafas 2022	Indonesia	Quasi-experimental	Pregnant women	Intervention (30): Music listening session (15mins); pre-selected relaxation music with 60 bpm<br>Control (0): None	Anxiety	HAM-A	NR	Ji 2024
Sanfilippo 2020	Gambia	RCT	Pregnant women	Intervention (50): Live music session including singing, moving to the music, and clapping led by medical staff at the clinic: weekly sessions (60 mins) for 6 weeks<br>Control (74): Routine care	Depression	EPDS	Baseline, immediate post-intervention, and 4wks post-intervention	Han 2024; Maul 2024

Sanli 2022	Turkey	RCT	Primiparous singleton pregnant women	Intervention (35): music listening and reminders: Sessions (30mins) 3 times a week for 2 weeks; musical pieces composed in uşşak mode selected by the researchers<br>Control (35): Not specified	Sleep quality	Adjusted PSQI	NR	Hoffman 2025; Ji 2024
Simavli 2014a	Turkey	RCT	Primiparous women who had vaginal delivery	Intervention (77): self-selected music (classical music, light music, popular music, Turkish art music, Turkish folk music and Turkish Sufi music) through headphones during labor for 2h periods with 20m breaks<br>Control (79): Routine care	Anxiety<br>Pain	STAI<br>FAS<br>VAS-A<br>VAS-P	Vaginal Delivery, 1, 4, 8, 16, 24h	Chuang 2018; Hunter 2023; Ji 2024; Maleki 2023 ; Sen 2024 ; Shafqat 2024
Simavli 2014b	Turkey	RCT	Primiparous women having a vaginal birth: 38+ wks gestation	Intervention (80*): Self-selected music listening at the hospital during labor: Non-stop music with 20min break every 2hrs until end of third phase of labor with or without headphones; participant choice of six types of music, including classical music, light music, popular music, Turkish art music, Turkish folk music, and Turkish Sufi music.<br>Control (81): Routine care	Anxiety<br>Depression<br>Pain	VAS-A<br>EPDS<br>VAS-P	During active and latent phases of labor, at vaginal delivery, 1h postpartum	Hakimi 2021; Han 2024 ; Hunter 2023; Ji 2024; Maleki 2023 ; Shafqat 2024; Sun 2024; Yang 2019
Sharifi 2013	Iran	RCT	Pregnant women having a cesarean section	Intervention (30): Music listening during preoperative period (within 2hrs of cesarean section): 1 session (20mins); pre-selected music by researchers including instrumental music or recitation of the Quran<br>Control (15): Routine care	Anxiety	STAI	NR	Weingarten 2021
Shin & Kim 2011	South Korea	CCT	Pregnant women: <14 weeks gestation, medically low risk	Intervention (117): Listening of recorded music for 1 session of 30mins during transvaginal ultrasound; music chosen by researchers (nature sounds)<br>Control (116): Transvaginal ultrasound without music	Anxiety<br>Stress	STAI<br>PSS	Baseline and immediately post intervention	Corbijn van Willenswaard 2017; Dogan-Gangal 2024
Shobeiri 2016	Iran	RCT	Singleton pregnant women: 30-34th week gestation with sleep disorder	Intervention (42): Music listening and two weekly sessions of music therapy counseling in groups: Daily sessions (45mins) for 28 days; music selected by the researchers<br>Control (44): Not specified	Sleep quality	PSQI	NR	Hoffman 2025

Soylu 2022	Turkey	RCT	Pregnant women	Intervention (37): Music listening using a MP3 player and earphones during a nonstress test; 1 session (30mins); participant selection of preferred music<br>Control (37): Not specified	Anxiety	STAI	NR	Shafqat 2024
Stocker 2016	UK	RCT	Women undergoing fertility treatment	Intervention (21): Music of choice through headphones: once for 15m<br>Control (21): No music	Anxiety	STAI	NR	Kizilkaya 2024
Su 2014	China	RCT	Women diagnosed with postpartum depression	Intervention (80): pure music twice daily for 6 weeks for 30m<br>Control (80): Psychological treatment, traditional Chinese medicine	Depression	NR	NR	Yang 2019
Surucu 2018	Turkey	RCT	Healthy pregnant primiparous women having a vaginal birth	Intervention (25): Music listening in active labor (4cm) through headphones for 3 hours, alternating between 20 mins of listening and 10 mins break: acemasiran music selected for the participant<br>Control (25): Routine care	Anxiety<br><br><br>Pain	VAS-A, STAI, FAS (face anxiety scale)<br>Pain perception	NR	Hunter 2023; Ji 2024; Shafqat 2024, Sen 2023
Suryani 2021	Indonesia	RCT	Nulliparous pregnant women in active labor	Intervention (30): Instrumental music<br>Control (30): Not specified	Pain<br>Anxiety	VAS<br>STAI	NR	Sen 2023
Taghinejad 2010	Iran	RCT	Primiparous pregnant women with singleton pregnancies having a vaginal birth	Intervention 1 (50): Music listening with headphones during early active phase of labor: 1 session (30mins); participant choice of five types of soft traditional music without lyrics<br>Intervention 2 (51): Massage during early active phase of labor	Anxiety	VAS-A	NR	Hunter 2023; Chehreh 2023
Teckenberg-Jansson 2019	Finland	RCT	Women hospitalized with pregnancy-related complication	Intervention (52): Music therapy with music therapist playing two lyre instruments and humming at hospital bedside; Daily sessions (30mins) for 3 days<br>Control (50): Routine care	Anxiety<br>Stress	STAI<br>PSS	Baseline and post-intervention	Lin 2019; Maul 2024; Shafqat 2024
Toker and Komurcu 2017	Turkey	RCT	Pregnant women with preeclampsia (≥30 wks gestation)	Intervention (35): Music listening using MP3 player and headphones and deep breathing at hospital: daily sessions (30mins) for 5 days before and 2 days after labor; participant selected from 2 categories: Nihavend or Buselik modes<br>Control (35): Not specified	Anxiety<br>Vital signs	STAI<br>Systolic and diastolic blood pressure	Baseline and 5th day of the intervention	Dogan-Gangal 2024; Lin 2019; Maul 2024; Shafqat 2024
Tseng 2010		RCT	NR	Intervention (37): Music therapy	Anxiety	STAI	NR	Hakimi 2021

				Control (40): Usual care	Stress	PSS		
Ventura 2012	Portugal	Quasi-experimental	Pregnant women having a singleton pregnancy, low risk, waiting for amniocentesis	Intervention (154): Music listening using CD player while sitting and reading magazines; sessions 30 mins; participant choice of 4 types of music<br>Control (0): None	Anxiety	STAI	NR	Shafqat 2024
Vianna 2011	Brazil	RCT	Mothers with newborns	Intervention (48): Passive and active listening (playing instruments and singing): once daily for 3 days (3 total sessions) for 60 min<br>Control (48): Standard nursing care	Breastfeeding rate	NR	NR	Duzgun 2020
Wan and Wen 2018	China	RCT	Pregnant women having a vaginal birth	Intervention 1 (60): Music listening during labor and delivery: 20 min sessions followed by a 2-hour break<br>Intervention 2 (60): Acupressure during labor and delivery<br>Intervention 3 (62): Music listening and acupressure during labor and delivery<br>Control (59): Routine care	Anxiety<br>Pain	VAS-A<br>VAS-P	NR	Hunter 2023
Wang 2016a	China	RCT	Postpartum	Intervention (200): Five-element music: 40db, twice daily for 3 days for 30 min<br>Control (200): Routine care	Anxiety<br>Depression	Self-rating anxiety scale<br>Self-rating depression scale	24 and 72 h post-birth	Wu 2020
Wang 2016b	China	RCT	Women diagnosed with postpartum depression after cesarean section	Intervention 1 (200): Pure music every other day for 3 days for 30 min<br>Intervention 2 (200): Psychological treatment<br>Control (52): Kangaroo care	Anxiety<br>Depression	Self-rating anxiety scale<br>Self-rating depression scale	NR	Yang 2019
Wang 2018	China	RCT	Postpartum	Intervention (60): Five-element music: 20-40db, once daily for 8 weeks for 30-60 mIN<br>Control (60): routine care	Depression	HAM-D<br>EDPS	Before, after 8weeks	Wu 2020
Wang 2019	China	RCT	Pregnant in labor	Intervention 1 (53): Electroacupuncture<br>Intervention 2 (55): Electroacupuncture and five-element music: 30m every 2h throughout labor<br>Intervention 3 (54): Medicine<br>Control (51): No intervention	Pain<br>Length of labor<br>Blood loss	VAS<br>Mins<br>ml<br>Serum dynomorphin	Before, after	Wu 2020
Wei 2013	China	RCT	Postpartum	Intervention (60): Five-element music: daily for 8 weeks for 30m<br>Control (60): Medicine	Depression	EPDS	Before, after (2, 4, 6, 8 weeks)	Wu 2020

Wu 2012	USA	RCT	Women undergoing an elective abortion	Intervention (13): Music listening during surgical abortion procedure; participant choice of 5 preloaded playlists<br>Control (13): Routine care	Anxiety<br>Pain	11-point verbal numerical scales of anxiety and pain, STAI	Baseline, prior to pelvic exam, during surgical procedure, after speculum removal, and 30mins after the procedure;	Lin 2019; Shafqat 2024
Wulff 2021a	Germany	RCT	Pregnant women: 24-36wks gestation	Intervention 1 (64): A 30-min group music session was led by a music therapist with up to 3 other women between 30 and 34th wk gestation to practice relaxation through passive music listening and instruction on how to listen and relax to music at home; subjects received a CD with classical, calm music without lyrics and a soothing calm beat to listen to at home for 10-15 mins a day until birth, subjects were also free to choose and listen to other music<br>Intervention 2 (59): Two 30-minute group singing sessions were led by a music therapist with up to 7 other women between 30th and 34th wk gestation; music therapist practiced children's songs and lullabies with the group and played the guitar; subjects received a song book with lyrics and melodies of 10 children's songs and lullabies and asked to continue sessions on a daily basis at home for 10-15 mins a day until birth<br>Control (49): Routine care	Anxiety<br>Depression<br>Emotional state<br>Maternal attachment	STAI<br>EPDS<br>VAS<br><br>Maternal Antenatal Attachment Scale	30th and 36th gestational week in intervention groups (before and after intervention)	Han 2024; Maul 2024
Wulff 2021b	Germany	RCT	Postpartum women	Intervention (59): Up to three 45-minute music sessions led by a music therapist with 5-10 women and their babies at the hospital between 3 and 12 weeks postpartum, including elements of finger games, lullabies and movements to music; Subjects asked to complete	Anxiety<br>Depression<br>Emotional state<br>Maternal attachment	STAI<br>EPDS<br>VAS<br><br>Postpartum bonding questionnaire	Baseline (within 48hrs of delivery), 2 weeks postpartum, 12 weeks postpartum	Sun 2024

				singing and music-based interaction between mother and baby daily at home; standard repertoire of songs and games were provided along with subjects' requests				
				Control (61): Routine care				
Xavier 2016	India	NR	Pregnant women in labor	NR	Pain	VAS-P	NR	Chehreh 2023
Xu 2017	China	RCT	Pregnant in labor	Intervention (50): five-element music: volume 40 db, 30 min every 2h for duration of labor	Anxiety	Self-rating anxiety scale	Before, 24h after birth	Wu 2020
				Control (50): Routine care				
Yang 2009	China	RCT	Pregnant women: 28-36 wks gestation, medically high risk	Intervention (60): Usual care plus listening to music for 3 days for 30minutes/day in hospital; participant selected among 3 types of music: classical music, pleasant music, and Chinese folk music with 60-72 beats/min	Anxiety<br>Vital signs	STAI<br>Systolic and diastolic blood pressure, heart rate, respiratory rate	Before and 2hrs after final session	Corbijn van Willenswaard 2017; Dogan-Gangal 2024; Lin 2019; Maul 2024
				Control (60): Usual care				
Yüksekol 2020	Turkey	RCT	Pregnant women 28-32wks gestation hospitalized with preeclampsia	Intervention (30): Music listening at the hospital: 2 sessions (30mins) a day (morning and evening)	Anxiety<br>Vital signs	STAI<br>Systolic and diastolic blood pressure	NR	Shafqat 2024
				Control (30): No music				
Zou 2018	China	RCT	Postpartum	Intervention 1 (62): acupressure and five-element music: 40-60db, twice daily for 3 days for 30 min	Anxiety<br><br>Milk production	Self-rating anxiety scale<br>Milk yield<br>Breast fullness	Before, 72 h post-birth	Wu 2020
				Intervention 2 (62): Acupressure				
				Control (62): Five-element music: 40-60 db, twice daily for 3 days for 30 min				

Notes:
[a] Guo 2022 has been retracted. We will not present any analyses with these data.
