# Supplementary material for: Music interventions to improve women’s health outcomes in the preconception, antepartum, intrapartum, and postpartum periods: An overview of reviews
Source: PLoS One. 2026 Feb 18;21(2):e0339337. doi: 10.1371/journal.pone.0339337 (PMC12915951; doi:10.1371/journal.pone.0339337)
Supplement: S3 Table — (PDF) [file pone.0339337.s003.pdf]

Supplementary Materials

Table S3: Primary Study Mapping

Primary studies	Eligible Reviews [a]																					
	1	2	3	4	5	6	7	8	9	10	11	12	13	14	15	16	17	18	19	20	21	22	23
Aba 2017											X			X									
Abarghoee 2022									X														
AjorPaz 2010																					X		
Allameh 2013																					X		
Ak 2015					X																		
Akmese and Oran 2014				X																			
Almedhesh 2022																		X					
Amanak 2020									X										X				
Angın 2020															X								
Arya 2012												X											
Bansal 2019																					X		
Baltaci 2023																X		X					
Bose 2015																							
Bershadsky 2014																							
Bauer 2010			X	X																			
Browning 2001									X														
Buglione 2020									X	X					X			X	X				
Cao 2016													X			X		X		X			
Cappon 2014				X																			
Çatalgöl 2022										X		X				X		X					
Chaichanalp 2018a															X								
Chaichanalp 2018b															X								
Chang 2005									X									X			X		
Chang 2008			X	X			X						X			X		X		X			
Chang 2015			X	X												X							
Chengshu 2018																						X	

Primary studies	Eligible Reviews [a]																						
	1	2	3	4	5	6	7	8	9	10	11	12	13	14	15	16	17	18	19	20	21	22	23
Cheung 2018											X			X									
Choubsaz 2018																					X		
Dabas 2019					X																		
Davis 2014																							
Dehcheshmeh and Rafiei 2015									X								X		X				
Denney 2018									X												X		
Dereddy 2024							X																
Drzymalski 2023																		X					
Drzymalski 2020																		X					
Drzymalski 2017																		X					
Dolker 2019										X													
Ebneshahidi and Mohsen 2008						X															X		
Eren 2018									X									X			X		
Estrella-Juarez 2023									X									X					
Fleury 2021											X			X									
Gaden 2022							X																
García-González 2018a				X					X	X		X	X			X		X					
García-González 2018b									X							X							
García-González (2017)										X													
Gokduman 2022															X								
Gönenç and Dikmen 2020									X										X				
Guerrero 2012													X										
Guo 2022									X	X									X				
Halder 2022									X														
Hanprasertpong 2016																		X					
Hepp 2018									X	X		X						X			X		
Hinelsey 2020				X																			

Primary studies	Eligible Reviews [a]																						
	1	2	3	4	5	6	7	8	9	10	11	12	13	14	15	16	17	18	19	20	21	22	23
Hoegholt 2024								X															
Horasanlı and Demirbas 2022									X														
Hosseini 2013		X								X							X						
Huang 2010																							X
Jiawei 2016																						X	
Kafali 2011				X								X	X										
Kakde 2023																		X					
Karkal 2017		X																					
Kaur 2023									X														
Kehl 2021							X																
Khoshkholgh 2016				X																			
Kimber 2008	X								X								X		X				
Kırca 2020															X								
Kittithanesuan 2017					X																		
Kobus 2022							X																
Kobus 2023										X													
Küçükkaya 2024							X													X			
Kumarilohar 2018	X																						
Kurdi and Gasti 2018									X												X		
Kwun and Kim 2000																					X		
Labrague 2013																			X				
Lee 2010																							X
Li and Dong 2012									X								X	X			X		
Liu 2010		X							X								X	X	X				
Liu 2014																							X
Liu 2016				X				X		X		X	X			X		X					
Mengying 2019																						X	
Mengyan 2017																						X	

Primary studies	Eligible Reviews [a]																						
	1	2	3	4	5	6	7	8	9	10	11	12	13	14	15	16	17	18	19	20	21	22	23
Mohd Shukri 2019					X																		
Momeni 2020																		X					
Moragianni 2009											X			X									
Murphy 2014											X			X									
Nandeibam 2022											X												
Nayak 2014	X																						
Nikandish 2007						X																	
Norouzi 2013																							X
Nwebube 2017							X						X			X		X		X			
Orak 2020											X			X									
Palazzi 2021							X																
Parodi 2021																		X			X		
Perkins 2023							X													X			
Perkovic 2021									X	X													
Phumdoung 2003		X							X								X		X				
Ping 2015																						X	
Qi 2023																				X			
Reza 2007																	X	X			X		
Abhari Rezaei 2005	X																						
Abhari Rezaei 2023																		X					
Ribeiro 2018																				X			
Sanfilippo 2020							X									X							
Sanli 2022								X		X													
Sharifi 2013																					X		
Simavli 2014		X				X	X		X	X					X		X	X	X	X			X
Shin & Kim 2011			X	X																			
Shoberi 2016								X															
Soylu 2022												X						X					
Stocker 2016											X			X									
Su 2014																							X

Primary studies	Eligible Reviews [a]																						
	**1**	**2**	**3**	**4**	**5**	**6**	**7**	**8**	**9**	**10**	**11**	**12**	**13**	**14**	**15**	**16**	**17**	**18**	**19**	**20**	**21**	**22**	**23**
Surucu 2018									X	X		X					X	X	X				
Suryant 2021																			X				
Taghinejad 2010	X								X								X						
Teckenberg 2019													X			X		X					
Toker and Komurcu 2017				X									X			X		X					
Tseng 2010						X																	
Ventura 2012												X						X					
Vianna 2011					X																		
Wan and Wen 2018									X														
Wang 2016																						X	X
Wenhui 2017																						X	
Wu 2012													X					X					
Wulff 2021							X									X				X			
Wulff 2021b							X									X				X			
Xavier 2016	X																						
Xiaofeng 2018																						X	
Yang 2009			X	X									X										
Yanwei 2016																						X	
Yüksekol 2020																		X					
Zhen 2015																						X	
Zhiyun 2016a																						X	
Zhiyun 2016b																						X	

Notes:
[a] The following reviews are mapped: 1. Chehreh 2023, 2. Chuang 2018, 3. Corbijn van Willenswaard 2017, 4. Dogan-Gangal 2024, 5. Duzgun 2020, 6. Hakimi 2021, 7. Han 2024, 8. Hoffmann 2025, 9. Hunter 2023, 10. Ji 2024, 11. Kizilkaya 2024, 12. Konsam 2023, 13. Lin 2019, 14. Mahmoud 2022, 15. Maleki 2023, 16. Maul 2024, 17. Santivanez-Acosta 2020, 18. Shafqat 2024, 19. Sen 2023, 20. Sun 2024, 21 Weingarten 2021, 22. Wu 2020, 23. Yang 2019
