# Supplementary material for: Music interventions to improve women’s health outcomes in the preconception, antepartum, intrapartum, and postpartum periods: An overview of reviews
Source: PLoS One. 2026 Feb 18;21(2):e0339337. doi: 10.1371/journal.pone.0339337 (PMC12915951; doi:10.1371/journal.pone.0339337)
Supplement: S5 Table — (PDF) [file pone.0339337.s005.pdf]

Supplementary Materials

Table S5: Characteristics of Primary Studies Included in Each Review

**Primary study** (Author Year)	**Country**	**Study design**	**Population**	**Description of Trial Arms** (sample)	**Outcomes of interest**	**Outcome Measurement**	**Data collection frequency**	**Review** (Author Year)
Aba 2017	Turkey	RCT	Women undergoing fertility treatment	Intervention (89): A music CD consisting of violin and viola concertos by Bach, Martini, and Mozart, played at 45 dB: once for 28m	Anxiety	STAI	NR	Kizilkaya 2024
				Control (97): No music				
Abarghoee 2022	Iran	RCT	Primiparous pregnant women scheduled for a cesarean section	Intervention 1 (35): Music listening using MP3 player in private room at hospital prior to cesarean section: 1 session (20mins); pre-selected nonverbal song by researchers ("Weightless" by Macaroni Union)	Anxiety	STAI	NR	Hunter 2023
				Intervention 2 (35): Benson relaxation techniques				
				Control (35): Routine care				
Abhari 2000	Iran	NR	Pregnant women in labor	NR	Pain	VAS-P	NR	Chehreh 2023
AjorPaz and Ranjbar 2010	Iran	RCT	Pregnant women having a cesarean section	Intervention (50): Music listening during preoperative period; 1 session (20mins); pre-selected music of sung versus of the Quran	Anxiety	STAI	NR	Weingarten 2021
				Control (30): Quiet room for 20 min				
Ak 2015	India	RCT	Mothers with newborns	Intervention (29): Passive listening to music played on flute: 2x/day for 4 days (8 total sessions) for 30 min	Breastmilk volume	ml	NR	Duzgun 2020
				Control (29): No music				
Akmese and Oran 2014	NR	RCT	Pregnant women with back pain: 12-24 wks gestation	Intervention (33): Progressive muscle relaxation exercises accompanied with low-level music at home: 2 sessions (20mins) a day for 8 weeks: music preselected by researcher	Quality of life	Quality of Life Short Form-36	Baseline and post intervention	Dogan-Gangal 2024
				Control (33): Not specified				
Allameh 2013	Iran	RCT	Pregnant women with singleton gestation having a planned cesarean section	Intervention (31): Music listening using headphones during entire cesarean section and 10 mins after; pre-selection of music by researcher (recited verses of the Quran)	Anxiety	Zung Self-Rating Anxiety Scale	NR	Weingarten 2021

				Control (33): Headphones with no music				
Almedhesh 2022	Saudi Arabia	RCT	Low-risk pregnant women having an elective cesarean section	Intervention (176): Wearing virtual reality glasses during and after regional anesthesia; participant selection between Holy Quran verses with landscapes and music with landscapes	Anxiety<br><br>Satisfaction	Novel visual facial anxiety scale<br>Maternal satisfaction scale	NR	Shafqat 2024
				Control (175): Routine care				
Amanak 2020	Turkey	RCT	Primipara pregnant women having a vaginal birth	Intervention (15): Music listening on a CD player: rounds of 30mins of music followed by 30 mins of break during vaginal delivery; pre-selected Turkish music (instrumental Ney music in modal rhythm of Segah) by researcher	Anxiety	VAS-A, STAI	NR	Hunter 2023, Sen 2023
				Control (15): Routine care				
Angin 2020	Turkey	RCT	Primiparous and multiparous women who had vaginal delivery	Intervention 1 (30): Turkish classical music through speakers from stage 2 to end of episiotomy repair (~90m)	Pain	VAS-P	Immediately post-episiotomy repair	Maleki 2023
				Intervention 2 (30): Turkish pop music through speakers from stage 2 to end of episiotomy repair (~90m)				
				Control (30): Routine care				
Baltacı, 2022	Turkey	RCT	Pregnant women hospitalized due to high risk pregnancy	Intervention (38): Lullabies for 20m while touching abdomen	Anxiety	STAI	NR	Shafqat 2022
				Control (38): Not specified				
Baltacı, 2024	Turkey	RCT	Nulliparous pregnant women 36+ wks gestation	Intervention 1 (40): Music listening using a CD player at home; daily sessions (30mins) for 2 weeks; preselected lullabies	Anxiety	STAI	Baseline and post-intervention	Maul 2024
				Intervention 2 (40): Music listening using a CD player at home; daily sessions (30mins) for 2 weeks; participant selection of nature sounds, Western or Turkish music, or lullabies				
				Control (40): Routine care				
Bansal 2019	India	RCT	Pregnant women having a planned cesarean section	Intervention (30): Music listening using headphones during the entire cesarean section; participant choice of music	Anxiety	VAS	NR	Weingarten 2021
				Control (30): Headphones without sound				
Bauer 2010		RCT	Pregnant women: 24-36 wks	Intervention 1 (19): Music therapy: 1 session (60 minutes) with music	Stress		Baseline, immediately	Corbijn van Willenswaard

			gestation, medically high risk, hospitalized 3+ days	therapist at hospital; music selected by participant among 5 categories		Antepartum bedrest emotional impact inventory	post intervention, and 48-72hrs after intervention	2017; Dogan-Gangal 2024
				Intervention 2 (19): Relaxation intervention: 1 session (60 minutes)				
				Control (42): Waitlist attention group				
Browning 2001		RCT	Primiparous women with planned hospital vaginal birth	Intervention (10): Session with a music therapist to select soothing anxiety-relieving and rhythmic music followed by music listening during labor: six 90-min tapes of recorded music provided to use on cassette player or Walkman	Relaxation and control as proxies for anxiety<br><br>Pain	Labor agentry scale - attitude towards childbirth scale; Trippet objective muscle relaxation inventory McGill pain scale;	NR	Hunter 2023
				Control (10): Routine care				
Buglione 2020	Italy	RCT	Nulliparous women at full-term with singleton pregnancy having a vaginal birth	Intervention (15): Music listening via speakers throughout their labor: participant choice of gentle popular music, soft classical music, and Israeli tunes	Anxiety<br>Pain	VAS-A<br>VAS-P	Vaginal Delivery, episiotomy pain at 1, 24, 48 h	Hunter 2023; Ji 2024; Shafqat 2024, Maleki 2023, Sen 2023
				Control (15): Routine care				
Cao 2016	China	RCT	Pregnant women admitted to hospital with pregnancy-induced hypertension	Intervention (30): Music listening using a MP3 player with headphones for 30-60mins a day for 4 weeks; participant's preferred music or selection from recorded CD	Anxiety<br>Vital signs<br><br>Depression	HAM-A<br>Systolic and diastolic blood pressure<br>HAM-D	Baseline and post-intervention	Lin 2019; Maul 2024; Shafqat 2024; Sun 2024
				Control (30): Routine care				
Cappon 2014		CCT	Anxious pregnant women	Intervention (36): music listening of pre-recorded psychoacoustically modified music preselected by researcher: 20 sessions (15-30 min)	Anxiety	BAI	NR	Dogan-Gangal 2024
				Control (37): Not specified				
Catalgol 2021	Turkey	RCT	Pregnant women 36-38 wks gestation	Intervention (50): Music listening during non-stress test with a MP4 player; participant selection of 12 classical Turkish songs	Anxiety	STAI-S, STAI-T	Before and after non-stress test, before NST at 36 and 38w	Ji 2024; Maul 2024; Shafqat 2024
				Control (50): Not specified				
Chaichanalap 2018	Thailand	RCT	Primiparous and multiparous women who had vaginal delivery	Intervention (50): "A Yellow Brick Cinema-Relaxing Piano Music" through earphones from episiotomy repair to 6h postpartum	Pain	VAS-P	Vaginal delivery, 2h and 6h post-repair	Maleki 2023
				Control (50): Routine care				
Chang 2008	Taiwan	RCT	Pregnant women: 18-22 or 30-34 wks	Intervention (116): Routine ANC and listening to music for 2 weeks for 30minutes/day; participant selection	Anxiety<br>Depression<br>Stress	STAI<br>EPDS<br>PSS	Baseline, immediately	Corbijn van Willenswaard 2017; Dogan-

			gestation, medically low risk	among 4 types of music: lullabies, classical music, nature sounds, and crystal music with 60-80 beats/min<br>Control (120): Routine ANC			post intervention	Gangal 2024; Han 2024; Lin 2019; Maul 2024; Shafqat 2024; Sun 2024
Chang 2015	Taiwan	RCT	Pregnant women: ≥17 wks gestation, medically low risk	Intervention (145): Music listening using a CD, headphones or speakers at home; daily sessions (30mins) for 2 weeks; participant selection among 4 types of music: lullabies, classical music, nature sounds, crystal and symphonic music with 60-80 beats/min<br>Control (151): Routine ANC	Stress	PSS, pregnancy stress rating scale	Baseline and immediately post intervention	Corbijn van Willenswaard 2017; Dogan-Gangal 2024; Maul 2024
Chang and Chen 2005	Taiwan	RCT	Pregnant women scheduled for a cesarean section	Intervention (32): Music listening using a portable C play with headphones from beginning of anesthesia administration to end of cesarean section; participant choice of anxiety-relieving music including western classical, new-age, or Chinese religious songs<br>Control (32): Routine care	Anxiety	VAS-A	NR	Hunter 2023; Shafqat 2024; Weingarten 2021
Cheung 2018	China	RCT	Women undergoing fertility treatment	Intervention (66): Calming non-lyrical music through headphones during oocyte collection, approximately 60-80bpm, played at 60dB: once for 30m<br>Control (65): Headset without music	Anxiety<br>Pain<br>Depression<br>Patient satisfaction	STAI<br>VAS<br>BDI<br>Client Satisfaction Questionnaire	NR	Kizilkaya 2024
Choubsaz 2018	Iran	RCT	Pregnant women having a planned cesarean section	Intervention (30): Music listening during the entire cesarean section; pre-selected music by the researchers of sedative musical piece of Iranian Music Therapy Association<br>Control (60): Ear plugs or routine care	Anxiety	STAI	NR	Weingarten 2021
Dabas 2019	India	RCT	Mothers with newborns	Intervention (29): Passive listening to an audio-assisted relaxation technique on a laptop: once daily for 10 days (10 total sessions) for 30m<br>Control (28): Standard nursing care	Breastmilk volume	ml	NR	Duzgun 2020
Dehcheshmeh and Rafiei 2015	Iran	RCT	Primiparous pregnant women at full term having a vaginal birth	Intervention 1 (37): Music listening using headphones for 30 minutes: participant choice of piano music or wave sounds<br>Intervention 2 (37): Ice massage<br>Control (38): Routine care	Anxiety	VAS-A	NR	Hunter 2023; Sen 2023

Denney 2018	USA	RCT	Pregnant women scheduled for a cesarean section	Intervention (25): Music listening using a MP3 player while in the preoperative waiting area and postoperative recovery room; participant choice of a playlist with classical, pop/top 40, R&B, country, soft rock, or gospel music	Anxiety	STAI	NR	Hunter 2023; Weingarten 2021
				Control (25): Routine care				
Dereddy 2024	USA	RCT	Women in postpartum period	Intervention (20): Music therapy in the NICU with a music therapist: 4 times a day for 7 days (30mins): participant selection of preferred music	Anxiety Depression Stress	DASS-21	NR	Han 2024
				Control (20): Routine care				
Dolker 2019	Turkey	RCT	Pregnant women 32-41 wks gestation	Intervention (50): Music listening during non-stress test; selection of Turkish classical, folk, or classical music	Anxiety	STAI	NR	Ji 2024
				Control (50): Not specified				
Drzymalski 2023	US	RCT	Nulliparous women having an elective cesarean section	Intervention (10): Music listening via broadcast immediately before patient entry and throughout entire cesarean section; pre-selected Mozart sonatas	Anxiety	Maternal satisfaction scale for cesarean sections	NR	Shafqat 2024
				Control (10): Routine care				
Drzymalski 2020	US	RCT	Pregnant women having and elective cesarean section	Intervention 1 (49): Music listening before, during and after cesarean section; participant choice of preferred music on Pandora	Anxiety	Maternal satisfaction scale for cesarean sections	NR	Shafqat 2024
				Intervention 2 (50): Music listening before, during and after cesarean section; preselected Mozart sonatas				
				Control (50): Not specified				
Drzymalski 2017	US	RCT	Pregnant women scheduled for labor induction	Intervention (50): Music listening via broadcast during labor analgesia; participant choice of preferred music on Pandora	Anxiety Pain	Numeric rating scales	NR	Shafqat 2024
				Control (49): Not specified				
Ebneshahidi and Mohseni 2008		RCT	Pregnant women with planned cesarean sections	Intervention (38): Music listening with a soft open-air headphones and a tape player within 15mins of arrival to PACU; 1 session (30mins); participant choice of music	Anxiety Pain	VAS-A VAS-P	NR	Hakimi 2021; Weingarten 2021
				Control (39): Headphones with no music				

Eren 2018	Turkey	RCT	Pregnant women having a planned cesarean section	Intervention (30): Music listening during cesarean section; participant choice of music prior to operation<br>Control (30): Not specified	Anxiety<br>Vital signs	VAS<br>Systolic and diastolic blood pressure	NR	Hunter 2023; Shafqat 2024; Weingarten 2021
Estrella-Juarez 2023	New Zealand	RCT	Nulliparous pregnant women at full term (>37wks) having a vaginal, instrumental or cesarean section birth	Intervention 1 (109): Music listening via iPod with overhead headphones: 20 minute intervals during the first stage of labor; instrumental of music pre-selected by researcher<br>Intervention 2 (130): virtual reality during first stage of labor<br>Control (124): Routine care	Anxiety<br>Vital signs	STAI<br>Systolic and diastolic blood pressure	NR	Hunter 2023; Shafqat 2024
Fleury 2021	Brazil	RCT	Women undergoing fertility treatment	Intervention (50): Music therapy using small and easy to play percussive musical instruments, a guitar, voice, and a flute: once for 50 min<br>Control (50): No intervention	Anxiety<br>Stress	DASS 21<br>LSSI	NR	Kizilkaya 2024
Gaden 2022	Argentina, Colombia, Israel, Norway, and Poland	RCT	Postpartum women	Intervention (105): Music therapy with a music therapist in the NICU: 3 session per week (30mins) for a total of 27 sessions: participant selection of preferred music<br>Control (108): Routine care	Anxiety<br>Depression	GAD-7<br>EPDS	NR	Han 2024
Gan 2016	China	RCT	Pregnant with threatened abortion	Intervention 1 (60): Western medicine and medicinal diet and five-element music: Volume <75db, twice weekly for two weeks for 30 min<br>Intervention 2 (60): Western medicine and five-element music: Volume <75db, twice weekly for two weeks for 30m<br>Intervention 3 (60): Western medicine and medicinal diet<br>Control (60): Routine care (western medicine)	Anxiety<br>depression	Self-rating anxiety scale<br>Self-rating depression scale	Before, after 2 weeks	Wu 2020
Garcia González 2017	Spain	RCT	Nulliparous pregnant women: >28 wks gestation, medically low-risk	Intervention (204): Music listening of recorded music on a CD player: 3 sessions a week (40mins) with 14 sessions in total at home and during non-stress test; instrumental music preselected by researcher (60-75 beats/min)<br>Control (205): Not specified	Anxiety<br>Vital signs	STAI<br>Systolic and diastolic blood pressure	Before and after non-stress test	Dogan-Gangal 2024; Ji 2024; Hunter 2023; Maul 2024

Garcia-Gonzalez 2018	Spain	RCT	Pregnant women in 3rd trimester	Intervention (204): Music listening of recorded music on a CD player: 3 sessions a week (40mins) with 14 sessions in total at home and during non-stress test; instrumental music preselected by researcher (60-75 beats/min)	Anxiety	STAI	NR	Hunter 2023, Ji 2024, Lin 2019, Maul 2024, Shafqat 2024
				Control (205): Not specified				
Gokduman 2022	Turkey	RCT	Primiparous women who had vaginal delivery	Intervention (40): classical Turkish music with sterile virtual reality glasses	Pain	VAS-P	Immediately post-episiotomy and 1h later	Maleki 2023
				Control (44): Routine care				
Gönenç and Dikmen 2020	Turkey	RCT	Nulliparous pregnant women having a vaginal birth	Intervention 1 (33): Music listening using headphones once cervical dilation reached 4-5cm (active labor); 1 session (30mins); subjects choice of 3 songs with a range of upbeat pop music, slow pop music, Turkish folk music, and religious music	Pain, Pregnancy-related Anxiety	VAS<br>W-DEQA	NR	Hunter 2023, Sen 2023
				Intervention 2 (33): Dance and music listening				
				Control (33): Routine care				
Guerrero 2012	USA	RCT	Women undergoing an abortion	Intervention (54): Music listening during vacuum aspiration abortion procedure; participant choice of 10 preloaded playlists	Anxiety<br>Vital signs<br><br>Pain	STAI<br>Systolic and diastolic blood pressure, HR, RR<br>VAS	Before and after the procedure	Lin 2019
				Control (47): Routine care				
Guo 2022 [a]	China	RCT	Primiparous women having a vaginal birth	Intervention (201): Music therapist session to personalize music followed by music listening during all phases of labor: first phase of labor relaxing/hypnotic music was played, late stage of the first phase of labor: intense rhythmic music was played, second/third phase of labor parent–child music was played	Pain	Perception of labour pain questionnaire, perineal pain score	NR	Hunter 2023; Ji 2024, Sen 2023
				Control (239): standard labor practices with no music				
Halder 2022	India	RCT	Pregnant women scheduled for an elective cesarean section	Intervention (30): Music listening using headphones during the preoperative, intraoperative, and postoperative stages of the cesarean section; intervals of 20 mins; participant choice of preferred	Pain	VAS	NR	Hunter 2023

				genre or Indian classical, semi-classical, folk, light music, or instrumental<br>Control (30): Routine care				
Hanprasertpong 2016	Thailand	RCT	Pregnant women 15-21wks gestation who underwent a second trimester genetic amniocentesis	Intervention (161): Music listening using earphones throughout antiseptic skin preparation to the needle removal stage<br>Control (171): Not specified	Anxiety<br>Pain	VAS-A<br>VAS-P	NR	Shafqat 2024
Hepp 2018	Germany	RCT	Pregnant women having a cesarean section	Intervention (154): Music listening using CD player in operating theater; participant choice of classical, jazz, lounge, or meditation music with tempo at 60-80 bpm<br>Control (150): Routine care	Anxiety	VAS-A, STAI	NR	Hunter 2023; Ji 2024; Shafqat 2024; Weingarten 2021
Hinesley 2020		RCT	Pregnant women: 2nd-3rd trimester, medically low risk	Intervention (23): Composing a lullaby and listening teach recorded lullaby: 3 sessions (12hrs total); music created by subjects<br>Control (21): Not specified	Stress	Mental Health: SCL-27 and Pregnancy Stress Scale	NR	Dogan-Gangal 2024
Hoegholt 2024	De--ark	RCT	Pregnant nulliparous women: >25th week gestation	Intervention (31): Music listening and web-based advice on sleep hygiene: Daily sessions (30mins) for 28 days; participant selected music<br>Control (40): Web-based advice on sleep hygiene	Sleep quality	PSQI, Insomnia Severity Index.	NR	Hoffman 2025
Horasanli and Demirbas 2022	Turkey	RCT	Pregnant women with singletons over 37 wks gestation scheduled for elective cesarean section	Intervention (26): Music listening using an earpiece prior to administration of spinal anesthesia and throughout the entire cesarean section; pre-selection of sufi music with steady rhythm<br>Control (23): Routine care	Anxiety<br>Vital signs	STAI<br>Systolic and diastolic blood pressure, HR, RR, O2 sat	NR	Hunter 2023
Hosseini 2013	Iran	RCT	Pregnant women	Intervention (15): Music listening during labor; 2 sessions (30mins) within first two hours; pre-selected music by researchers: baraneeshgh (love rain) composed by Manouchehr cheshmazar<br>Control (15): Routine care	Anxiety<br>Pain	VAS<br>VAS-P Numerical pain rating scale, verbal pain rating scale	NR	Chuang 2018; Ji 2024;
Haung 2010	China	RCT	Women diagnosed with postpartum depression	Intervention (82): light music every other day for 6 weeks for 30m<br>Control (80): Psychological treatment, drug treatment	Depression	HAM-D	NR	Yang 2019

Kafali 2011	Turkey	RCT	Pregnant women: 36 wks gestation, medically low risk	Intervention (96): Music listening of recorded music during non-stress test at hospital: 1 session (30mins); participant's preference or selection among 3 types of music: classical, Turkish art, and Turkish folk music with 60-72 beats/min<br>Control (105): Not specified	Anxiety	STAI	Before and after non-stress test	Dogan-Gangal 2024; Lin 2019
Kakde 2023	Singapore	RCT	Pregnant women having an elective cesarean section	Intervention (53): Music listening using earphones during administration of spinal anesthesia and continuing during the cesarean section and post-surgery in the post-anesthesia care unit: 2 sessions (30mins)<br>Control (55): Routine care	Anxiety<br>Pain	VAS-A<br>Unspecified pain measurement tool	NR	Shafqat 2024
Karkal 2017	India	RCT	Primiparous women	Intervention (30): Music intervention<br>Control (30): Routine care	Anxiety<br>Pain	NR<br>NR	NR	Chuang 2018
Kaur 2023		RCT	Pregnant women having a cesarean section	Intervention (30): Music listening using headphones during cesarean section while under spinal anaesthesia; participant choice of folk, Hindi, film music, or religious songs<br>Control (30): Routine care with headphones worn	Anxiety	VAS-A; cortisol levels; hemodynamic parameters	NR	Hunter 2023
Kehl 2020	Switzerland	RCT	Postpartum women	Intervention (10): Music therapy with a music therapist in the NICU: 2-3 sessions per week (20mins) for a total of 8 sessions<br>Control (6): Routine care	Anxiety<br>Depression<br>Stress	STAI<br>EPS<br>PSS	NR	Han 2024
Kimber 2008	England	RCT	Pregnant women having a vaginal birth	Intervention 1 (30): Music with relaxation at the hospital; subjects were trained to focus on breathing and visualization techniques along with music listening during labor<br>Intervention 2 (30): Massage with relaxation at the hospital: subjects were trained to focus on breathing and visualization techniques along with massage during labor<br>Control (30): Routine care	Anxiety	VAS-A Cambridge birth worry scale, Labour agentry scale	NR	Hunter 2023; Chehreh 2023; Sen 2023
Kirca 2020	Turkey	RCT	Primiparous women who had vaginal delivery	Intervention (50): Turkish music (mother's preference) during episiotomy repair for 15-20m	Pain	VAS-P	Vaginal delivery, hymen	Maleki 2023

				Control (50): Routine care			repair, skin repair, immediately post-episiotomy repair and 1h later	
Kittithanesuan 2017	Thailand	RCT	Mothers with newborns	Intervention (152): Passive listening to regional music on a CD player: once for 11m	Breastmilk volume	ml	NR	Duzgun 2020
				Control (152): Standard nursing care				
Kobus 2022	Germany	RCT	Postpartum women	Intervention (40): Music therapy with a music therapist at the NICU: 2 sessions per week (10-50mins) until discharge	Depression	Allgemeine Depressions Skala, German version of the Center for Epidemiologic Studies Depression Scale	NR	Han 2024; Ji 2024
				Control (40): Routine care				
Küçükkaya 2024	Turkey	RCT	Women within 3h postpartum of full-term delivery	Intervention (41): Music listening at hospital of recorded Turkish music: 2 times a day for 36hrs (30 mins)	Depression	EPDS, Stein Blues Scale	Baseline, 12th hr post partum, 24th hr post partum, post-intervention	Han 2024; Sun 2024
				Control (41): Routine care				
Kumarilohar2018	India	NR	Pregnant women in labor	NR	Pain	VAS-P	NR	Chehreh 2023
Kurdi and Gasti 2018	India	RCT	Pregnant women having an emergency cesarean section	Intervention 1 (63): Music listening using a MP3 player and bilateral headphones covering entire ear during cesarean section; pre-selection of calming and soothing meditation music	Anxiety<br><br>Pain	VAS-A, psychological wellbeing questionnaire; VAS-P	NR	Hunter 2023; Weingarten 2021
				Intervention 2 (63): Music listening using a MP3 player and bilateral headphones covering entire ear during cesarean section; pre-selection of binaural beat meditation music				
				Control (63): Routine care while wearing headphones				
Kwun and Kim 2000	Korea	RCT	Pregnant women having planned cesarean section	Intervention (32): Music listening during preoperative period of cesarean section; 3 sessions (30mins); participant choice of music	Anxiety	STAI	NR	Weingarten 2021
				Control (32): Routine care				

Labrague 2013	Philippines	RCT	Pregnant women in latent labor	Intervention (25): Classical nature music through headphones and speakers for 30 min<br>Control (25): Not specified	Pain during labor	VAS	NR	Chehreh 2023, Sen 2023
Lee 2010	Korea	RCT	Women diagnosed with postpartum depression	Intervention (30): preferred music daily for 8 days for 40 min<br>Control (30): Routine care	Depression	BAI	NR	Yang 2019
Li J 2016	China	RCT	Pregnant with threatened abortion	Intervention (98): Five-element music (Gong (10 min), Shang (5 min), Jue (10 min), Zhi (5 min))<br>Control (98): Routine care	Anxiety<br>Depression	Self-rating anxiety scale<br>Self-rating depression scale	After intervention	Wu 2020
Li Z 2015	China	RCT	Postpartum	Intervention (40): electroacupuncture and five-element music<br>Control (42): Acupuncture	Depression	Self-rating depression scale, HAM-D	Before, after (3weeks, 6weeks)	Wu 2020
Li Z 2016a	China	RCT	Pregnant with threatened abortion	Intervention 1 (60): Western medicine and medicinal diet and five-element music: Volume <75db, twice weekly for two weeks for 30 min<br>Intervention 2 (60): Western medicine and medicinal diet<br>Intervention 3 (60): Western medicine and five-element music: Volume <75db, twice weekly for two weeks for 30m<br>Control (60): Routine care (western medicine)	Anxiety<br>Depression	HAM-A<br>HAM-D	Before, after 2 weeks	Wu 2020
Li Z 2016b	China	RCT	Pregnant with threatened abortion	Intervention (120): Five-element music: Volume <75db, twice weekly for two weeks for 30m<br>Control (120): Routine care	Anxiety<br>depression	Self-rating anxiety scale<br>Self-rating depression scale	Before, after 1,2 weeks	Wu 2020
Li and Dong 2012	China	RCT	Pregnant women scheduled for elective cesarean section	Intervention (30): Music listening for 30 minutes before and throughout the entire cesarean section; participant choice of classical Chinese music<br>Control (30): Routine care and 30 minutes of relaxation before cesarean section	Anxiety	Self-rating anxiety scale, heart rate variability, VAS	NR	Hunter 2023; Shafqat 2024; Weingarten 2021
Liu 2010	Taiwan	RCT	Primiparous women having a vaginal birth	Intervention (30): Music listening during the latent and active phases of labor for 30 minutes with or without headphones: participant choice of relaxing music, light music, popular music, crystal, children’s, or Chinese religious music.	Anxiety<br>Pain	VAS-A<br>VAS-P	During active and latent phases of labor	Chuang 2018; Hunter 2023; Shafqat 2024, Sen 2023

				Control (30): Routine care				
Liu 2014	China	RCT	Women diagnosed with postpartum depression	Intervention (41): Pure music every other day for 3 days for 1-2 hours<br>Control (41): Health education, psychological treatment	Depression<br>sleep	Self-rating depression scale<br>PSQI	NR	Yang 2019
Liu 2016	Taiwan	RCT	Sleep-disturbed pregnant women: 18-34 wks gestation	Intervention (61): Music listening of recorded music at bedtime: daily for 2 weeks (30mins); participant selected from their own collection or from 5 categories: lullaby, classical, nature sounds, crystal, or symphonic music with 60-80 beats/min<br>Control (60): Standard prenatal care	Anxiety<br>Sleep quality<br>Stress	STAI<br>PSQI<br>PSS	Baseline and post-intervention	Dogan-Gangal 2024; Ji 2024; Hoffman 2025; Lin 2019; Maul 2024 ; Shafqat 2024
Liu 2017	China	RCT	Pregnant women	Intervention (50): five-element music: twice daily for 8 weeks for 30m<br>Control (50): Routine care	Anxiety<br>depression	Self-rating anxiety scale<br>Self-rating depression scale	Before, after 6 weeks, 1 week after birth	Wu 2020
Mohd Shukri 2019	Malaysia	RCT	Mothers with newborns	Intervention (33): Passive listening to image-supported relaxing music: once daily for 2 weeks (14 total sessions)<br>Control (31): Standard nursing care	Breastmilk volume	ml	NR	Duzgun 2020
Momeni 2020	Iran	RCT	Pregnant women 37-42wks gestation	Intervention (65): Creating a calming enviro--ent: Snoezelen's room designed using an aquarium, and a projector, which played optical shapes, light music, and essential aroma<br>Control (65): Not specified	Anxiety	VAS-A	NR	Shafqat 2024
Moragianni 2009	USA	RCT	Women undergoing fertility treatment	Intervention (67): Harp music therapy with embryo transfer: once for 20m<br>Control (59): Standard care	Anxiety	STAI	NR	Kizilkaya 2024
Murphy 2014	USA	RCT	Women undergoing fertility treatment	Intervention (90): Harp music therapy with embryo transfer: once for 20m<br>Control (91): Standard care	Anxiety	STAI	NR	Kizilkaya 2024
Nandeibam 2022	India	RCT	Women undergoing fertility treatment	Intervention (54): Instrumental music (Zebronics, Zeb-thunder) through headphones for 30-45m<br>Control (55): Noise-cancelling headphones	Pain<br>Anxiety	VAS<br>Anxiety score	NR	Kizilkaya 2024
Nayak 2014	India	NR	Pregnant women in labor	NR	Pain	VAS-P	NR	Chehreh 2023
Nikandish 2007		RCT	NR	Intervention (50): Spanish guitar<br>Control (50): White music	Anxiety<br>Pain	VAS-A<br>VAS-P	NR	Hakimi 2021

Norouzi 2013	Iran	RCT	Women diagnosed with postpartum depression after cesarean section	Intervention (30): Lullaby daily for 30 min<br>Control (30): Kangaroo care	Anxiety	STAI	NR	Yang 2019
Nwebube 2017	UK	RCT	Pregnant women	Intervention (20): Music listening of traditional lullabies at home: Daily sessions (20mins) for 12 weeks<br>Control (16): Routine care	Anxiety<br>Depression	STAI<br>EPDS	Baseline and post-intervention	Han 2024; Lin 2019; Maul 2024; Shafqat 2024; Sun 2024
Orak 2020	Turkey	RCT	Women undergoing fertility treatment	Intervention 1 (31): Turkish classical music through headphones 1h before surgery: once for 60 min<br>Intervention 2 (31): Turkish classical music through headphones 1h before surgery and continuing through surgery: 60+ min<br>Control (31): No music	Pain	VAS	NR	Kizilkaya 2024
Palazzi 2021	Brazil	RCT	Postpartum women	Intervention (22): Music therapy with a music therapist at the NICU: 2 sessions per week (20-30 mins) for a total of 6 sessions; participant selection of preferred music<br>Control (17): Routine care	Anxiety<br>Depression<br>Stress	STAI<br>EPDS<br>PSS	NR	Han 2024
Parodi 2021	Italy	RCT	Low-risk pregnant women having an elective cesarean section	Intervention (40): Music listening of novel binaural beat technique audio track for deep relaxation during the preoperative period (within 1hr of cesarean section): 1 session (12 mins)<br>Control (20): Routine care	Anxiety	STAI	NR	Shafqat 2024; Weingarten 2021
Perkins 2023	UK	RCT	Women within 9 months postpartum	Intervention (44): Music therapy with a musically trained research assistants at home: 60-minute sessions for 6 weeks; frequency of sessions NR : participant selection of preferred music<br>Control (45): Routine care	Depression	EPDS, Social support and loneliness	Baseline, immediate post-intervention, 4 weeks post-intervention	Han 2024; Sun 2024
Perkovic 2021	Bosnia	RCT	Pregnant women in second and third trimesters	Intervention (90): Group education intervention with music listening: daily sessions (15min) before bedtime during pregnancy; participant choice of classical music<br>Control (85): Routine care	Anxiety<br><br>Pain	The symptom checklist—90<br>VAS-P	NR	Hunter 2023; Ji 2024
Phumdoung and Good 2003	Thailand	RCT	Primiparous women having a vaginal birth	Intervention (55): Music listening to soft music with earphones during first 3 hours of the active phase of labor:	Anxiety<br>Pain	VAS-A<br>VAS-P	During active phase of labor	Chuang 2018; Hunter 2023; Sen 2023

				participant choice of five types of Western music without lyrics: synthesizer, harp, piano, orchestra, and jazz.<br>Control (55): Routine care				
Qi 2023	China	RCT	Pregnant women 28-36wk gestation	Intervention (29): Music listening at home: daily sessions (20-30mins) for 4 weeks<br>Control (29): Routine care	Depression	EPDS	Baseline, immediate post-intervention, 6 weeks postpartum	Sun 2024
Rajakumari 2015	India	NR	Pregnant women in labor	NR	Pain	VAS-P	NR	Chehreh 2023
Reza 2007	Iran	RCT	Pregnant women having an elective cesarean section	Intervention 1 (50): Music listening using a CD player and headphones during cesarean section; pre-selection of Spanish-style guitar music<br>Control (50): white noise using CD player and headphones	Anxiety<br>Pain	VAS-A<br>VAS-P	NR	Shafqat 2024; Weingarten 2021
Rezaei 2023	Iran	RCT	Pregnant women 32-41wks gestation	Intervention 1(65): Inhalation of lavender aromatherapy drops and music listening using earphones before a non-stress test; pre-selected nature sounds<br>Intervention 2 (65): Inhalation of a placebo (distilled water) before a non-stress test<br>Control (65): No intervention	Anxiety	STAI	NR	Shafqat 2024
Ribeiro 2018	Brazil	RCT	Mothers of preterm infants admitted to NICUs	Intervention (10): Music sessions performed by professional music therapists at the hospital; weekly sessions (30-45 mins)<br>Control (11): Routine care	Depression	BDI	Baseline and discharge of infant	Sun 2024
Salafas 2022	Indonesia	Quasi-experimental	Pregnant women	Intervention (30): Music listening session (15mins); pre-selected relaxation music with 60 bpm<br>Control (0): None	Anxiety	HAM-A	NR	Ji 2024
Sanfilippo 2020	Gambia	RCT	Pregnant women	Intervention (50): Live music session including singing, moving to the music, and clapping led by medical staff at the clinic: weekly sessions (60 mins) for 6 weeks<br>Control (74): Routine care	Depression	EPDS	Baseline, immediate post-intervention, and 4wks post-intervention	Han 2024; Maul 2024

Sanli 2022	Turkey	RCT	Primiparous singleton pregnant women	Intervention (35): music listening and reminders: Sessions (30mins) 3 times a week for 2 weeks; musical pieces composed in uşşak mode selected by the researchers<br>Control (35): Not specified	Sleep quality	Adjusted PSQI	NR	Hoffman 2025; Ji 2024
Simavli 2014a	Turkey	RCT	Primiparous women who had vaginal delivery	Intervention (77): self-selected music (classical music, light music, popular music, Turkish art music, Turkish folk music and Turkish Sufi music) through headphones during labor for 2h periods with 20m breaks<br>Control (79): Routine care	Anxiety<br>Pain	STAI<br>FAS<br>VAS-A<br>VAS-P	Vaginal Delivery, 1, 4, 8, 16, 24h	Chuang 2018; Hunter 2023; Ji 2024; Maleki 2023 ; Sen 2024 ; Shafqat 2024
Simavli 2014b	Turkey	RCT	Primiparous women having a vaginal birth: 38+ wks gestation	Intervention (80*): Self-selected music listening at the hospital during labor: Non-stop music with 20min break every 2hrs until end of third phase of labor with or without headphones; participant choice of six types of music, including classical music, light music, popular music, Turkish art music, Turkish folk music, and Turkish Sufi music.<br>Control (81): Routine care	Anxiety<br>Depression<br>Pain	VAS-A<br>EPDS<br>VAS-P	During active and latent phases of labor, at vaginal delivery, 1h postpartum	Hakimi 2021; Han 2024 ; Hunter 2023; Ji 2024; Maleki 2023 ; Shafqat 2024; Sun 2024; Yang 2019
Sharifi 2013	Iran	RCT	Pregnant women having a cesarean section	Intervention (30): Music listening during preoperative period (within 2hrs of cesarean section): 1 session (20mins); pre-selected music by researchers including instrumental music or recitation of the Quran<br>Control (15): Routine care	Anxiety	STAI	NR	Weingarten 2021
Shin & Kim 2011	South Korea	CCT	Pregnant women: <14 weeks gestation, medically low risk	Intervention (117): Listening of recorded music for 1 session of 30mins during transvaginal ultrasound; music chosen by researchers (nature sounds)<br>Control (116): Transvaginal ultrasound without music	Anxiety<br>Stress	STAI<br>PSS	Baseline and immediately post intervention	Corbijn van Willenswaard 2017; Dogan-Gangal 2024
Shobeiri 2016	Iran	RCT	Singleton pregnant women: 30-34th week gestation with sleep disorder	Intervention (42): Music listening and two weekly sessions of music therapy counseling in groups: Daily sessions (45mins) for 28 days; music selected by the researchers<br>Control (44): Not specified	Sleep quality	PSQI	NR	Hoffman 2025

Soylu 2022	Turkey	RCT	Pregnant women	Intervention (37): Music listening using a MP3 player and earphones during a nonstress test; 1 session (30mins); participant selection of preferred music<br>Control (37): Not specified	Anxiety	STAI	NR	Shafqat 2024
Stocker 2016	UK	RCT	Women undergoing fertility treatment	Intervention (21): Music of choice through headphones: once for 15m<br>Control (21): No music	Anxiety	STAI	NR	Kizilkaya 2024
Su 2014	China	RCT	Women diagnosed with postpartum depression	Intervention (80): pure music twice daily for 6 weeks for 30m<br>Control (80): Psychological treatment, traditional Chinese medicine	Depression	NR	NR	Yang 2019
Surucu 2018	Turkey	RCT	Healthy pregnant primiparous women having a vaginal birth	Intervention (25): Music listening in active labor (4cm) through headphones for 3 hours, alternating between 20 mins of listening and 10 mins break: acemasiran music selected for the participant<br>Control (25): Routine care	Anxiety<br>Pain	VAS-A, STAI, FAS (face anxiety scale)<br>Pain perception	NR	Hunter 2023; Ji 2024; Shafqat 2024, Sen 2023
Suryani 2021	Indonesia	RCT	Nulliparous pregnant women in active labor	Intervention (30): Instrumental music<br>Control (30): Not specified	Pain<br>Anxiety	VAS<br>STAI	NR	Sen 2023
Taghinejad 2010	Iran	RCT	Primiparous pregnant women with singleton pregnancies having a vaginal birth	Intervention 1 (50): Music listening with headphones during early active phase of labor: 1 session (30mins); participant choice of five types of soft traditional music without lyrics<br>Intervention 2 (51): Massage during early active phase of labor	Anxiety	VAS-A	NR	Hunter 2023; Chehreh 2023
Teckenberg-Jansson 2019	Finland	RCT	Women hospitalized with pregnancy-related complication	Intervention (52): Music therapy with music therapist playing two lyre instruments and humming at hospital bedside; Daily sessions (30mins) for 3 days<br>Control (50): Routine care	Anxiety<br>Stress	STAI<br>PSS	Baseline and post-intervention	Lin 2019; Maul 2024; Shafqat 2024
Toker and Komurcu 2017	Turkey	RCT	Pregnant women with preeclampsia (≥30 wks gestation)	Intervention (35): Music listening using MP3 player and headphones and deep breathing at hospital: daily sessions (30mins) for 5 days before and 2 days after labor; participant selected from 2 categories: Nihavend or Buselik modes<br>Control (35): Not specified	Anxiety<br>Vital signs	STAI<br>Systolic and diastolic blood pressure	Baseline and 5th day of the intervention	Dogan-Gangal 2024; Lin 2019; Maul 2024; Shafqat 2024
Tseng 2010		RCT	NR	Intervention (37): Music therapy	Anxiety	STAI	NR	Hakimi 2021

				Control (40): Usual care	Stress	PSS		
Ventura 2012	Portugal	Quasi-experimental	Pregnant women having a singleton pregnancy, low risk, waiting for amniocentesis	Intervention (154): Music listening using CD player while sitting and reading magazines; sessions 30 mins; participant choice of 4 types of music	Anxiety	STAI	NR	Shafqat 2024
				Control (0): None				
Vianna 2011	Brazil	RCT	Mothers with newborns	Intervention (48): Passive and active listening (playing instruments and singing): once daily for 3 days (3 total sessions) for 60 min	Breastfeeding rate	NR	NR	Duzgun 2020
				Control (48): Standard nursing care				
Wan and Wen 2018	China	RCT	Pregnant women having a vaginal birth	Intervention 1 (60): Music listening during labor and delivery: 20 min sessions followed by a 2-hour break	Anxiety<br>Pain	VAS-A<br>VAS-P	NR	Hunter 2023
				Intervention 2 (60): Acupressure during labor and delivery				
				Intervention 3 (62): Music listening and acupressure during labor and delivery				
				Control (59): Routine care				
Wang 2016a	China	RCT	Postpartum	Intervention (200): Five-element music: 40db, twice daily for 3 days for 30 min	Anxiety<br><br>Depression	Self-rating anxiety scale<br>Self-rating depression scale	24 and 72 h post-birth	Wu 2020
				Control (200): Routine care				
Wang 2016b	China	RCT	Women diagnosed with postpartum depression after cesarean section	Intervention 1 (200): Pure music every other day for 3 days for 30 min	Anxiety<br><br>Depression	Self-rating anxiety scale<br>Self-rating depression scale	NR	Yang 2019
				Intervention 2 (200): Psychological treatment				
				Control (52): Kangaroo care				
Wang 2018	China	RCT	Postpartum	Intervention (60): Five-element music: 20-40db, once daily for 8 weeks for 30-60 mIN	Depression	HAM-D<br>EDPS	Before, after 8weeks	Wu 2020
				Control (60): routine care				
Wang 2019	China	RCT	Pregnant in labor	Intervention 1 (53): Electroacupuncture	Pain<br>Length of labor<br>Blood loss	VAS<br>Mins<br><br>ml<br>Serum dynomorphin	Before, after	Wu 2020
				Intervention 2 (55): Electroacupuncture and five-element music: 30m every 2h throughout labor				
				Intervention 3 (54): Medicine				
				Control (51): No intervention				
Wei 2013	China	RCT	Postpartum	Intervention (60): Five-element music: daily for 8 weeks for 30m	Depression	EPDS	Before, after (2, 4, 6, 8 weeks)	Wu 2020
				Control (60): Medicine				

Wu 2012	USA	RCT	Women undergoing an elective abortion	Intervention (13): Music listening during surgical abortion procedure; participant choice of 5 preloaded playlists	Anxiety<br>Pain	11-point verbal numerical scales of anxiety and pain, STAI	Baseline, prior to pelvic exam, during surgical procedure, after speculum removal, and 30mins after the procedure;	Lin 2019; Shafqat 2024
				Control (13): Routine care				
Wulff 2021a	Germany	RCT	Pregnant women: 24-36wks gestation	Intervention 1 (64): A 30-min group music session was led by a music therapist with up to 3 other women between 30 and 34th wk gestation to practice relaxation through passive music listening and instruction on how to listen and relax to music at home; subjects received a CD with classical, calm music without lyrics and a soothing calm beat to listen to at home for 10-15 mins a day until birth, subjects were also free to choose and listen to other music	Anxiety<br>Depression<br>Emotional state<br>Maternal attachment	STAI<br>EPDS<br>VAS<br><br>Maternal Antenatal Attachment Scale	30th and 36th gestational week in intervention groups (before and after intervention)	Han 2024; Maul 2024
				Intervention 2 (59): Two 30-minute group singing sessions were led by a music therapist with up to 7 other women between 30th and 34th wk gestation; music therapist practiced children's songs and lullabies with the group and played the guitar; subjects received a song book with lyrics and melodies of 10 children's songs and lullabies and asked to continue sessions on a daily basis at home for 10-15 mins a day until birth				
				Control (49): Routine care				
Wulff 2021b	Germany	RCT	Postpartum women	Intervention (59): Up to three 45-minute music sessions led by a music therapist with 5-10 women and their babies at the hospital between 3 and 12 weeks postpartum, including elements of finger games, lullabies and movements to music; Subjects asked to complete	Anxiety<br>Depression<br>Emotional state<br>Maternal attachment	STAI<br>EPDS<br>VAS<br><br>Postpartum bonding questionnaire	Baseline (within 48hrs of delivery), 2 weeks postpartum, 12 weeks postpartum	Sun 2024

				singing and music-based interaction between mother and baby daily at home; standard repertoire of songs and games were provided along with subjects' requests				
				Control (61): Routine care				
Xavier 2016	India	NR	Pregnant women in labor	NR	Pain	VAS-P	NR	Chehreh 2023
Xu 2017	China	RCT	Pregnant in labor	Intervention (50): five-element music: volume 40 db, 30 min every 2h for duration of labor	Anxiety	Self-rating anxiety scale	Before, 24h after birth	Wu 2020
				Control (50): Routine care				
Yang 2009	China	RCT	Pregnant women: 28-36 wks gestation, medically high risk	Intervention (60): Usual care plus listening to music for 3 days for 30minutes/day in hospital; participant selected among 3 types of music: classical music, pleasant music, and Chinese folk music with 60-72 beats/min	Anxiety<br>Vital signs	STAI<br>Systolic and diastolic blood pressure, heart rate, respiratory rate	Before and 2hrs after final session	Corbijn van Willenswaard 2017; Dogan-Gangal 2024; Lin 2019; Maul 2024
				Control (60): Usual care				
Yüksekol 2020	Turkey	RCT	Pregnant women 28-32wks gestation hospitalized with preeclampsia	Intervention (30): Music listening at the hospital: 2 sessions (30mins) a day (morning and evening)	Anxiety<br>Vital signs	STAI<br>Systolic and diastolic blood pressure	NR	Shafqat 2024
				Control (30): No music				
Zou 2018	China	RCT	Postpartum	Intervention 1 (62): acupressure and five-element music: 40-60db, twice daily for 3 days for 30 min	Anxiety<br><br>Milk production	Self-rating anxiety scale<br>Milk yield<br>Breast fullness	Before, 72 h post-birth	Wu 2020
				Intervention 2 (62): Acupressure				
				Control (62): Five-element music: 40-60 db, twice daily for 3 days for 30 min				

Notes:
[a] Guo 2022 has been retracted. We will not present any analyses with these data.
