# Supplementary material for: Music interventions to improve women’s health outcomes in the preconception, antepartum, intrapartum, and postpartum periods: An overview of reviews
Source: PLoS One. 2026 Feb 18;21(2):e0339337. doi: 10.1371/journal.pone.0339337 (PMC12915951; doi:10.1371/journal.pone.0339337)
Supplement: S6 Table — (PDF) [file pone.0339337.s006.pdf]

Supplementary Materials

Table S6: Methodological Quality of Systematic Reviews Using the AMSTAR 2 Tool (N=20)

Review	Criteria [a]																Overall confidence [b]
	1	2	3	4	5	6	7	8	9	10	11	12	13	14	15	16	
Kızılkaya 2024	+	+	+	-	+	+	+	+	+	+	+	+	+	+	X	+	Low
Hoffmann 2025	+	+	+	+	+	+	X	+	+	X	X	X	+	+	+	+	Critically low
Dogan-Gangal 2024	+	+	+	X	+	+	X	+	-	X	--	--	+	+	--	+	Critically low
Maul 2024	+	+	+	-	+	X	+	+	+	X	--	--	+	+	--	+	Moderate
Lin 2019	+	X	+	-	+	+	X	+	+	X	+	+	X	+	+	+	Critically low
Corbijn van Willenswaard 2017	+	X	X	-	+	+	X	+	-	X	+	+	+	+	X	+	Critically low
Chehreh 2023	+	X	+	-	+	+	X	X	X	X	+	X	X	X	X	+	Critically low
Hunter 2023	+	X	+	-	+	X	X	+	+	+	--	--	+	+	--	+	Critically low
Şen 2023	+	+	X	X	+	+	X	+	+	X	+	+	+	+	X	+	Critically low
Chuang 2019	X	X	+	-	+	+	X	X	+	X	+	+	+	+	+	+	Critically low
Maleki and Youseflu 2023	+	X	X	-	+	+	X	+	+	+	+	+	X	+	+	+	Critically low
Weingarten 2021	+	+	X	-	+	+	+	+	+	+	+	+	+	+	X	+	Low
Hakimi 2021	+	X	X	X	+	+	X	-	+	X	X	X	+	X	X	+	Critically low
Düzgün and Özer 2020	+	+	+	-	+	+	X	+	+	+	+	+	+	+	+	+	Low
Yang 2019	+	+	+	-	+	+	X	-	+	X	+	+	+	+	X	+	Critically low
Han 2024	+	-	+	X	+	+	X	+	+	X	--	--	X	+	--	+	Critically low
Ji 2024	+	X	X	X	+	+	X	-	X	X	--	--	X	+	--	+	Critically low
Shafqat 2024	+	-	X	X	+	+	X	+	+	X	--	--	+	+	--	+	Critically low
Sun 2024	+	+	+	X	+	+	+	+	+	X	+	+	X	+	+	+	Critically low
Wu 2020	+	X	X	-	+	+	X	-	+	X	+	+	+	X	+	X	Critically low

Key: +: Yes; -: Partial Yes; X: No; --: No meta-analysis conducted

[a] Criteria included the following questions: [1]Did the research questions and inclusion criteria for the review include the components of PICO? [2]Did the report of the review contain an explicit statement that the review methods were established prior to the conduct of the review and did the report justify any significant deviations from the protocol? [3]Did the review authors explain their selection of the study designs for inclusion in the review? [4]Did the review authors use a comprehensive literature search strategy? [5]Did the review authors perform study selection in duplicate? [6]Did the review authors perform data extraction in duplicate? [7]Did the review authors provide a list of excluded studies and justify the exclusions? [8]Did the review authors describe the included studies in adequate detail? [9]Did the review authors use a satisfactory technique for assessing the risk of bias (RoB) in individual studies that were included in the review? [10]Did the review authors report on the sources of funding for the studies included in the review? [11]If meta-analysis was performed did the review authors use appropriate methods for statistical combination of results? [12]If meta-analysis was performed, did the review authors assess the potential impact of RoB in individual studies on the results

of the meta-analysis or other evidence synthesis? [13]Did the review authors account for RoB in individual studies when interpreting/discussing the results of the review? [14]Did the review authors provide a satisfactory explanation for, and discussion of, any heterogeneity observed in the results of the review? [15]If they performed quantitative synthesis did the review authors carry out an adequate investigation of publication bias (small study bias) and discuss its likely impact on the results of the review? [16]Did the review authors report any potential sources of conflict of interest, including any funding they received for conducting the review?
[b] Guidance has determined that criteria 2, 4, 7, 9, 11, 13 and 15 are critical. Overall confidence is assessed using the following guidance: High (0-1 non-critical weakness), Moderate (>1 non-critical weakness), Low (1 critical flaw with or without non-critical weaknesses), and Critically low (>1 critical flaw with or without non-critical weaknesses).
