# Supplementary material for: Music interventions to improve women’s health outcomes in the preconception, antepartum, intrapartum, and postpartum periods: An overview of reviews
Source: PLoS One. 2026 Feb 18;21(2):e0339337. doi: 10.1371/journal.pone.0339337 (PMC12915951; doi:10.1371/journal.pone.0339337)
Supplement: S7 Table — (PDF) [file pone.0339337.s007.pdf]

Supplementary Materials

Table S7: Primary Study Risk of Bias Assessments (RoB-1)

**Primary study (1st Author Year)**	**Review (1st Author Year)**	**Random sequence generation (selection bias)**	**Allocation concealment (selection bias)**	**Blinding of subjects and personnel (performance bias)**	**Blinding of outcome assessment (detection bias)**	**Incomplete outcome data (attrition bias)**	**Selective reporting (reporting bias)**	**Other bias**
Aba 2017	Kizilkaya 2024	+	+	X	X	-	-	+
Abarghoee 2022	Hunter 2023 [a]	+	+	X	X *	X *	- *	- *
Abhari 2000	Chehreh 2023 [b]	n/a	n/a	n/a	n/a	n/a	n/a	n/a
AjorPaz and Ranjbar 2010	Weingarten 2021	+	+	-	-	+	+	+
Ak 2015	Duzgun 2020 [c]	-	n/a	n/a	+	+	+	n/a
Akmese and Oran 2014	Dogan-Gangal 2024 [d]	+	+	+	+	X	+ *	+ *
Allameh 2013	Weingarten 2021	-	-	-	-	-	+	+
Almedhesh 2022	Shafqat 2024 [e]	+	+	-	+	+	+ *	+ *
Anamak 2020	Hunter 2023 [a]; Sen 2023	+	+	X	+	+	+	-
Angin 2020	Maleki 2023	X	X	X	X	+	+	+
Baltaci 2022	Shafqat 2024 [e]	+	+	X	-	+	+ *	+ *
Baltaci 2024	Maul 2024	+	+ *	+ *	-	+	-	+ *
Bansal 2019	Wu 2020	-	-	-	-	+	-	-
Bauer 2010	Corbijn van Willenswaard 2017; Dogan-Gangal 2024[d]	+	+	-	-	-	-	-
Browning 2001	Hunter 2023 [a]	+	-	X	- *	+ *	- *	- *
Buglione 2020	Hunter 2023 [a]; Maleki 2023; Sen 2023; Ji 2024 [f]; Shafqat 2024 [e]	+	+	X	X	+	+	+
Cao 2016	Lin 2019; Maul 2024; Shafqat 2024 [e]; Sun 2024	+	-	-	-	+	-	-
Cappon 2014	Dogan-Gangal 2024 [d]	+	+	+	+	-	- *	X *
Catalgol 2021	Ji 2024[f]; Maul 2024; Shafqat 2024 [e]	+	+	+	+	+	-	-
Chaichanalap 2018	Maleki 2023	+	+	+	X	+	+	+
Chang 2008	Corbijn van Willenswaard 2017; Dogan-Gangal 2024 [d]; Han 2024; Lin 2019; Maul 2024; Shafqat 2024 [e]; Sun 2024	+	-	-	-	+	-	+

**Primary study (1st Author Year)**	**Review (1st Author Year)**	**Random sequence generation (selection bias)**	**Allocation concealment (selection bias)**	**Blinding of subjects and personnel (performance bias)**	**Blinding of outcome assessment (detection bias)**	**Incomplete outcome data (attrition bias)**	**Selective reporting (reporting bias)**	**Other bias**
Chang 2015	Corbijn van Willenswaard 2017; Dogan-Gangal 2024 [d]; Maul 2024	-	-	-	-	-	-	-
Chang and Chen 2005	Weingarten 2021; Hunter 2023 [a]; Shafqat 2024 [e]	+	-	X	-	+	-	+
Cheung 2018	Kizilkaya 2024	+	-	X	X	+	-	+
Choubsaz 2018	Weingarten 2021	-	-	X	-	+	+	+
Dabas 2019	Duzgun 2020	+	+ *	X *	+	+	+	+ *
Dehcheshmeh and Rafiei 2015	Hunter 2023 [a]; Sen 2023	+	-	X	+	+	+	-
Denney 2018	Weingarten 2021; Hunter 2023 [a]	+	+	X	-	+	+	+
Dereddy 2024	Han 2024	+	+	X	-	+	+	+
Dolker 2019	Ji 2024 [f]	X *	X *	X *	X *	+ *	- *	+ *
Drzymalski 2017	Shafqat 2024 [e]	+	+	-	-	+	+ *	+ *
Drzymalski 2020	Shafqat 2024 [e]	+	+	-	-	+	X *	+ *
Drzymalski 2023	Shafqat 2024 [e]	+	+	X	-	+	+ *	+ *
Ebneshahidi and Mohseni 2008	Hakimi 2021; Weingarten 2021	-	-	-	+	+	+	+
Eren 2018	Weingarten 2021; Hunter 2023 [a]; Shafqat 2024 [e]	-	-	X	-	+	+ *	+ *
Estrella-Juarez 2023	Hunter 2023 [a]; Shafqat 2024 [e]	+	+	-	+	+	- *	+ *
Fleury 2021	Kizilkaya 2024	+	-	X	X	-	-	+
Gaden 2022	Han 2024	+	+	X	+	+	+	+
Gan 2016	Wu 2020	-	-	-	-	+	X	-
Garcia González 2018a	Dogan-Gangal 2024[d]; Hunter 2023[a]	+	+	-	-	+	- *	+ *
Garcia González 2018b	Ji 2024 [f]; Maul 2024	+	+ *	- *	-	+	-	- *
Garcia González 2018c	Lin 2019; Ji 2024; Maul 2024; Shafqat 2024[e];	+	+	-	-	+	-	-
Gokduman 2022	Maleki 2023	+	+	X	X	+	+	+
Gönenç and Dikmen 2020	Hunter 2023 [a]; Sen 2023	+	+	X	+	+	+	-
Guerrero 2019	Lin 2019	-	+ *	X *	X *	+	+	+ *

Primary study (1st Author Year)	Review (1st Author Year)	Random sequence generation (selection bias)	Allocation concealment (selection bias)	Blinding of subjects and personnel (performance bias)	Blinding of outcome assessment (detection bias)	Incomplete outcome data (attrition bias)	Selective reporting (reporting bias)	Other bias
Guo 2022[g]	Hunter 2023[a]; Sen 2023; Ji 2024	+	-	-	+	+	+	-
Halder 2022	Hunter 2023[a]	+	+	X	X *	+ *	- *	+ *
Hanprasertpong 2016	Shafqat 2024[e]	+	+	+	+	+	- *	- *
Haung 2010	Yang 2019	+	+	+	+	+	-	+
Hepp 2018	Weingarten 2021; Hunter 2023[a]; Ji 2024[f]; Shafqat 2024[e]	+	+	X	-	+	+	+
Hinesley 2020	Dogan-Gangal 2024[d]	+	+	+	+	X	- *	X *
Hoegholt 2024	Hoffman 2025	+	+	X	+	X	+	+
Horasanli and Demirbas 2022	Hunter 2023[a]	+	X	X	X *	- *	- *	+ *
Hosseini 2013	Chuang 2018; Ji 2024[f]	X	X	X	X	+	+	-
Kafali 2011	Lin 2019; Dogan-Gangal 2024[d]	-	+	+	+	-	-	-
Kakde 2023	Shafqat 2024[e]	+	+	-	-	+	- *	- *
Karkal 2017	Chuang 2018	-	+	X	+	+	+	-
Kaur 2023	Hunter 2023[a]	+	+	X	+ *	- *	- *	+ *
Kehl 2020	Han 2024	-	-	X	+	+	X	+
Khoshkholgh 2016	Dogan-Gangal 2024[d]	+	+	-	-	X	- *	+ *
Kimber 2008	Chehreh 2023; Hunter 2023[a]; Sen 2023	+	+	-	+	+	+	-
Kittithanesuan 2017	Duzgun 2020	+	+ *	- *	+	+	+	+ *
Kirca 2020	Maleki 2023	+	+	+	+	+	+	+
Kobus 2022	Han 2024; Ji 2024[f]	-	-	-	-	+	+	+
Küçükkaya 2024	Han 2024; Sun 2024	-	-	X	-	+	+	+
Kumarilohar 2018	Chehreh 2023	- *	- *	X *	- *	- *	- *	- *
Kurdi and Gasti	Weingarten 2021; Hunter 2023[a]	+	+	X	-	+	+	+
Kwun and Kim 2000	Weingarten 2021	-	-	-	+	+	+	+
Labrague 2013	Chehreh 2023; Sen 2023	+	- *	- *	+	+	+	- *

Primary study (1st Author Year)	Review (1st Author Year)	Random sequence generation (selection bias)	Allocation concealment (selection bias)	Blinding of subjects and personnel (performance bias)	Blinding of outcome assessment (detection bias)	Incomplete outcome data (attrition bias)	Selective reporting (reporting bias)	Other bias
Lee 2010	Yang 2019	−	−	−	−	+	+	+
Li and Dong 2012	Weingarten 2021; Hunter 2023 [a]; Shafqat 2024 [e]	+	+	−	−	+	+	+
Li Z 2015	Wu 2020	+	−	−	−	+	−	−
Li Z 2016a	Wu 2020	+	−	−	−	+	−	−
Li Z 2016b	Wu 2020	−	−	−	−	+	X	−
Li P 2015	Wu 2020	X	−	−	−	+	−	−
Li J 2016	Wu 2020	−	−	−	−	+	−	−
Liu 2010	Chuang 2018; Hunter 2023 [a]; Sen 2023; Shafqat 2024 [e]	+	−	X	+	+	+	−
Liu 2014	Yang 2019	+	+	+	+	+	−	+
Liu 2016	Lin 2019; Dogan-Gangal 2024 [d]; Ji 2024 [f]; Maul 2024; Shafqat 2024 [e]; Hoffmann 2025	+	−	−	−	+	−	+
Liu 2017	Wu 2020	+	−	−	−	X	−	−
Mohd Shukri 2019	Duzgun 2020	+	+ *	+ *	+	+	+	+ *
Momeni 2020	Shafqat 2024 [e]	X	+	X	−	+	− *	X *
Moragianni 2009	Kizilkaya 2024	+	+	−	−	+	−	+
Murphy 2014	Kizilkaya 2024	+	+	X	X	−	−	+
Nandeibam 2022	Kizilkaya 2024	+	+	X	−	+	−	+
Nayak 2014	Chehreh 2023	X *	X *	X *	X *	− *	− *	− *
Nikandish 2007	Hakimi 2021	+	−	+	+	+	+	−
Norouzi 2013	Yang 2019	+	−	−	−	+	+	+
Nwebube 2017	Lin 2019; Han 2024; Maul 2024; Shafqat 2024 [e]; Sun 2024	+	+	−	−	−	+	+
Orak 2020	Kizilkaya 2024	+	+	X	X	+	−	+
Palazzi 2021	Han 2024	X	−	X	−	+	+	+
Parodi 2021	Weingarten 2021; Shafqat 2024 [e]	+	+	+	+	−	+	+
Perkins 2023	Han 2024; Sun 2024	−	−	−	−	−	+	+

Primary study (1st Author Year)	Review (1st Author Year)	Random sequence generation (selection bias)	Allocation concealment (selection bias)	Blinding of subjects and personnel (performance bias)	Blinding of outcome assessment (detection bias)	Incomplete outcome data (attrition bias)	Selective reporting (reporting bias)	Other bias
Perkovic 2021	Hunter 2023 [a]; Ji 2024 [f]	+	−	−	− *	X *	− *	+ *
Phumdoung and Good 2003	Chuang 2018; Hunter 2023[a]; Sen 2023	+	−	−	−	+	+	−
Qi 2023	Sun 2024	−	+ *	X *	+	+	+	+ *
Rajakumari 2015	Weingarten 2021; Chehreh 2023; Shafqat 2024 [e]	+	+	+	+	+	+	+
Rezaei 2023	Shafqat 2024 [e]	+	−	−	−	+	− *	+ *
Ribeiro	Weingarten 2021	−	−	X	−	+	+	+
Salafas 2022	Ji 2024 [f]	X *	X *	X *	X *	+ *	− *	− *
Sanfilippo 2020	Han 2024; Maul 2024	−	+	X	−	+	−	+
Sanli 2022	Ji 2024 [f]; Hoffman 2025	+	−	X	−	+	+	+
Serap 2014	Yang 2019	+	−	−	−	+	+	+
Sharifi 2013	Weingarten 2021	−	−	−	−	+	+	+
Shin & Kim 2011	Corbijn van Willenswaard 2017; Dogan-Gangal 2024 [d]	−	−	X	X	−	−	−
Shobeiri 2016	Hoffman 2025	−	−	X	−	+	+	X
Simavli 2014a	Chuang 2019; Hakimi 2021; Hunter 2023 [a]; Maleki 2023; Sen 2023; Ji 2024 [f]; Shafqat 2024 [e]	+	+	X	+	+	+	−
Simalvi 2014b	Hunter 2023 [a]; Maleki 2023; Han 2024; Shafqat 2024 [e]	+	+	+	+	+	+	+
Soylu 2022	Shafqat 2024 [e]	+	X	X	−	+	− *	+ *
Stocker 2016	Kizilkaya 2024	+	+	X	X	+	−	+
Su 2014	Yang 2019	X	−	−	−	+	+	+
Surucu 2018	Hunter 2023 [a]; Sen 2023; Ji 2024 [f]; Shafqat 2024 [e]	+	−	−	−	+	+	−
Suryani 2021	Sen 2023	+	− *	X *	+	+	+	− *
Taghinejad 2010	Chehreh 2023; Hunter 2023 [a]	+	+	−	+ *	+ *	− *	+ *
Teckenberg-Jansson 2019	Lin 2019; Maul 2024; Shafqat 2024 [e]	+	+	−	−	+	−	−
Toker and Komurcu 2017	Lin 2019; Dogan-Gangal 2024 [d]; Maul 2024; Shafqat 2024 [e]	+	+	−	−	+	−	−
Tseng 2010	Hakimi 2021	X	+	X	X	+	+	−

Primary study (1st Author Year)	Review (1st Author Year)	Random sequence generation (selection bias)	Allocation concealment (selection bias)	Blinding of subjects and personnel (performance bias)	Blinding of outcome assessment (detection bias)	Incomplete outcome data (attrition bias)	Selective reporting (reporting bias)	Other bias
Ventura 2012	Shafqat 2024 [e]	+	+	-	-	+	-*	-*
Vianna 2011	Duzgun 2020	+	+*	X*	+	+	+	+
Xavier 2016	Chehreh 2023	X*	X*	X*	-*	+*	-*	-*
Xu 2017	Wu 2020	+	-	-	-	+	-	-
Wan and Wen 2018	Hunter 2023 [a]	+	-	+	+*	+*	-*	+*
Wang 2016a	Wu 2020	+	-	-	-	+	-	-
Wang 2018	Wu 2020	+	-	-	-	+	-	-
Wang 2019	Wu 2020	-	-	-	-	+	-	-
Wei 2013	Yang 2019	+	-	-	-	+	+	+
Wu 2012	Lin 2019; Shafqat 2024 [e]	-	+	X	+	+	-	-
Wulff 2021a	Han 2024; Maul 2024	-	-	X	-	+	+	+
Wulff 2021b	Han 2024; Maul 2024	-	X	X	-	-	+	+
Yang 2009	Corbijn van Willenswaard 2017; Lin 2019; Dogan-Gangal 2024 [d]; Maul 2024	+	-	-	+	-	-	-
Yüksekol 2020	Shafqat 2024 [e]	+	+	-	-	+	-*	+*
Zou 2018	Wu 2020	-	-	-	-	+	-	+

Key: + : Low risk; - : Some concerns; X : High risk

Notes: If more than one review assessed the risk of bias of an article and the assessments were different, we kept the most common rating. Unclear rating was employed if there was no consensus in ratings across reviews that rated the same primary article.

*Denotes rating conducted by MM or BK in the absence of an assessment by a review.

[a] The Scottish Intercollegiate Guidelines Network (SIGN) ratings were mapped to the RoB criteria.

[b] Risk of bias was not assessed by Chehreh 2023 and we cannot locate primary study article.

[c] We cannot locate this primary study article; it is also not listed in Duzgun 2020 reference list.

[d] Quality Assessment Tool for Quantitative Studies (QATQS) ratings were mapped to the RoB criteria.

[e] JBI Critical Appraisal Tool was mapped to the RoB assessment.

[f] Ji 2024 did not conduct any risk of bias assessment

[g] Guo 2022 has been retracted
