# Supplementary material for: Music interventions to improve women’s health outcomes in the preconception, antepartum, intrapartum, and postpartum periods: An overview of reviews
Source: PLoS One. 2026 Feb 18;21(2):e0339337. doi: 10.1371/journal.pone.0339337 (PMC12915951; doi:10.1371/journal.pone.0339337)
Supplement: S8 Table — (PDF) [file pone.0339337.s008.pdf]

Supplementary Materials

Table S8: Summary of Effects of Music Interventions on Anxiety

Review	Comparison	Outcome measurement	No. of subjects (trials)	Effect (95% CI)	I^2 (%)	Quality of evidence (GRADE)	Comments	Primary studies
Preconception interventions								
Kızılkaya 2024(1)	Music listening and music therapy vs. no intervention	Anxiety: Composite measure derived from: DASS 21, STAI, VAS-A	902 (8)	SMD= -0.27 (-0.40, -0.14)	44%	Low	Serious bias: all studies had unclear or high risk related to blinding; Serious indirectness: variability in interventions and outcome measures	(2–9)
Antepartum interventions								
Lin 2019(10)	Music listening and music therapy vs. no intervention	Anxiety: STAI	1234 (8)	SMD: -0.42 (-0.83, -0.02)	91%	Very low	Very serious bias: All studies had overall high risk; Very serious inconsistency: high I^2; Serious indirectness: variability in intervention	(11–18)
Corbijn van Willenswaard 2017	Music listening vs. no intervention	Anxiety: STAI	469 (2)	SMD: -0.21 (-0.39, -0.03)	0%	Low	Serious bias: High risk in sequence generation, allocation concealment and blinding in 1 of 2 studies; Serious indirectness: variability in intervention	(18,19)
Lin 2019	Music listening at home vs. no intervention	Anxiety: STAI	393 (3)	SMD: -0.28 (-0.47, -0.08)	0%	Low	Very serious bias: All studies had overall high risk;	(12,14,18)
Lin 2019	Music listening during antepartum HDP hospitalization vs. no intervention	Anxiety: STAI	130 (2)	SMD: -0.54 (-2.09, 1.01)	94%	Very low	Very serious bias: Both studies had overall high risk; Very serious inconsistency: high I^2; Very serious imprecision: wide CIs that crossed the null, small sample size	(13,15)
Lin 2019	Music listening during antepartum procedures vs. no intervention	Anxiety: STAI	711 (3)	SMD: -0.43 (-1.21, 0.34)	96%	Very low	Very serious bias: All studies had overall high risk; very serious inconsistency: high I^2; serious indirectness: wide CIs that crossed the null	(11,16,17)
Lin 2019	Music listening with investigator chosen music vs. no intervention	Anxiety: STAI	445 (2)	SMD: -0.83 (-1.50, -0.17)	74%	Very low	Very serious bias: All studies had high risk; serious inconsistency: high I^2	(11,12)
Lin 2019	Music listening with patient chosen music vs. no intervention	Anxiety: STAI	789 (6)	SMD: -0.29 (-0.68, 0.09)	85%	Very low	Very serious bias: All studies had overall high risk; Very serious inconsistency: high I^2; Serious imprecision: wide CIs that crossed the null	(13–18)
Intrapartum Interventions								
Chuang 2019	Music listening during labor vs. no intervention	Anxiety: SAS	302 (4)	SMD: -0.96 (-1.15, -0.76)	0%	Low	Serious bias: All 3 studies have high risk of participant blinding;	(20–22)

Weingarten 2021	Music listening before cesarean delivery vs. no intervention	Preoperative anxiety: STAI	250 (4)	MD: -3.95 (- 9.07 to 1.18)	22%	Moderate	Very serious imprecision: wide CIs that crossed the null	(23–26)
Weingarten 2021	Music listening before cesarean delivery vs. no intervention	Preoperative anxiety: SAS	60 (1)	MD: -7 (- 8.43 to - 5.57)	NA	Low	Very serious imprecision: based on a single trial with wide CIs that crossed the null	(27)
Weingarten 2021	Music listening during cesarean delivery vs. no intervention	Intraoperative anxiety: VAS	368 (2)	MD: -0.54 (-0.87, -0.2)	0%	Moderate	Serious bias: 1 of 2 studies with high risk of blinding participant and outcomes;	(28,29)
Weingarten 2021	Music listening during cesarean delivery vs. no intervention	Intraoperative anxiety: SAS	64 (1)	MD: -4.8 (-7.08, -2.52)	NA	Low	Very serious imprecision: based on a single trial with wide CIs	(30)
Weingarten 2021	Music listening during cesarean delivery vs. no intervention	Intraoperative anxiety: STAI	304 (1)	MD: -2.8 (-4.57, -1.03)	NA	Low	Serious bias: High risk of participant and outcome blinding; Serious imprecision: based on a single trial with wide CIs	(29)
Weingarten 2021	Music listening during and immediately after cesarean delivery vs. no intervention	Postoperative anxiety: VAS	847 (7)	MD: -0.38 (- 0.53 to - 0.23)	0%	Moderate	Serious bias: 5 of 7 studies with high risk of participant blinding;	(28,29,31–35)
Weingarten 2021	Music listening before, during, and immediately after cesarean delivery vs. no intervention	Postoperative anxiety: STAI	504 (4)	MD: -2.1 (-6.38, - 2.18)	58%	Low	Serious bias: 2 of 4 studies with high risk of participant blinding; serious imprecision: wide CIs	(26,29,36, 37)
Weingarten 2021	Music listening during cesarean delivery vs. no intervention	Postoperative anxiety: SAS	64 (1)	MD: -4.5 (- 6.82 to- 2.18)	NA	Low	Very serious imprecision: based on a single trial with wide CIs	(30)
Weingarten 2021	Music listening before scheduled cesarean delivery vs. no intervention	Preoperative anxiety: STAI	125 (2)	MD: 0.63 (-4.62, 5.89)	48%	Low	Very serious imprecision: wide CIs that crossed the null, low sample size	(24,26)
Weingarten 2021	Music listening during and after scheduled cesarean delivery vs. no intervention	Postoperative anxiety: VAS	361 (5)	MD: -0.49 (-0.85, -0.13)	18%	Moderate	Serious bias: 3 of 5 studies with high risk of participant blinding;	(28,31,32, 34,35)
Weingarten 2021	Music listening before and during	Postoperative anxiety: STAI	200 (3)	MD: -2.22 (-7.44, 2.99)	15%	Low	Very serious imprecision: wide CIs that crossed the null, low sample size	(26,36,37)

	scheduled cesarean delivery vs. no intervention							
Postpartum interventions								
Hakimi 2021	Music listening and music therapy vs. no intervention	Anxiety 0.5-8hrs post-delivery: VAS-A	318 (3)	MD: -0.96 (-2.26, 0.34)	92%	Very low[a]	Very serious inconsistency: high I^2; Serious imprecision: total number of subjects less than 400	(32,38,39)
Hakimi 2021	Music listening and music therapy vs. no intervention	Anxiety 2 weeks postpartum: VAS-A	77 (1)	MD: 0.84 (-1.59, 3.27)	N/A	Very low[a]	Serious risk of bias; Very serious imprecision: wide CIs, sample less than 400	(40)
Yang 2019	Music listening vs. kangaroo care, traditional treatment, or psychological treatment	Anxiety: Composite continuous measure derived from: STAI, SAS, VAS	601 (3)	SMD: -1.26 (-2.81, 0.29)	98%	Very low	Serious indirectness: variability in the control and outcome measurement; Very serious inconsistency: high I^2; Serious imprecision: wide CIs that crossed the null	(38,41,42)
Yang 2019	Music listening vs. kangaroo care	Anxiety: Composite binary measure derived from STAI, SAS	460 (2)	RR: 0.38 (0.09, 1.63)	0%	Low	Serious indirectness: variability in the outcome measurement; wide CIs that crossed the null	(41,42)
Wu 2020	Five elements music listening alone or in combination with other treatment vs. no intervention	Anxiety rate	524 (2)	MD: 0.42 (0.19, 0.94)	0%	Low	Serious bias: most methodological details unclear; wide CIs that crossed the null	(41,43)
Acronyms: BAI: Beck Anxiety Inventory; CI: Confidence Intervals; DASS21: Depression, Anxiety and Stress Scale; MD: Mean difference; N/A: Not available; OR: Odds ratio; RR: Risk ratio; SAS: Self-rating Anxiety Scale; SMD: Standardized mean difference; STAI: State Anxiety Inventory (including STAI-S or STAI-T); VAS-A: Visual analogue scale for anxiety. [a] GRADE ratings were calculated by Hakimi 2021.								

References

1. Kızılkaya M, Karaahmet AY, Beydağ KD. The effect of music interventions on anxiety, pain, and pregnancy rates in women undergoing fertility treatment before oocyte collection and embryo transfer: Systematic review and meta-analysis of randomized controlled trials. Eur J Integr Med. 2024 Dec 1;72:102408.

2. Murphy EM, Nichols J, Somkuti SG, Sobel M, Braverman A, Barmat LI. Randomized Trial of Harp Therapy During In Vitro Fertilization–Embryo Transfer. J Evid-Based Complement Altern Med. 2014 Apr 1;19(2):93–8.

3. Fleury EA de B, Approbato MS, Barbosa MA. Interactive Music Therapy on Stress Level Reduction in Women Submitted to IVF/ICSI. Prospective Randomized Study. JBRA Assist Reprod. 2021 Apr 27;25(2):209–14.

4. Moragianni VA, Hopkins J, Somkuti SG, Lee A, Schinfeld JS, Barmat LI. Randomized trial of harp music therapy in IVF-ET. Fertil Steril. 2009 Sept 1;92(3, Supplement):S147–8.

5. Aba YA, Avci D, Guzel Y, Ozcelik SK, Gurtekin B. Effect of music therapy on the anxiety levels and pregnancy rate of women undergoing in vitro fertilization-embryo transfer: A randomized controlled trial. Appl Nurs Res. 2017 Aug 1;36:19–24.

6. Nandeibam Y, Joseph T, Antonisamy B, Kamath MS, Kunjummen AT. Effectiveness of music therapy as an adjuvant to conscious sedation in women undergoing transvaginal oocyte retrieval: A randomized controlled trial. J Obstet Gynaecol Res. 2022;48(6):1409–17.

7. Stocker L, Hardingham K, Cheong Y. A Randomized Controlled Trial Assessing Whether Listening to Music at Time of Embryo Transfer Effects Anxiety Levels. Gynecol Obstet. 2016 Sept 20;6.

8. Cheung CWC, Yee AWW, Chan PS, Saravelos SH, Chung JPW, Cheung LP, et al. The impact of music therapy on pain and stress reduction during oocyte retrieval – a randomized controlled trial. Reprod Biomed Online. 2018 Aug 1;37(2):145–52.

9. Orak Y, Bakacak SM, Yaylali A, Tolun FI, Kiran H, Boran OF, et al. Efeitos da musicoterapia sobre dor e estresse oxidativo na aspiração folicular: estudo clínico randomizado. Braz J Anesthesiol. 2020 Sept 1;70(5):491–9.

10. Lin CJ, Chang YC, Chang YH, Hsiao YH, Lin HH, Liu SJ, et al. Music Interventions for Anxiety in Pregnant Women: A Systematic Review and Meta-Analysis of Randomized Controlled Trials. J Clin Med. 2019 Nov 6;8(11):1884.

11. García González J, Ventura Miranda MI, Requena Mullor M, Parron Carreño T, Alarcón Rodriguez R. Effects of prenatal music stimulation on state/trait anxiety in full-term pregnancy and its influence on childbirth: a randomized controlled trial. J Matern Fetal Neonatal Med. 2018 Apr 18;31(8):1058–65.

12. Nwebube C, Glover V, Stewart L. Prenatal listening to songs composed for pregnancy and symptoms of anxiety and depression: a pilot study. BMC Complement Altern Med. 2017 May 8;17(1):256.

13. Toker E, Kömürcü N. Effect of Turkish classical music on prenatal anxiety and satisfaction: A randomized controlled trial in pregnant women with pre-eclampsia. Complement Ther Med. 2017 Feb;30:1–9.

14. Liu YH, Lee CS, Yu CH, Chen CH. Effects of music listening on stress, anxiety, and sleep quality for sleep-disturbed pregnant women. Women Health. 2016 Apr 2;56(3):296–311.

15. Cao S, Sun J, Wang Y, Zhao Y, Sheng Y, Xu A. Music therapy improves pregnancy-induced hypertension treatment efficacy. 2016 May 30;9:8833–8.

16. Guerrero JM, Casta□□o PM, Schmidt EO, Rosario L, Westhoff CL. Music as an auxiliary analgesic during first trimester surgical abortion: a randomized controlled trial. Contraception. 2012 Aug 1;86(2):157–62.

17. Kafalİ H, Derbent A, Keskİn E, Sİmavlİ S, Gözdemİr E. Effect of maternal anxiety and music on fetal movements and fetal heart rate patterns. J Matern Fetal Neonatal Med. 2011 Mar 1;24(3):461–4.

18. Chang MY, Chen CH, Huang KF. Effects of music therapy on psychological health of women during pregnancy. J Clin Nurs. 2008;17(19):2580–7.

19. Shin HS, Kim JH. Music Therapy on Anxiety, Stress and Maternal-fetal Attachment in Pregnant Women During Transvaginal Ultrasound. Asian Nurs Res. 2011 Mar;5(1):19–27.

20. Liu YH, Chang MY, Chen CH. Effects of music therapy on labour pain and anxiety in Taiwanese first-time mothers. J Clin Nurs. 2010;19(7–8):1065–72.

21. Phumdoung S, Good M. Music reduces sensation and distress of labor pain. Pain Manag Nurs Off J Am Soc Pain Manag Nurses. 2003 June;4(2):54–61.

22. Simavli S, Gumus I, Kaygusuz I, Yildirim M, Usluogullari B, Kafali H. Effect of Music on Labor Pain Relief, Anxiety Level and Postpartum Analgesic Requirement: A Randomized Controlled Clinical Trial. Gynecol Obstet Invest. 2014 Sept 16;78(4):244–50.

23. Sharifi A, Alipour A, Baharloei S. COMPARISON OF THE EFFECT OF INSTRUMENTAL MUSIC AND VOICES OF HOLY QURAN ON ANXIETY OF WOMAN BEFORE CESAREAN. J Urmia Nurs Midwifery Fac 2228-6411. 2013 Feb 1;10(6):841–6.

24. Kwun Y, Kim T. The Effect of Music Therapy on Anxiety of Cesarean Section Women. J Korean Acad Fundam Nurs. 2000;(7):466–78.

25. AjorPaz N, Ranjbar N. Effects of recitation of Holy Quran on anxiety of women before cesarean section: a randomize clinical trial. Qom Univ Med Sci J. 2010 Apr 10;4(1):15–9.

26. Parodi A, Fodde P, Pellecchia T, Puntoni M, Fracchia E, Mazzella M. A randomized controlled study examining a novel binaural beat technique for treatment of preoperative anxiety in a group of women undergoing elective caesarean section: Journal of Psychosomatic Obstetrics & Gynecology: Vol 42 , No 2 - Get Access. J Psychosom Obstet Gynecol. 2021;(42):147–51.

27. Li Y, Dong Y. Preoperative music intervention for patients undergoing cesarean delivery. Int J Gynecol Obstet. 2012 Oct 1;119(1):81–3.

28. Chang SC, Chen CH. Effects of music therapy on women's physiologic measures, anxiety, and satisfaction during cesarean delivery. Res Nurs Health. 2005;28(6):453–61.

29. Hepp P, Hagenbeck C, Gilles J, Wolf OT, Goertz W, Janni W, et al. Effects of music intervention during caesarean delivery on anxiety and stress of the mother a controlled, randomised study. BMC Pregnancy Childbirth. 2018 Dec;18(1):1–8.

30. Allameh T, JabalAmeli M, Lorestani K, Akbari M. The Efficacy of Quran Sound on Anxiety and Pain of Patients under Cesarean Section with Regional Anesthesia: A Randomized Case-Controlled Clinical Trial. J Isfahan Med Sch. 2013 June 22;31(235):601–10.

31. Reza N, Ali S, Saeed K, Abul-Qasim A, Reza T. The impact of music on postoperative pain and anxiety following cesarean section. Middle East J Anesthesiol. 2007;19(3):573–86.

32. Ebneshahidi A, Mohseni M. The Effect of Patient-Selected Music on Early Postoperative Pain, Anxiety, and Hemodynamic Profile in Cesarean Section Surgery. J Altern Complement Med. 2008 Sept;14(7):827–31.

33. Kurdi MS, Gasti V. Intraoperative Meditation Music as an Adjunct to Subarachnoid Block for the Improvement of Postoperative Outcomes Following Cesarean Section: A Randomized Placebo-controlled Comparative Study. Anesth Essays Res. 2018 Sept;12(3):618.

34. Bansal GL, Kaur H, Shukla V, Harsh HK, Gupta A. Music: an effective anxiolytic during caesarean section under spinal anaesthesia. Int J Res Med Sci. 2019 Feb 27;7(3):676–81.

35. Eren H, Sahiner N, Bal M, Dissiz M. Effects of music during multiple cesarean section delivery. J Coll Physicians Surg Pak. 2018;28(3):247–9.

36. Choubsaz M, Rezavand N, Bayat A, Farhadi K, Amirifard N. Comparison between the effect of ear plug and music in reducing anxiety in patients undergoing elective cesarean section under spinal anesthesia. Kuwait Med J. 2018;50(1):37–42.

37. Denney JM, Blackburn KL, Bleach CC, Martinez AR, Philips JB, Lanier K, et al. THE EFFECTS OF MUSIC INTERVENTION ON WOMEN'S ANXIETY BEFORE AND AFTER CESAREAN DELIVERY: A Randomized Controlled Trial. Music Med. 2018 Oct 28;10(4):225–32.

38. Simavli S, Kaygusuz I, Gumus I, Usluogulları B, Yildirim M, Kafali H. Effect of music therapy during vaginal delivery on postpartum pain relief and mental health. J Affect Disord. 2014 Mar;156:194–9.

39. Nikandish R, Ali S, Khademi S, Avand AQ, Reza T. The impact of music on postoperative pain and anxiety following cesarean section. Middle East J Anesthesiol. 2007 Oct 1;19:573–86.

40. Tseng YF, Chen CH, Lee CS. Effects of listening to music on postpartum stress and anxiety levels. J Clin Nurs. 2010 Apr;19(7–8):1049–55.

41. Wang W, Wang J, Qi X, Huang X, Jia J, Ren Q. Application of music in emotion management of puerpera after cesarean section. J Tradit Chin Med Manag. 2016;24(22):22–5.

42. Norouzi F, Keshavarz M, SeyedFatemi N, Montazeri A. The impact of kangaroo care and music on maternal state anxiety. Complement Ther Med. 2013 Oct 1;21(5):468–72.

43. Zou X. Effect observation of acupoint massage combined with five elements of music nursing intervention to promote postpartum lactation. J Sichuan Tradit Chin Med. 2018;36(3):181–3.
