# Supplementary material for: Music interventions to improve women’s health outcomes in the preconception, antepartum, intrapartum, and postpartum periods: An overview of reviews
Source: PLoS One. 2026 Feb 18;21(2):e0339337. doi: 10.1371/journal.pone.0339337 (PMC12915951; doi:10.1371/journal.pone.0339337)
Supplement: S9 Table — (PDF) [file pone.0339337.s009.pdf]

Supplementary Materials

Table S9: Summary of Effects of Music Interventions on Depression

Review	Comparison	Outcome measurement	No. of subjects (trials)	Effect (95% CI)	I^2 (%)	Quality of evidence (GRADE)	Comments	Primary studies
Antepartum interventions								
Sun 2024	Mixed music interventions vs. no intervention	Depression: Composite measure derived from EPDS, BDI, and HAM-D	703 (6)	SMD: -0.51 (-0.80, - 0.22)	67%	Very low	Serious bias: most methodological details unclear or high; Serious inconsistency: high I2; Serious indirectness: variability in the intervention and outcome measurement;	(1–6)
Han 2024	Mixed music interventions vs. no intervention	EPDS	374 (3)	SMD: -0.44 (-0.90, 0.02)	74%	Very low	Serious bias: most methodological details unclear or high; Serious inconsistency: high I2; Serious indirectness: variability in the intervention; Serious imprecision: wide CIs that crossed the null	(1,7,8)
Wu 2020	Five elements music listening along or in combination with other treatment vs. no treatment	Depression: SDS	1138 (6)	MD: -3.67 (-5.21, -2.13)	99%	Very low	Very serious inconsistency: high I2; Serious indirectness: variability in the intervention	(9–14)
Postpartum interventions								
Han 2024	Mixed music interventions vs. no intervention	Depression: EPDS	906 (6)	SMD: -0.59 (-0.96, -0.21)	87%	Very low	Serious bias: 3 of 6 studies with risk of participant blinding; Very serious inconsistency: high I2; Serious indirectness: variability in intervention; publication bias	(3–5,15–17)
Sun 2024	Music listening vs. no intervention	Depression: Composite measure derived from EPDS, BDI, and HAM-D	285 (4)	SMD: -0.75 (-1.47, -0.03)	87%	Very low	Serious bias: several studies with high risk in randomization, allocation concealment, and blinding; Very serious inconsistency: high I2; Serious indirectness: variability in intervention and outcome measurement;	(5,16–18)
Yang 2019	Music listening vs. psychological treatment and Chinese medicine	Depression: SDS	80 (1)	RR: 0.73 (0.63, 0.86)	N/A	Low	Serious bias: high or unclear on most methodological details; Serious imprecision: based on a single trial with low sample	(19)
Yang 2019	Music listening vs. psychological treatment, drug treatment, and/or traditional treatment	Depression: Composite continuous measure derived from EPDS, HAM-D, BAI, and SDS	763 (4)	SMD: -0.87 (-1.23, -0.51)	79%	Very low	Very serious inconsistency: high I2; Serious indirectness: variability in the control and outcome measurement;	(4,20–22)
Yang 2019	Music listening vs. health education, psychological treatment, and/or traditional treatment	Depression: Composite binary measure derived	623 (4)	RR: 0.25 (0.12, 0.54)	0%	Low	Serious indirectness: variability in the control and outcome measurement; Serious imprecision: wide CIs	(4,20,23)

		from EPDS and SDS						
Wu 2020	Five elements music listening alone or with psychiatric nursing vs. no intervention	Depression: EPDS	240 (2)	SMD: -0.84 (-1.33,-0.35)	71%	Low	Serious indirectness: variability in the intervention; Serious imprecision: wide CIs and low sample size	(24,25)
Acronyms: BDI: Beck Depression Inventory; CI: Confidence Intervals; EPDS: Edinburgh postnatal depression scale; HAM-D: Hamilton Depression Rating Scale; HIC: High income countries; LMIC: Low-and-middle income countries; N/A: Not available; RR: Risk ratio; SDS: Self-raiting depression scale; SMD: Standardized mean difference.								

References

1. Nwebube C, Glover V, Stewart L. Prenatal listening to songs composed for pregnancy and symptoms of anxiety and depression: a pilot study. BMC Complement Altern Med. 2017 May 8;17(1):256.

2. Cao S, Sun J, Wang Y, Zhao Y, Sheng Y, Xu A. Music therapy improves pregnancy-induced hypertension treatment efficacy. 2016 May 30;9:8833–8.

3. Chang MY, Chen CH, Huang KF. Effects of music therapy on psychological health of women during pregnancy. J Clin Nurs. 2008;17(19):2580–7.

4. Simavli S, Kaygusuz I, Gumus I, Usluogulları B, Yildirim M, Kafali H. Effect of music therapy during vaginal delivery on postpartum pain relief and mental health. J Affect Disord. 2014 Mar;156:194–9.

5. Wulff V, Hepp P, Wolf OT, Fehm T, Schaal NK. The influence of maternal singing on well-being, postpartum depression and bonding – a randomised, controlled trial. BMC Pregnancy Childbirth. 2021 July 12;21(1):501.

6. Qi W, Zhao F, Huang S, Wei Z, Yang H, He K, et al. Effects and Feasibility of a Mindfulness-Based Guqin Music Intervention During Pregnancy on Postpartum Anxiety and Depression: A Pilot Randomized Controlled Trial. Mindfulness. 2023 Nov 1;14(11):2641–56.

7. Palazzi A, Meschini R, Piccinini CA. NICU music therapy effects on maternal mental health and preterm infant's emotional arousal. Infant Ment Health J Infancy Early Child. 2021;42(5):672–89.

8. Wulff V, Hepp P, Wolf OT, Balan P, Hagenbeck C, Fehm T, et al. The effects of a music and singing intervention during pregnancy on maternal well-being and mother–infant bonding: a randomised, controlled study. Arch Gynecol Obstet. 2021 Jan 1;303(1):69–83.

9. Yanwei G, Zhiyun L, Jing R. Study on five- element music combined with herbal diet on the safety of late threatened abortion. J Qilu Nurs. 2016;22(20):6–8.

10. Jiawei L. Application of five elements music in the fetal heart monitoring. Med Innov China. 2016;13(17):120–2.

11. Zhen L. The clinical research of Zangshi Wuyin Ziangtiao acupuncture treatment for postpartum depression. Jilin J Tradit Chin Med. 2015;35(1):88–90.

12. Wenhui L. The effect of five elements music therapy based on traditional Chinese medicine theory on the anxiety and depression of pregnant women in perinatal period. Today Nurse. 2017;(12):58–60.

13. Wenjuan W. Application of five elements of traditional Chinese medicine music in maternal emotion management after cesarean section. J Tradit Chin Med Manag. 2016;(22):22–5.

14. Zhiyun L, Linlin Y, Yanqiong X. Effect of five elements music assisted interference on extended duration of pregnancy, maternal emotion and quality of life during the late threatened abortion. Nurs Pract Res. 2016;13(22):58–60.

15. Gaden TS, Ghetti C, Kvestad I, Bieleninik Ł, Stordal AS, Assmus J, et al. Short-term Music Therapy for Families With Preterm Infants: A Randomized Trial. Pediatrics. 2022 Jan 6;149(2):e2021052797.

16. Küçükkaya B, Can I, Güler G. The effect of music played to new birth mothers on postpartum blues: A randomized controlled trial. Early Hum Dev. 2024 May 1;192:106013.

17. Perkins R, Spiro N, Waddell G. Online songwriting reduces loneliness and postnatal depression and enhances social connectedness in women with young babies: randomised controlled trial. Public Health. 2023 July 1;220:72–9.

18. Ribeiro MKA, Alcântara-Silva TRM, Oliveira JCM, Paula TC, Dutra JBR, Pedrino GR, et al. Music therapy intervention in cardiac autonomic modulation, anxiety, and depression in mothers of preterms: randomized controlled trial. BMC Psychol. 2018 Dec 13;6(1):57.

19. Su Y. Clinical effect analysis of jieyuyangxuetang combined with music psychotherapy in the treatment of postpartum depression. Shenzhen J Integ Tradit Chin West Med. 2014;24(11):50–1.

20. Wang W, Wang J, Qi X, Huang X, Jia J, Ren Q. Application of music in emotion management of puerpera after cesarean section. J Tradit Chin Med Manag. 2016;24(22):22–5.

21. Huang C, Luo Z, Fang Z. Observation on the curative effect of drug combination with music psychotherapy for postpartum depression. Med Inf. 2010;23(3):636–7.

22. Lee SM. The Effects of Music Therapy on Postpartum Blues and Maternal Attachment of Puerperal Women. J Korean Acad Nurs. 2010 Feb 28;40(1):60–8.

23. Liu H. Application of music therapy in postpartum depression patients. Nurs Res Pract. 2014;11(6):63–4.

24. Chengshu W, Shuangfeng L, Wenjing X. Clinical effect of applying Chinese five elements music therapy on postpartum depression on the basis of psychological care. Chin J Conval Med. 2018;27(1):15–7.

25. Qiuyan W, Anzhou I, Sina I. Clinical study on the treatment of postpartum depression by traditional Chinese music. Lab Med Clin. 2013;10(13):1709–11.
