# Supplementary material for: Music interventions to improve women’s health outcomes in the preconception, antepartum, intrapartum, and postpartum periods: An overview of reviews
Source: PLoS One. 2026 Feb 18;21(2):e0339337. doi: 10.1371/journal.pone.0339337 (PMC12915951; doi:10.1371/journal.pone.0339337)
Supplement: S10 Table — (PDF) [file pone.0339337.s010.pdf]

Supplementary Materials

Table S10: Summary of effects of music interventions on pain

Review	Comparison	Outcome measurement	No. of subjects (trials)	Effect (95% CI)	I^2 (%)	Quality of evidence (GRADE)	Comments	Primary studies
Preconception interventions								
Kızılkaya 2024	Music listening before or during ART treatment vs. no intervention	Pain: VAS-P	302 (3)	MD: -0.96 (-1.72, -0.20)	0%	Low	Serious bias: all studies have high risk for blinding; Serious imprecision: wide CIs that crossed the null	(30,32,33)
Intrapartum interventions								
Chuang 2019	Music listening during labor vs. no intervention	Overall Pain: VAS-P	332 (4)	MD: -0.92 (-1.33, -0.51)	45%	Moderate	Serious bias: All studies have high risk of participant blinding, 1 study has high risk for randomization and allocation concealment;	(1–4)
Şen 2023	Music listening during labor vs. no intervention	Pain in active phase of labor: VAS-P	912 (16)	SMD: -1.26 (-1.52, -1.01)	67%	Moderate	Serious inconsistency: High I2;	(1,3,5–10)
Şen 2023	Music listening during labor vs. no intervention	Pain 2hrs after initiation of music: VAS-P	528 (7)	SMD: -1.22 (-1.67, -0.76)	81%	Low	Very serious inconsistency: High I2;	(1–3,5,7–9)
Şen 2023	Music listening during labor vs. no intervention	Pain 3hrs after initiative of music: VAS-P	312 (5)	SMD: -1.06 (-1.32, -0.81)	12%	High	Very serious inconsistency: High I2;	(2,5,7–9)
Şen 2023	Music listening during labor with preselected music vs. no intervention	Pain: VAS-P	860 (14)	SMD: -1.22 (-1.50, -0.95)	69%	Moderate	Serious inconsistency: High I2;	(2,5,7,9–11)
Şen 2023	Music listening during labor with headphones vs. no intervention	Pain: VAS-P	1418 (20)	SMD: -1.04 (-1.27, -0.81)	75%	Moderate	Serious inconsistency: High I2;	(1–3,5,7,8,11)
Şen 2023	Music listening during labor via speakers vs. no intervention	Pain: VAS-P	200 (4)	SMD: -0.44 (-0.93, 0.05)	65%	Low	Serious inconsistency: High I2; Serious imprecision: wide CIs that crossed the null, low sample;	(1,6,11)
Maleki 2023	Music listening after episiotomy vs. no intervention	Episiotomy pain measured at any time: VAS-P	677 (7)	SMD = -1.60 (-2.18, -1.02)	96%	Very Low	Serious bias: 4 of 7 studies have high risk for blinding; Very serious inconsistency: High I2;	(3,6,12–16)
Maleki 2023	Music listening after episiotomy vs. no intervention	Episiotomy pain 1hr post-procedure: VAS-P	425 (4)	SMD: -1.78 (-3.1, -0.46)	96%	Very low	Serious bias: 2 of 4 studies have high risk for blinding; Very serious inconsistency: High I2	(3,6,13,14)
Maleki 2023	Music listening after episiotomy vs. no intervention	Episiotomy pain 2hrs post-procedure: VAS-P	232 (2)	SMD: -2.7 (-5.21, -0.19)	98%	Very low	Very serious inconsistency: High I2; Very serious imprecision: wide CIs, small sample	(3,16)

Maleki 2023	Music listening after episiotomy vs. no intervention	Episiotomy pain 3-24hrs post-procedure: VAS-P	241 (2)	SMD: -2.09 (-2.71, -1.47)	88%	Very low	Very serious inconsistency: High I2; Very serious imprecision: wide CIs, small sample	(3,12,16)
Maleki 2023	Music listening after episiotomy vs. no intervention	Episiotomy pain 24hrs post-procedure: VAS-P	242 (2)	SMD: -1.71 (-4.49, 1.07)	98%	Very low	Very serious inconsistency: High I2; Very serious imprecision: wide CIs that crossed the null, small sample	(3,6)
Maleki 2023	Music listening after episiotomy vs. no intervention	Episiotomy pain 48hrs post-procedure: VAS-P	100 (1)	SMD: -0.16 (-0.88, 0.55)	NA	Very low	Serious bias: high risk for blinding; Very serious imprecision: based on a single trial with wide CIs, small sample	(6)
Maleki 2023	Music listening after episiotomy: high quality study vs. no intervention	Episiotomy pain: VAS-P	373 (3)	SMD: -1.6 (-2.18, -1.02)	79%	Low	Very serious inconsistency: High I2;	(3,12,14)
Maleki 2023	Music listening after episiotomy: low quality study vs. no intervention	Episiotomy pain: VAS-P	374 (4)	SMD:-0.55 (-1.01, -0.08)	86%	Very low	Serious bias: all studies have high risk for blinding, 1 study has high risk for randomization and allocation concealment; Very serious inconsistency: High I2;	(6,13,15,16)
Weingarten 2021	Music listening before, during, and immediately after cesarean delivery vs. no intervention	Postoperative pain: VAS-P	479 (5)	MD: -0.82 (-1.74, 0.11)	86%	Very low	Very serious inconsistency: high I2; Serious imprecision: wide CIs that crossed the null; Serious publication bias	(17–21)
Postpartum interventions								
Hakimi 2021	Music listening vs. no intervention	Postpartum Pain (30-60 mins after delivery): VAS-P	318 (3)	MD: -1.85 (-3.96, 0.26)	98%	Very low[a]	Very serious inconsistency: high I^2; Serious imprecision: wide CIs that crossed the null	(12,19,22)
Acronyms: ART: assisted reproductive technology; CI: Confidence Intervals; MD: Mean difference; N/A: Not available; RR: Risk ratio; SMD: Standardized mean difference; VAS-P: Visual analogue scale for pain.<br>[a] GRADE ratings were calculated by Hakimi 2021.								

References

1. Liu YH, Chang MY, Chen CH. Effects of music therapy on labour pain and anxiety in Taiwanese first-time mothers. J Clin Nurs. 2010;19(7–8):1065–72.

2. Phumdoung S, Good M. Music reduces sensation and distress of labor pain. Pain Manag Nurs Off J Am Soc Pain Manag Nurses. 2003 June;4(2):54–61.

3. Simavli S, Gumus I, Kaygusuz I, Yildirim M, Usluogullari B, Kafali H. Effect of Music on Labor Pain Relief, Anxiety Level and Postpartum Analgesic Requirement: A Randomized Controlled Clinical Trial. Gynecol Obstet Invest. 2014 Sept 16;78(4):244–50.

4. Hosseini SE, Bagheri M, Honarparvaran N. Investigating the effect of music on labor pain and progress in the active stage of first labor. Eur Rev Med Pharmacol Sci. 2013 June;17(11):1479–87.

5. Amanak K. The effect of the sound of the ney (reed flute) on women in labour in Bursa, Turkey. JPMA J Pak Med Assoc. 2020 Nov;70(11):1934–7.

6. Buglione A, Saccone G, Mas M, Raffone A, Di Meglio L, di Meglio L, et al. Effect of music on labor and delivery in nulliparous singleton pregnancies: a randomized clinical trial. Arch Gynecol Obstet. 2020 Mar 1;301(3):693–8.

7. Dehcheshmeh FS, Rafiei H. Complementary and alternative therapies to relieve labor pain: A comparative study between music therapy and Hoku point ice massage. Complement Ther Clin Pract. 2015 Nov;21(4):229–32.

8. Gönenç İM, Dikmen HA. Effects of Dance and Music on Pain and Fear During Childbirth. J Obstet Gynecol Neonatal Nurs JOGNN. 2020 Mar;49(2):144–53.

9. Gokyildiz Surucu S, Ozturk M, Avcibay Vurgec B, Alan S, Akbas M. The effect of music on pain and anxiety of women during labour on first time pregnancy: A study from Turkey. Complement Ther Clin Pract. 2018 Feb;30:96–102.

10. Suryani E, Sari LP, Natalia I. The Impact Langgams Music Instrumental of Javanese Style to Reduce Anxiety and Labor Pain. J Kesehat Masy. 2021 Mar 17;16(3):377–84.

11. Labrague LJ, Rosales RA, Rosales GL, Fiel GB. Effects of soothing music on labor pain among Filipino mothers. Clin Nurs Stud. 2013 Feb 22;1(1):35.

12. Simavli S, Kaygusuz I, Gumus I, Usluogulları B, Yildirim M, Kafali H. Effect of music therapy during vaginal delivery on postpartum pain relief and mental health. J Affect Disord. 2014 Mar;156:194–9.

13. Toker E, Gökduman Keleş M. The effect of virtual reality on fetal movement, fetal heart rate, maternal satisfaction, fatigue, and anxiety levels and vital signs of pregnant women during non-stress test: A randomized controlled trial. Health Care Women Int. 2024 July 2;45(7):765–81.

14. Şolt Kırca A, Kanza Gül D. The effect of music and skin contact with the newborn on pain and anxiety during episiotomy repair in primiparous women: A double-blind randomized controlled study. EXPLORE. 2022 Mar 1;18(2):210–6.

15. ANĞIN AD, oruç muhammet ali, öktem abdulmecit, Gursu T, ALAN Y, sakin onder, et al. The Effect of Music on Pain and Anxiety in Episiotomy. Ank Med J. 2020;20(3):541–52.

16. Chaichanalap R, Laosooksathit W, Kongsomboon K, Hanprasertpong T. Efficacy of Music Therapy on Immediate Postpartum Episiotomy Pain: A randomized controlled trial. Thai J Obstet Gynaecol. 2018 Sept 30;158–65.

17. Li Y, Dong Y. Preoperative music intervention for patients undergoing cesarean delivery. Int J Gynecol Obstet. 2012 Oct 1;119(1):81–3.

18. Reza N, Ali S, Saeed K, Abul-Qasim A, Reza T. The impact of music on postoperative pain and anxiety following cesarean section. Middle East J Anesthesiol. 2007;19(3):573–86.

19. Ebneshahidi A, Mohseni M. The Effect of Patient-Selected Music on Early Postoperative Pain, Anxiety, and Hemodynamic Profile in Cesarean Section Surgery. J Altern Complement Med. 2008 Sept;14(7):827–31.

20. Kurdi MS, Gasti V. Intraoperative Meditation Music as an Adjunct to Subarachnoid Block for the Improvement of Postoperative Outcomes Following Cesarean Section: A Randomized Placebo-controlled Comparative Study. Anesth Essays Res. 2018 Sept;12(3):618.

21. Eren H, Sahiner N, Bal M, Dissiz M. Effects of music during multiple cesarean section delivery. J Coll Physicians Surg Pak. 2018;28(3):247–9.

22. Nikandish R, Ali S, Khademi S, Avand AQ, Reza T. The impact of music on postoperative pain and anxiety following cesarean section. Middle East J Anesthesiol. 2007 Oct 1;19:573–86.
