# Supplementary material for: Music interventions to improve women’s health outcomes in the preconception, antepartum, intrapartum, and postpartum periods: An overview of reviews
Source: PLoS One. 2026 Feb 18;21(2):e0339337. doi: 10.1371/journal.pone.0339337 (PMC12915951; doi:10.1371/journal.pone.0339337)
Supplement: S11 Table — (PDF) [file pone.0339337.s011.pdf]

Supplementary Materials

Table S11: Summary of Effects of Music Interventions on Stress

Review	Comparison	Outcome measurement	No. of subjects (trials)	Effect (95% CI)	I^2 (%)	Quality of evidence (GRADE)	Comments	Primary studies
Antepartum interventions								
Corbijn van Willenswaard 2017	Music listening vs. no intervention	Stress: PSS	532 (2)	SMD: -0.08 (-0.25, 0.09)	0%	Moderate	Serious imprecision: wide confidence interval that crossed the null	(1,2)
Corbijn van Willenswaard 2017	Music listening vs. no intervention	Stress: Pregnancy Stress Rating Scale	529 (2)	SMD: -0.02 (-0.19, 0.15)	0%	Low	Serious bias: High risk in sequence generation, allocation concealment and blinding in 1 of 2 studies; Serious imprecision: wide confidence interval that crossed the null	(2,3)
Acronyms: CI: Confidence Intervals; PSS: Perceived stress rating scale; SDS: Self-raiting depression scale; SMD: Standardized mean difference.								

References

1. Chang MY, Chen CH, Huang KF. Effects of music therapy on psychological health of women during pregnancy. J Clin Nurs. 2008;17(19):2580–7.

2. Chang HC, Yu CH, Chen SY, Chen CH. The effects of music listening on psychosocial stress and maternal–fetal attachment during pregnancy. Complement Ther Med. 2015 Aug 1;23(4):509–15.

3. Shin HS, Kim JH. Music Therapy on Anxiety, Stress and Maternal-fetal Attachment in Pregnant Women During Transvaginal Ultrasound. Asian Nurs Res. 2011 Mar;5(1):19–27.
