# Supplementary material for: Music interventions to improve women’s health outcomes in the preconception, antepartum, intrapartum, and postpartum periods: An overview of reviews
Source: PLoS One. 2026 Feb 18;21(2):e0339337. doi: 10.1371/journal.pone.0339337 (PMC12915951; doi:10.1371/journal.pone.0339337)
Supplement: S12 Table — (PDF) [file pone.0339337.s012.pdf]

Supplementary Materials

Table S12: Summary of Effects of Music Interventions on Sleep

Review	Comparison	Outcome measurement	No. of subjects (trials)	Effect (95% CI)	I^2 (%)	Quality of evidence (GRADE)	Comments	Primary studies
Antepartum interventions								
Hoffmann 2025	Music listening vs. no intervention	Sleep: PSQI	348 (4)	MD -1.38 (-2.56, -0.19)	76%	Very low	Serious bias: all studies have high or unclear risk for blinding; Very serious inconsistency: high I^2	(1–4)
Postpartum interventions								
Yang 2019	Music listening vs. no intervention	Sleep: PSQI	82 (1)	MD: -2.30 (-2.75, -1.85)	N/A	Low	Very serious imprecision: based on a single trial with wide CIs, small sample	(5)
Acronyms: CI: Confidence Intervals; MD: Mean difference; N/A: Not applicable; PSQI: Pittsburgh Sleep Quality Index.								

References

1. Liu YH, Lee CS, Yu CH, Chen CH. Effects of music listening on stress, anxiety, and sleep quality for sleep-disturbed pregnant women. Women Health. 2016 Apr 2;56(3):296–311.

2. Shobeiri F, Khaledi S, Masoumi SZ, Roshanaei G. The effect of music therapy counseling on sleep quality in pregnant women | Abstract. [cited 2025 July 5]; Available from: https://www.ijmrhs.com/abstract/the-effect-of-music-therapy-counseling-on-sleep-quality-in-pregnant-women-6782.html

3. Sanlı Y, Arslan GG, Akbag NNA, Sahiner NC, Yılmaz D, Yucel SC. Effects of music on sleep quality and comfort levels of pregnant women. J Perinat Med. 2022 May 1;50(4):467–75.

4. Hoegholt NF, Krænge CE, Vuust P, Kringelbach M, Jespersen KV. Music and Sleep Hygiene Interventions for Pregnancy-Related Insomnia: An Online Randomized Controlled Trial. J Midwifery Womens Health. 2025;70(3):387–95.

5. Liu H. Application of music therapy in postpartum depression patients. Nurs Res Pract. 2014;11(6):63–4.
