# Supplementary material for: Music interventions to improve women’s health outcomes in the preconception, antepartum, intrapartum, and postpartum periods: An overview of reviews
Source: PLoS One. 2026 Feb 18;21(2):e0339337. doi: 10.1371/journal.pone.0339337 (PMC12915951; doi:10.1371/journal.pone.0339337)
Supplement: S13 Table — (PDF) [file pone.0339337.s013.pdf]

Supplementary Materials

Table S13: Summary of Effects of Music Interventions on Other Outcomes

Review	Comparison	Outcome measurement	No. of subjects (trials)	Effect (95% CI)	I^2 (%)	Quality of evidence (GRADE)	Comments	Primary studies
Preconception interventions								
Kızılkaya 2024	Mixed music interventions before or during ART treatment vs. no intervention	Pregnancy rate	409 (3)	MD= -0.14 (-2.63, 2.34)	86%	Very low	Serious bias: high risk for blinding in all studies; Very serious inconsistency: high I^2; Serious indirectness: variability in intervention; Serious imprecision: wide confidence intervals that cross the line of no effect	(1–3)
Intrapartum interventions								
Şen 2023	Listening to music during labor vs. no intervention	Total labor duration: mins	30 (1)	MD: -22.95 (-56.90, 11.00)	n/a	Low	Very serious imprecision: based on a single trial with wide confidence intervals, small sample	(4)
Şen 2023	Listening to music during labor vs. no intervention	Duration of first stage of labor: mins	60 (1)	MD: 64.20 (-41.63, 170.03)	n/a	Low	Very serious imprecision: based on a single trial with wide confidence intervals, small sample	(5)
Weingarten 2021	Music listening during and immediately after cesarean delivery vs. no intervention	Postoperative opioid use	177 (2)	MD: -0.87 (-1.55, -0.19)	0%	Moderate	Serious imprecision: wide confidence intervals, small sample size	(6,7)
Postpartum Interventions								
Düzgün 2020	Mixed music interventions vs. no intervention	Breast milk production: milliliters	554 (5)	Hedge's g = 0. 39 (0.18, 0.61)	21%	Moderate	Serious imprecision: wide confidence intervals that crossed the null	(8–12)
Yang 2019	Music listening vs. no intervention	Satisfaction: VAS	141 (1)	MD: 2.92 (2.67, 3.17)	NA	Low	Very serious imprecision: based on a single trial with wide confidence intervals, small sample	(13)
Yang 2019	Music listening vs. no intervention	Maternal attachment: Maternal attachment tool	60 (1)	MD: 3.30 (1.72, 4.88)	NA	Low	Very serious imprecision: based on a single trial with wide confidence intervals, small sample	(14)
Acronyms: ART: assisted reproductive technology; CI: Confidence Intervals; MD: Mean difference.								

References:

1. Murphy EM, Nichols J, Somkuti SG, Sobel M, Braverman A, Barmat LI. Randomized Trial of Harp Therapy During In Vitro Fertilization–Embryo Transfer. J Evid-Based Complement Altern Med. 2014 Apr 1;19(2):93–8.

2. Aba YA, Avci D, Guzel Y, Ozcelik SK, Gurtekin B. Effect of music therapy on the anxiety levels and pregnancy rate of women undergoing in vitro fertilization-embryo transfer: A randomized controlled trial. Appl Nurs Res. 2017 Aug 1;36:19–24.

3. Stocker L, Hardingham K, Cheong Y. A Randomized Controlled Trial Assessing Whether Listening to Music at Time of Embryo Transfer Effects Anxiety Levels. Gynecol Obstet. 2016 Sept 20;6.

4. Amanak K. The effect of the sound of the ney (reed flute) on women in labour in Bursa, Turkey. JPMA J Pak Med Assoc. 2020 Nov;70(11):1934–7.

5. Kimber L, McNabb M, Mc Court C, Haines A, Brocklehurst P. Massage or music for pain relief in labour: a pilot randomised placebo controlled trial. Eur J Pain Lond Engl. 2008 Nov;12(8):961–9.

6. Reza N, Ali S, Saeed K, Abul-Qasim A, Reza T. The impact of music on postoperative pain and anxiety following cesarean section. Middle East J Anesthesiol. 2007;19(3):573–86.

7. Ebneshahidi A, Mohseni M. The Effect of Patient-Selected Music on Early Postoperative Pain, Anxiety, and Hemodynamic Profile in Cesarean Section Surgery. J Altern Complement Med. 2008 Sept;14(7):827–31.

8. Vianna MNS, Barbosa AP, Carvalhaes AS, Cunha AJLA. Music therapy may increase breastfeeding rates among mothers of premature newborns: a randomized controlled trial. J Pediatr (Rio J). 2011 June 8;87(3):206–12.

9. Dabas S, Joshi P, Agarwal R, Yadav RK, Kachhawa G. Impact of audio assisted relaxation technique on stress, anxiety and milk output among postpartum mothers of hospitalized neonates: A randomized controlled trial. J Neonatal Nurs. 2019 Aug 1;25(4):200–4.

10. AK J, Lakshmanagowda PB, G C M P, Goturu J. Impact of Music Therapy on Breast Milk Secretion in Mothers of Premature Newborns. J Clin Diagn Res JCDR. 2015 Apr;9(4):CC04–6.

11. Kittithanesuan Y, Chiarakul S, Kaewkungwal J, Poovorawan Y. Effect of music on immediately postpartum lactation by term mothers after giving birth: A randomized controlled trial. J Med Assoc Thai. 2017 Aug;100(8):834–42.

12. Mohd Shukri NH, Wells J, Eaton S, Mukhtar F, Petelin A, Jenko-Pražnikar Z, et al. Randomized controlled trial investigating the effects of a breastfeeding relaxation intervention on maternal psychological state, breast milk outcomes, and infant behavior and growth. Am J Clin Nutr. 2019 July 1;110(1):121–30.

13. Simavli S, Kaygusuz I, Gumus I, Usluogulları B, Yildirim M, Kafali H. Effect of music therapy during vaginal delivery on postpartum pain relief and mental health. J Affect Disord. 2014 Mar;156:194–9.

14. Lee SM. The Effects of Music Therapy on Postpartum Blues and Maternal Attachment of Puerperal Women. J Korean Acad Nurs. 2010 Feb 28;40(1):60–8.
