# Supplementary material for: Music interventions to improve women’s health outcomes in the preconception, antepartum, intrapartum, and postpartum periods: An overview of reviews
Source: PLoS One. 2026 Feb 18;21(2):e0339337. doi: 10.1371/journal.pone.0339337 (PMC12915951; doi:10.1371/journal.pone.0339337)
Supplement: S14 Table — (PDF) [file pone.0339337.s014.pdf]

Supplementary Materials

Table S14: Summary of Effects of Music Interventions on Vital Signs

Review	Comparison	Outcome measurement	No. of subjects (trials)	Effect (95% CI)	I^2 (%)	Quality of evidence (GRADE)	Comments	Primary studies
Preconception interventions								
Kızılkaya 2024	Mixed music interventions before or during ART treatment vs. no intervention	Heart rate	369 (3)	MD: 0.56 (-1.49, 2.61)	27%	Very low	Serious bias: 2 of 3 studies with high risk for blinding; Serious indirectness: variable interventions; Serious imprecision: wide confidence intervals that crossed the null	(1–3)
Kızılkaya 2024	Harp music therapy with embryo transfer vs. no intervention	Respiratory rate	307 (2)	MD: -0.24 (-0.81, 0.34)	0%	Low	Serious bias: high or unclear risk for blinding in both studies; Serious imprecision: wide confidence intervals that crossed the null	(1,2)
Kızılkaya 2024	Harp music therapy with embryo transfer vs. no intervention	Blood pressure	307 (2)	MD: -1.92 (-4.24, 0.40)	0%	Low	Serious bias: high or unclear risk for blinding in both studies; Serious imprecision: wide confidence intervals that crossed the null	(1,2)
Intrapartum interventions								
Weingarten 2021	Music listening before, during, and immediately after cesarean delivery vs. no intervention	Postoperative blood pressure: systolic	620 (6)	MD: -0.21 (-2.25, 1.84)	0%	Low	Serious bias: 5 of 6 studies with high risk of participant blinding; Serious imprecision: wide confidence intervals that cross the line of no effect	(4–9)
Weingarten 2021	Music listening before, during, and immediately after cesarean delivery vs. no intervention	Postoperative blood pressure: diastolic	620 (6)	MD: -0.85 (-2.39, 0.69)	0%	Low	Serious bias: 5 of 6 studies with high risk of participant blinding; Serious imprecision: wide confidence intervals that cross the line of no effect	(4–9)
Weingarten 2021	Music listening before, during, and immediately after cesarean delivery vs. no intervention	Postoperative heart rate	744 (8)	MD: -2.3 (-5.65, 1.04)	74%	Low	Serious bias: 6 of 8 studies with high risk of participant blinding; Serious imprecision: wide confidence intervals that cross the line of no effect	(4–11)
Acronyms: ART: assisted reproductive technology; CI: Confidence Intervals; MD: Mean difference.								

References

1. Murphy EM, Nichols J, Somkuti SG, Sobel M, Braverman A, Barmat LI. Randomized Trial of Harp Therapy During In Vitro Fertilization–Embryo Transfer. J Evid-Based Complement Altern Med. 2014 Apr 1;19(2):93–8.

2. Moragianni VA, Hopkins J, Somkuti SG, Lee A, Schinfeld JS, Barmat LI. Randomized trial of harp music therapy in IVF-ET. Fertil Steril. 2009 Sept 1;92(3, Supplement):S147–8.

3. Orak Y, Bakacak SM, Yaylali A, Tolun FI, Kiran H, Boran OF, et al. Efeitos da musicoterapia sobre dor e estresse oxidativo na aspiração folicular: estudo clínico randomizado. Braz J Anesthesiol. 2020 Sept 1;70(5):491–9.

4. Kwun Y, Kim T. The Effect of Music Therapy on Anxiety of Cesarean Section Women. J Korean Acad Fundam Nurs. 2000;(7):466–78.

5. Chang SC, Chen CH. Effects of music therapy on women's physiologic measures, anxiety, and satisfaction during cesarean delivery. Res Nurs Health. 2005;28(6):453–61.

6. Hepp P, Hagenbeck C, Gilles J, Wolf OT, Goertz W, Janni W, et al. Effects of music intervention during caesarean delivery on anxiety and stress of the mother a controlled, randomised study. BMC Pregnancy Childbirth. 2018 Dec;18(1):1–8.

7. Ebneshahidi A, Mohseni M. The Effect of Patient-Selected Music on Early Postoperative Pain, Anxiety, and Hemodynamic Profile in Cesarean Section Surgery. J Altern Complement Med. 2008 Sept;14(7):827–31.

8. Eren H, Sahiner N, Bal M, Dissiz M. Effects of music during multiple cesarean section delivery. J Coll Physicians Surg Pak. 2018;28(3):247–9.

9. Denney JM, Blackburn KL, Bleach CC, Martinez AR, Philips JB, Lanier K, et al. THE EFFECTS OF MUSIC INTERVENTION ON WOMEN'S ANXIETY BEFORE AND AFTER CESAREAN DELIVERY: A Randomized Controlled Trial. Music Med. 2018 Oct 28;10(4):225–32.

10. Allameh T, JabalAmeli M, Lorestani K, Akbari M. The Efficacy of Quran Sound on Anxiety and Pain of Patients under Cesarean Section with Regional Anesthesia: A Randomized Case-Controlled Clinical Trial. J Isfahan Med Sch. 2013 June 22;31(235):601–10.

11. Bansal GL, Kaur H, Shukla V, Harsh HK, Gupta A. Music: an effective anxiolytic during caesarean section under spinal anaesthesia. Int J Res Med Sci. 2019 Feb 27;7(3):676–81.
